# Household concentrations and female and child exposures to air pollution in peri-urban sub-Saharan Africa: measurements from the CLEAN-Air(Africa) study

**DOI:** 10.1016/S2542-5196(23)00272-3

**Published:** 2024-02-06

**Authors:** Matthew Shupler, Theresa Tawiah, Emily Nix, Miranda Baame, Federico Lorenzetti, Emmanuel Betang, Ryan Chartier, Judith Mangeni, Adithi Upadhya, Rachel Anderson de Cuevas, Edna Sang, Ricardo Piedrahita, Michael Johnson, Daniel Wilson, Seeba Amenga-Etego, Mieks Twumasi, Sara Ronzi, Diana Menya, Elisa Puzzolo, Reginald Quansah, Kwaku Poku Asante, Daniel Pope, Bertrand Hugo Mbatchou Ngahane

**Affiliations:** a Department of Public Health, Policy and Systems, University of Liverpool, Liverpool, UK; b Kintampo Health Research Centre, Kintampo, Ghana; c Douala General Hospital, Douala, Cameroon; d RTI International, Research Triangle Park, NC, USA; e School of Public Health, Moi University, Eldoret, Kenya; f Berkeley Air Monitoring Group, Berkeley, CA, USA; g Geocene, Berkeley, CA, USA; h School of Public Health, University of Ghana, Accra, Ghana

## Abstract

**Background:**

Relatively clean cooking fuels such as liquefied petroleum gas (LPG) emit less fine particulate matter (PM_2·5_) and carbon monoxide (CO) than polluting fuels (eg, wood, charcoal). Yet, some clean cooking interventions have not achieved substantial exposure reductions. This study evaluates determinants of between-community variability in exposures to household air pollution (HAP) across sub-Saharan Africa.

**Methods:**

In this measurement study, we recruited households cooking primarily with LPG or exclusively with wood or charcoal in peri-urban Cameroon, Ghana, and Kenya from previously surveyed households. In 2019–20, we conducted monitoring of 24 h PM_2·5_ and CO kitchen concentrations (n=256) and female cook (n=248) and child (n=124) exposures. PM_2·5_ measurements used gravimetric and light scattering methods. Stove use monitoring and surveys on cooking characteristics and ambient air pollution exposure (eg, walking time to main road) were also administered.

**Findings:**

The mean PM_2·5_ kitchen concentration was five times higher among households cooking with charcoal than those using LPG in the Kenyan community (297 μg/m^3^, 95% CI 216–406, *vs* 61 μg/m^3^, 49–76), but only 4 μg/m^3^ higher in the Ghanaian community (56 μg/m^3^, 45–70, *vs* 52 μg/m^3^, 40–68). The mean CO kitchen concentration in charcoal-using households was double the WHO guideline (6·11 parts per million [ppm]) in the Kenyan community (15·81 ppm, 95% CI 8·71–28·72), but below the guideline in the Ghanaian setting (1·77 ppm, 1·04–2·99). In all communities, mean PM_2·5_ cook exposures only met the WHO interim-1 target (35 μg/m^3^) among LPG users staying indoors and living more than 10 min walk from a road.

**Interpretation:**

Community-level variation in the relative difference in HAP exposures between LPG and polluting cooking fuel users in peri-urban sub-Saharan Africa might be attributed to differences in ambient air pollution levels. Thus, mitigation of indoor and outdoor PM_2·5_ sources will probably be critical for obtaining significant exposure reductions in rapidly urbanising settings of sub-Saharan Africa.

**Funding:**

UK National Institute for Health and Care Research.

## Introduction

About 3 billion people in low-income and middle-income countries cook with polluting fuels (eg, wood, charcoal) and are exposed to dangerous levels of household air pollution (HAP).^1^ Fine particulate matter (PM_2·5_) and carbon monoxide (CO) are health-relevant pollutants found in HAP.^2^ Exposure to PM_2·5_ from HAP is estimated to cause 2·6 million premature deaths annually,^3^ and it has been epidemiologically linked with several adverse effects, including respiratory diseases (chronic obstructive pulmonary disease, acute lower respiratory infection, lung cancer),^4^, ^5^, ^6^ cataracts,^7^, ^8^ elevated blood pressure,^9^, ^10^ and cardiovascular diseases (ischaemic heart disease, stroke).^7^, ^11^, ^12^, ^13^ Among children, PM_2·5_ exposure can lead to pneumonia and other pulmonary diseases.^13^ CO exposure can potentially contribute to asthma, cardiovascular diseases, and neurological impairment.^14^ WHO has therefore established Indoor Air Quality Guidelines for these two pollutants to protect public health.^15^

Transitioning from polluting to cleaner cooking fuels (eg, liquefied petroleum gas [LPG], electricity, ethanol) can reduce PM_2·5_ and CO exposures.^16^ However, previous clean cooking interventions conducted in urban sub-Saharan Africa have found that PM_2·5_ kitchen levels remained above the WHO interim-1 target level (35 μg/m^3^) when cleaner fuels were used.^17^ Other studies have uncovered that HAP levels are affected by environmental characteristics, including ventilation and ambient air pollution infiltrating indoors from sources including trash burning^18^ and traffic.^19^ There is further evidence from China^20^ and India^21^ that ambient PM_2·5_ sources affect personal exposure levels in communities reliant on polluting cooking fuels.^22^, ^23^

**Research in context**

**Evidence before this study**

An estimated 3 billion people cook with highly polluting fuels (eg, wood, coal, animal dung, kerosene). Household air pollution (HAP) measurement studies demonstrate that cooking with polluting fuels is associated with higher levels of fine particulate matter (PM_2·5_) and carbon monoxide (CO; two key indicators of health impacts) than cooking with cleaner cooking fuels (eg, liquefied petroleum gas [LPG], ethanol, electricity). Our literature search on PubMed in October, 2022, identified published intervention studies assessing HAP exposure differences between polluting and clean cooking fuels in sub-Saharan Africa. Keywords of “sub-Saharan Africa”, “HAP”, “clean cooking”, “clean fuels”, “polluting fuels”, “solid fuels”, “PM_2·5_”, “CO”, “intervention” and “randomized controlled trial” were included. Three intervention studies published in English were identified. The intervention studies reported large variations in the degree by which HAP exposures differ between households cooking with cleaner fuels and polluting fuels. Thus, detailed examinations of drivers of HAP exposure disparities across different settings are needed, especially in sub-Saharan Africa.

**Added value of study**

This multisite measurement study is one of the largest in sub-Saharan Africa to integrate HAP monitoring, stove use monitoring, and survey data. Advancements in monitoring technology enabled an examination of variations in PM_2·5_ and CO exposures during cooking and non-cooking periods and in relation to indicators of ambient exposure (eg, travel time to main road, number of times leaving the home) across urbanising communities in Cameroon (Mbalmayo), Ghana (Obuasi), and Kenya (Eldoret). We found that cooking with polluting fuels was a major PM_2·5_ exposure source in the Cameroonian and Kenyan communities but not the Ghanaian setting. Greater ambient air pollution exposure among cooks using LPG relative to that of polluting fuel users in the Ghanaian community might be negating PM_2·5_ exposure reductions associated with clean cooking fuel use.

**Implications of all the available evidence**

Although switching to LPG for cooking has potential benefits to health by lowering HAP exposure, our results demonstrate that the public health benefits from a transition from polluting cooking fuels to LPG for cooking in sub-Saharan Africa might be unequal across communities because of differences in localised levels of ambient air pollution. Thus, interventions are needed to mitigate both indoor and outdoor air pollution sources in areas with high ambient air pollution to achieve meaningful PM_2·5_ exposure reductions, particularly as sub-Saharan Africa continues to urbanise.

The most recent report of the *Lancet* Commission on pollution and health noted reduced mortality from HAP and a simultaneous increase in deaths attributable to ambient air pollution over the past two decades in sub-Saharan Africa.^24^ We therefore aimed to descriptively compare variations in HAP levels between cooking and non-cooking periods and by underlying community characteristics (eg, ambient air pollution levels, cooking location) and by integrating stove use monitoring^25^ with PM_2·5_ and CO measurements across peri-urban Cameroon, Kenya, and Ghana. By identifying environmental determinants of between-community variation in HAP levels, we evaluated whether transitioning to clean cooking fuels might lead to varied levels of reductions in HAP exposure (and thus differential health benefits) across peri-urban settings in sub-Saharan Africa. Such information will be useful for policy makers seeking to efficiently allocate limited resources to have the greatest global health benefit.

## Methods

### Study areas

CLEAN-Air(Africa) was a three-phased study carried out in three peri-urban communities in sub-Saharan Africa. Peri-urban communities were defined as rapidly urbanising areas adjacent to urban centres, and they were selected because of a greater mix of clean and polluting cooking fuel-using households.^26^ A sufficient variety of cooking fuels in the community was necessary to establish a sufficient sample size for comparing HAP levels between fuel groups.

The communities included in the study were in Cameroon (Mbalmayo), Ghana (Obuasi), and Kenya (Eldoret; appendix p 3). Mbalmayo is an agricultural town with 60 000 residents and is an hour drive away from Yaoundé, the country's capital. Obuasi is a gold-mining community in the southern Ashanti region, with a population of almost 200 000, which is an hour drive away from Kumasi (capital city of the Ashanti region). Eldoret is surrounded by agricultural land and sits at an elevation of more than 2000 m in western Kenya, with a population of nearly 500 000.

### Study design

In phase 1 of the study, we used door-to-door sampling to randomly select 2000 households within each community; primary cooks from these households were surveyed on their cooking patterns.^27^ In phase 2, the corresponding author (MS) conducted stratified random sampling (using the sampling function in R) to select primary cooks from phase 1 who cooked either primarily with LPG (n=200) or exclusively with polluting fuels (n=200) from each study site (total n=1200); phase 2 participants were surveyed on cooking behaviours and wellbeing.^28^ In phase 3, the focus of this study, stratified random sampling was used to select an equal number of phase 2 participants (female participants aged ≥18 years only) who were cooking either primarily with LPG or exclusively with polluting fuels in each community. Female primary cooks who smoked (eight [1%] of 1150) or households that used kerosene for lighting (36 [3%]) in the phase 2 survey were excluded from phase 3.

This study received ethics approval from the University of Liverpool Health and Life Sciences Research Ethics Committee (reference numbers: 4594 [Cameroon], 4811 [Kenya], 5298 [Ghana]), Central Regional Ethics Committee for Human Health Research (Cameroon; CRERSHC 846), Institutional Research and Ethics Committee for Moi Teaching and Referral Hospital and Moi University (Kenya; IREC 3298), and Kintampo Health Research Centre (Ghana; KHRCIEC 4854). Informed written consent was obtained from all participants by the interviewer before the start of data collection.

### Monitoring of HAP and stove use

A total of 40 households using LPG and 40 households cooking exclusively with polluting fuels was targeted for HAP monitoring (phase 3) per site in Mbalmayo and Obuasi. In Eldoret, a larger sample size of 50 households using LPG and 50 households cooking only with polluting fuels was targeted due to a collaboration with another research partner that required a greater sample size to simultaneously conduct measurements of emissions and living area HAP monitoring.^29^ Thus, the sampling period in Eldoret (5 months) was longer than in the other two communities (2–3 months).

HAP monitoring included simultaneous 24 h monitoring of kitchen PM_2·5_ concentration and personal exposure to PM_2·5_ (personal exposure for primary cook and child in Eldoret and Obuasi and for primary cook only in Mbalmayo). Child exposure measurements were not collected in some households in Obuasi and Eldoret because of the child being in preschool or the monitor being too heavy or noisy (44 [26%] of 172 households); only PM_2·5_ kitchen concentration and cook exposure monitoring occurred. A maximum of one child was monitored per household.

Real-time quality control of incoming data enabled re-monitoring in some households to maximise the number of valid HAP samples; however, outstanding issues with monitoring equipment (battery died early [n=6], pump malfunction [n=1], damaged filter [n=1]) led to PM_2·5_ cook exposures being collected in 248 (97%) of 256 households with PM_2·5_ kitchen concentration measurements.

CO kitchen and cook monitoring were also conducted concurrently with PM_2·5_ sampling. Because of a lower availability of CO monitors due to budget constraints, CO measurements were not collected among children in Eldoret and Obuasi. HAP monitoring was conducted in Mbalmayo from June to August, 2019, Eldoret from September, 2019, to January, 2020, and Obuasi from February to April, 2020.

Kitchen PM_2·5_ concentrations and cook exposures were collected using a lightweight micro personal exposure monitor (MicroPEM; RTI International, Research Triangle Park, NC, USA). The MicroPEM collects gravimetric and real-time PM_2·5_ measurements at 10 s intervals using a nephelometer at a flow rate of 0·40 L/min with a 25 mm PTFE filter. Child exposures were measured using the enhanced children's MicroPEM (ECM) monitor (RTI International). The ECM operates at a flow rate of 0·3 L/min, uses 15 mm PTFE filters, and also measures real-time particle concentrations. The MicroPEM and ECM also measure triaxial accelerometry, which served as a proxy for participant wearing compliance.

Seven to eight (10% of total sample) field-blank filters were collected in each community (total n=23) by placing a filter in the microPEM or ECM without turning on the pump and bringing the monitor to a household. All filters were pre-weighed and post-weighed in triplicate using the same balance system maintained in a temperature-controlled and humidity-controlled laboratory in Research Triangle Park (method detection limit: 3·2 μg/m^3^; instrumental limit of detection 1·2 μg/m^3^). The average weight of the filter blanks was subtracted from the net weight of each filter to calculate the PM_2·5_ mass on each filter.

In Eldoret and Obuasi, 24 h ambient PM_2·5_ measurements were collected with the MicroPEM in at least four locations deemed safe to leave monitoring equipment overnight. In Eldoret, ambient PM_2·5_ concentrations were collected in a single location with the Ultrasonic Personal Aerosol Sampler (UPAS; Access Sensor Technologies, Fort Collins, CO, USA).^29^ A total of 25 ambient PM_2·5_ measurements were conducted across the three study communities (four in Mbalmayo, ten in Obuasi, and 11 in Eldoret; appendix p 37).

CO kitchen measurements were obtained using the EL-USB-CO monitor (Lascar Electronics, Wiltshire, UK). Before use, three CO monitors were calibrated by Thermosense (Bourne End, UK) using span gas to UK Accreditation Service standards at a concentration of 100–500 parts per million (ppm). In each community, CO monitors were deployed in the same household to establish correction factors (ranging from 0·8 to 1·2) between the three calibrated monitors and the 15 remaining monitors. The appendix (pp 5–7) shows additional information on monitor placement (including pictures) and filter analysis.

Phase 3 households had Geocene Dot temperature sensors (Geocene, Berkeley, CA, USA)^25^ placed on all household cookstoves for stove use monitoring (SUM) during the 24 h HAP monitoring period. The SUMs recorded a temperature measurement every 5 min, which was dichotomised into stove use or disuse using machine learning techniques.^25^ SUM and HAP data were time matched (PM_2·5_ and CO measurements converted from 10 s to 5 min running averages). SUM data were missing for 56 (22%) of 256 study households during the HAP monitoring period, mostly due to overheating (46 [82%] of 56). The remainder of missing SUM data (ten [18%]) was due to fieldwork error that prevented linkage of the stove to the correct household. A sensitivity analysis assessed the representativeness of the 78% of households with SUM data.

### Surveys

Short surveys asking questions on cooking behaviours and time-activity patterns (eg, number of times leaving home and walking time to the nearest major road) specific to the 24 h sampling period were administered immediately before and after HAP monitoring.

### Statistical analysis

We present a descriptive analysis of multinational variations in PM_2·5_ and CO levels by cooking fuel type (LPG, wood, charcoal), cooking and non-cooking subperiods, and proxies of ambient air pollution exposure (walking time to nearest major road, whether the cook left the house during HAP monitoring). We present median real-time (5 min interval) PM_2·5_ measurements to examine fluctuations in PM_2·5_ levels within the 24 h monitoring period.

As the distribution of average 24 h PM_2·5_ and CO measurements was right skewed, data were log-transformed to meet the assumption of normality for statistical comparison. When generating SEs for 24 h mean PM_2·5_ and CO levels, mixed-effect models (assumptions described in the appendix p 10) were run to account for clustering of households within communities:

log(PM_2·5_)_ij_=β_0_ *+* β_j_ *+* β_1_(PrimaryFuelType)_i_ + *e*_ij_

where log(PM_2·5_)_ij_ is the natural logarithm of mean 24 h PM_2·5_ concentration of *i*th household in community *j*, β_0_ is the overall intercept, β_j_ is the random intercept for community *j*, and *e*_ij_ is the leftover error.

Geometric means (hereafter referred to as means) and 95% CIs were reported, with significance ascertained via non-overlapping CIs^30^ and two-sample *t* tests. No statistical corrections were applied for multiple hypothesis testing. All analyses were conducted in R (version 4.2.1) and RStudio (version 2022.7.1).^31^ The R packages dplyr,^32^ tidyverse,^33^ data.table,^34^ and ggplot2^35^ were used.

### Role of the funding source

The funder of the study had no role in study design, data collection, data analysis, data interpretation, or writing of the report.

## Results

HAP measurements were obtained in 256 kitchens (84 in Mbalmayo, 77 in Obuasi, and 95 in Eldoret), and among 248 primary cooks (80 in Mbalmayo, 75 in Obuasi, and 93 in Eldoret) and 124 children (62 in Obuasi and 62 in Eldoret). Within each study site, there were no differences in socioeconomic characteristics between the full sample of households (n=256) and the subset of households with SUM data (n=206; appendix p 12).

Heads of household were younger in households primarily using LPG (mean 39 years [SD 11]) than in those primarily using polluting fuels (45 years [12]; table 1; appendix p 13). The proportion of participants reporting financial security (“enough to meet their needs”) was twice as high among households primarily cooking with LPG (43 [34%] of 124) as among those cooking with polluting fuels (22 [16%] of 132; table 1). 244 (93%) of 262 households used electricity for lighting (table 1). 19 (8%) of 248 primary cooks reported exposure to secondhand smoke.

**Table 1:** Demographics and socioeconomic characteristics of study population by community and primary cooking fuel

		**All communities (n=262)**		**Mbalmayo, Cameroon (n=86)**		**Obuasi, Ghana (n=77)**		**Eldoret, Kenya (n=99)**	
		LPG (n=125)	Polluting (n=137)	LPG (n=43)	Polluting (n=43)	LPG (n=40)	Polluting (n=37)	LPG (n=42)	Polluting (n=57)
Mean age of primary cook, years		35 (11)	37 (12)	38 (14)	41 (13)	35 (9)	35 (11)	31 (10)	35 (11)
Mean age of child, years		3·1 (1·3)	3·1 (1·3)	NA	NA	3·3 (1·3)	3·0 (1·3)	2·8 (1·4)	3·2 (1·2)
Sex of child									
	Female	27/50 (54%)	44/74 (59%)	NA	NA	15/29 (52%)	20/33 (61%)	12/21 (57%)	24/41 (59%)
	Male	23/50 (46%)	30/74 (41%)	NA	NA	14/29 (48%)	13/33 (39%)	9/21 (43%)	17/41 (41%)
Mean age of head of household, years		39 (11)	45 (12)	41 (14)	48 (13)	35 (9)	42 (11)	39 (9)	45 (11)
Participant is head of household (female head)		22/125 (18%)	30/137 (22%)	12/43 (28%)	13/43 (30%)	6/40 (15%)	8/37 (22%)	4/42 (10%)	9/57 (16%)
Marital status									
	Married	72/125 (58%)	77/137 (56%)	17/43 (40%)	12/39 (31%)	29/40 (73%)	25/37 (68%)	26/42 (62%)	40/57 (70%)
	Single	32/125 (26%)	28/137 (20%)	10/43 (23%)	12/39 (31%)	7/40 (18%)	2/37 (5%)	15/42 (36%)	14/57 (25%)
	Cohabitating	13/125 (10%)	21/137 (15%)	11/43 (26%)	13/39 (33%)	2/40 (5%)	8/37 (22%)	0	0
	Widowed	8/125 (6%)	11/137 (8%)	5/43 (12%)	2/39 (5%)	2/40 (5%)	2/37 (5%)	1/42 (2%)	3/57 (5%)
Household size (number of members)									
	1–2	13/125 (10%)	8/137 (6%)	1/43 (2%)	0	10/40 (25%)	7/37 (19%)	2/42 (5%)	1/57 (2%)
	3–4	44/125 (35%)	32/137 (23%)	12/43 (28%)	4/43 (9%)	15/40 (38%)	12/37 (32%)	17/42 (40%)	16/57 (28%)
	5–6	45/125 (36%)	41/137 (30%)	16/43 (37%)	10/43 (23%)	14/40 (35%)	6/37 (16%)	15/42 (36%)	25/57 (44%)
	≥7	23/125 (18%)	56/137 (41%)	14/43 (33%)	29/43 (67%)	1/40 (3%)	12/37 (32%)	8/42 (19%)	15/57 (26%)
Financial security									
	Enough money to meet needs	43/125 (34%)	22/137 (16%)	10/43 (23%)	3/43 (7%)	17/40 (43%)	6/37 (16%)	16/42 (38%)	13/57 (23%)
	Not quite enough	62/125 (50%)	64/137 (47%)	25/43 (58%)	17/43 (40%)	16/40 (40%)	17/37 (46%)	21/42 (50%)	30/57 (53%)
	Definitely not enough	20/125 (16%)	51/137 (37%)	8/43 (19%)	23/43 (53%)	7/40 (17%)	14/37 (38%)	5/42 (12%)	14/57 (25%)
Highest education level									
	No formal education	5/125 (4%)	10/137 (7%)	2/43 (5%)	0	6/40 (15%)	7/37 (19%)	0	3/57 (5%)
	Primary	17/125 (14%)	41/137 (30%)	10/43 (23%)	6/43 (14%)	8/40 (20%)	7/37 (19%)	6/42 (14%)	20/57 (35%)
	Secondary or high school	71/125 (57%)	72/137 (53%)	24/43 (56%)	31/43 (72%)	21/40 (53%)	23/37 (62%)	14/42 (33%)	22/57 (39%)
	University	32/125 (26%)	14/137 (10%)	7/43 (16%)	6/43 (14%)	5/40 (13%)	0	22/42 (52%)	12/57 (21%)
Toilet in home		69/125 (55%)	27/137 (20%)	22/43 (51%)	11/43 (26%)	15/40 (38%)	6/37 (16%)	32/42 (76%)	10/57 (18%)
Primary lighting fuel									
	Electricity	124/125 (>99%)	120/137 (88%)	43/43 (100%)	41/41 (100%)	40/40 (100%)	35/37 (95%)	41/42 (98%)	42/55 (76%)
	Solar powered lantern, flashlight, or oil lamp	1/125 (1%)	17/137 (12%)	0	0	0	2/37 (5%)	1/42 (2%)	13/55 (24%)
Cooking location*									
	In home (no separate room)	14/118 (12%)	2/123 (2%)	8/43 (19%)	0	1/36 (3%)	0	5/39 (13%)	2/53 (4%)
	In home (separate room)	70/118 (59%)	14/123 (11%)	33/43 (77%)	0	6/36 (15%)	4/37 (11%)	31/39 (79%)	12/53 (23%)
	Outside home (separate room)	6/118 (5%)	61/123 (50%)	2/43 (5%)	21/34 (62%)	1/36 (3%)	4/37 (11%)	3/39 (8%)	36/53 (68%)
	Veranda or porch	25/118 (21%)	37/123 (30%)	0	13/34 (38%)	25/36 (63%)	23/37 (62%)	0	0
	Outside home (open air)	3/118 (3%)	9/123 (7%)	0	0	3/36 (8%)	6/37 (16%)	0	3/53 (6%)
Number of times leaving the home during 24 h monitoring									
	0	72/125 (58%)	61/132 (46%)	28/43 (65%)	26/42 (62%)	19/40 (47%)	12/37 (32%)	25/42 (60%)	23/53 (43%)
	1–4	51/125 (41%)	60/132 (45%)	15/43 (35%)	16/42 (38%)	19/40 (47%)	15/37 (41%)	17/42 (40%)	29/53 (55%)
	≥5	2/125 (2%)	11/132 (8%)	0	0	2/40 (5%)	10/37 (27%)	0	1/53 (2%)
Travel time to major road*									
	<5 min	49/122 (40%)	48/128 (38%)	24/43 (56%)	24/41 (59%)	10/40 (25%)	5/37 (14%)	15/39 (38%)	19/50 (38%)
	5–10 min	47/122 (39%)	44/128 (34%)	18/43 (42%)	14/41 (34%)	15/40 (38%)	18/37 (49%)	14/39 (36%)	12/50 (24%)
	>10 min	26/122 (21%)	36/128 (28%)	1/43 (2%)	3/41 (7%)	15/40 (38%)	14/37 (38%)	10/39 (26%)	19/50 (38%)
Wearing compliance† (cook)		48% (17)	51% (17)	38% (19)	39% (17)	48% (14)	53% (13)	56% (14)	58% (16)
Wearing compliance† (child) (geometric mean, GSD)‡		7% (1·5)	15% (1·2)	NA	NA	4% (1·5)	14% (0·9)	15% (1·2)	15% (1·4)

Data are n (%), n/N (%), or mean (SD). GSD=geometric SD. NA=not applicable.

* Some households were missing data so percentage does not add up to 100%.

† Estimated % of time wearing the monitor during 24 h monitoring period as determined by accelerometer data.

‡ Child wearing compliance was right skewed (appendix p 34).

70 (59%) of 118 households using LPG cooked within their main house in a separate room. However, in Obuasi, the main cooking location for LPG (25 [69%] of 36) and polluting fuels (23 [62%] of 37) was on a porch (table 1). Wood users in Eldoret and Mbalmayo predominantly cooked in separate, enclosed rooms behind their main house (table 1). Women cooking with wood in Eldoret used a mud stove (chepkube), whereas women in Mbalmayo predominantly cooked over open fires.

The proportion of participants cooking with polluting fuels who reported leaving their home during the monitoring period in Obuasi (68%) was 10 percent points (pp) higher than Eldoret (57%) and 30 pp greater than in Mbalmayo (38%; table 1). The proportion of participants living less than 10 min from a major road was 11 pp higher among households cooking primarily with LPG (79%) than in those cooking with polluting fuels (68%).

Female primary cooks wore the monitors for approximately half of the 24 h sampling period (ie, 12 h), on average (table 1). Children wore the monitors for an average of approximately 2–4 h (7–15% of monitoring period; table 1).

Across all communities, mean PM_2·5_ kitchen concentrations among all primary cooking fuel types exceeded the WHO interim-1 target level (35 μg/m^3^; table 2). However, mean 24 h PM_2·5_ kitchen concentrations in households cooking primarily with wood (341 μg/m^3^, 95% CI 229–498) or charcoal (109 μg/m^3^, 68–189) were multiple times greater than the mean concentration among households cooking with LPG (52 μg/m^3^, 39–75; table 2).

**Table 2:** PM_2·5_ and CO kitchen concentrations and personal (main cook or child) exposures stratified by primary cooking fuel type and community

	All communities (n=256)*			**Mbalmayo, Cameroon (n=84)**			**Obuasi, Ghana (n=77)**			**Eldoret, Kenya (n=95)**		
	n	PM_2·5_(μg/m^3^)	CO (ppm)	n	PM_2·5_(μg/m^3^)	CO (ppm)	n	PM_2·5_(μg/m^3^)	CO (ppm)	n	PM_2·5_(μg/m^3^)	CO (ppm)
**Kitchen concentrations**												
Number of households	256	..	..	84	..	..	77	..	..	95	..	..
LPG	124	52 (39–75)	0·65 (0·07–4·71)	43	44 (38–51)	0·99 (0·55–1·78)	40	52 (40–68)	0·31 (0·12–0·81)	41	61 (49–76)	1·06 (0·34–3·31)
Charcoal	53	109 (68–189)	8·18 (1·53–18·28)	..	NA	NA	33	56 (45–70)	1·77 (1·04–2·99)	20	297 (216–406)	15·81 (8·71–28·72)
Wood	79	341 (229–498)	14·50 (0·61–68·57)	41	287 (222–374)	5·88 (3·87–8·95)	4	314 (170–580)	NA	34	424 (322–559)	17·09 (11·13–26·26)
**Cook exposures**												
Number of cooks	248	..	..	80	..	..	75	..	..	93	..	..
LPG	119	43 (23–76)	0·57 (0·12–2·49)	42	38 (32–44)	0·23 (0·11–0·51)	38	49 (37–63)	0·25 (0·12–0·81)	39	45 (36–56)	1·31 (0·65–2·65)
Charcoal	53	78 (51–100)	2·33 (1·16–8·52)	..	NA	NA	33	60 (47–75)	1·52 (0·86–2·67)	20	110 (80–150)	3·76 (2·18–6·51)
Wood	76	101 (55–194)	2·52 (0·56–14·09)	38	75 (58–98)	0·70 (0·40–1·20)	4	114 (62–211)	NA	34	136 (104–180)	2·65 (1·74–4·02)
**Child exposures**												
Number of children	124	..	..	0	..	..	62	..	..	62	..	..
LPG	50	47 (18–128)	NA	..	NA	NA	29	41 (31–53)	NA	21	56 (38–83)	NA
Charcoal	47	59 (34–117)	NA	..	NA	NA	29	46 (32–67)	NA	18	90 (61–132)	NA
Wood	27	107 (32–275)	NA	..	NA	NA	4	61 (25–148)	NA	23	118 (82–169)	NA

Data are geometric mean (95% CI). CO=carbon monoxide. LPG=liquefied petroleum gas. NA=not applicable. PM_2·5_=fine particulate matter. ppm=parts per million.

* SEs for the all-community analysis account for clustering of households within communities.

In Eldoret, the mean PM_2·5_ kitchen concentration in households cooking primarily with charcoal was significantly higher than that in households cooking with LPG (297 μg/m^3^, 95% CI 216–406, for charcoal *vs* 61 μg/m^3^, 49–76, for LPG; p<0·0001; appendix p 19). However, mean PM_2·5_ kitchen concentrations between the two fuel types were nearly identical in Obuasi (56 μg/m^3^, 95% CI 45–70, for charcoal *vs* 52 μg/m^3^, 40–68, for LPG; p=0·71; table 2; appendix p 19). In all communities, the mean PM_2·5_ kitchen concentration in households cooking primarily with wood was more than six times greater than mean PM_2·5_ kitchen concentrations among households cooking primarily with LPG (table 2).

In Eldoret and Mbalmayo, mean 24 h PM_2·5_ kitchen concentrations among households cooking inside their house or outdoors were substantially lower than concentrations in smaller rooms separate from the main house (appendix p 27).

The mean CO kitchen concentration in households cooking primarily with wood (14·50 ppm, 95% CI 0·61–68·57) and charcoal (8·18 ppm, 1·53–18·28) exceeded the WHO air quality guideline of 6·11 ppm and were more than ten times higher than the mean CO kitchen concentration in households primarily cooking with LPG (0·65 ppm, 0·39–1·07; table 2).

The mean CO kitchen concentration in households cooking mainly with charcoal in Eldoret (15·81 ppm, 95% CI 8·71–28·72) was more than double the WHO air quality guideline, while the mean CO kitchen level among charcoal users in Obuasi (1·77 ppm, 1·04–2·99) was less than half the WHO guideline level (table 2). The mean CO kitchen level among households cooking with wood in Eldoret (17·09 ppm, 95% CI 11·13–26·26) was more than twice the WHO guideline, whereas the mean CO kitchen level among those using wood in Mbalmayo was below the guideline (5·88 ppm, 3·87–8·95).

The overall mean 24 h PM_2·5_ cook exposure among those primarily using LPG was nearly half that of charcoal primary users (43 μg/m^3^, 95% CI 23–76, *vs* 78 μg/m^3^, 51–100) and less than half that of wood primary users (101 μg/m^3^, 55–194). However, in Obuasi, primary LPG users only had 11 μg/m^3^ lower mean PM_2·5_ exposures than cooks primarily using charcoal (49 μg/m^3^, 95% CI 37–63, *vs* 60 μg/m^3^, 47–75; p=0·25). In Mbalmayo, women cooking with LPG had significantly lower exposures than primary wood users (38 μg/m^3^, 95% CI 32–44, *vs* 75 μg/m^3^, 58–98; p=0·0071; table 2). In Eldoret, the mean PM_2·5_ exposure among women cooking primarily with wood was triple that of the mean among cooks primarily using LPG (136 μg/m^3^, 95% CI 104–180, *vs* 45 μg/m^3^, 36–56).

Average CO cook exposures among those cooking primarily with charcoal (2·33 ppm, 95% CI 1·16–8·52) and wood (2·52 ppm, 0·56–14·09) were five times greater than mean levels among primary LPG users (0·57 ppm, 0·12–2·49). Average CO exposures among cooks primarily using wood in Eldoret were more than three times higher than exposures in Mbalmayo (2·65 ppm, 95% CI 1·74–4·02, *vs* 0·70 ppm, 0·40–1·20).

Among households primarily cooking with LPG, mean 24 h PM_2·5_ child exposures were either similar to their mother's exposure (Obuasi) or slightly higher (Eldoret, Mbalmayo; table 2). However, child exposures were generally lower than that of the primary cook among households cooking with polluting fuels in all communities. Average PM_2·5_ child exposures among households cooking with polluting fuels were twice as high in Eldoret as Obuasi (table 2).

Intraclass correlation coefficients from mixed-effects models of 24 h mean PM_2·5_ and CO kitchen concentrations and personal exposures across all settings were low (≤0·13; appendix p 17), indicating high within-community variance in HAP levels.

Households cooking exclusively with LPG used their stove for approximately half as long as those exclusively using polluting fuels (1·75 h *vs* 3·40 h; appendix p 14). Households using LPG as a primary fuel and polluting fuels as a secondary fuel used their stoves for more than three times longer than exclusive LPG users during HAP monitoring (5·49 h *vs* 1·75 h).

**Figure 1:**
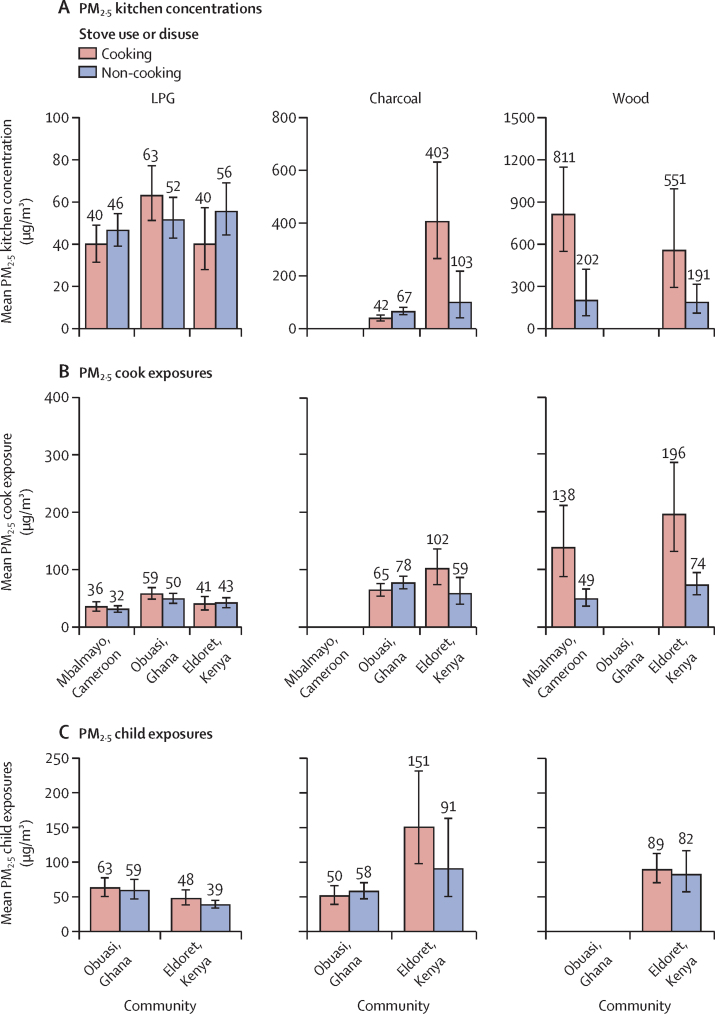
Geometric mean PM_2·5_ kitchen concentrations (A) and cook (B), and child (C) exposures during cooking and non-cooking periods stratified by community and primary fuel type Error bars represent 95% CIs. LPG=liquefied petroleum gas. PM_2·5_=fine particulate matter.

In Mbalmayo and Eldoret, mean PM_2·5_ kitchen concentrations in households primarily using LPG were approximately 15 μg/m^3^ higher during non-cooking periods than cooking periods (46 μg/m^3^, 95% CI 39−54, *vs* 40 μg/m^3^, 32−49, and 56 μg/m^3^, 45−70, *vs* 40 μg/m^3^, 28−58, respectively; figure 1A). By contrast, mean PM_2·5_ kitchen concentrations during cooking periods were significantly higher than levels during non-cooking periods among households primarily cooking with wood in Mbalmayo (p<0·0001) and Eldoret (p=0·043; appendix p 21). The mean PM_2·5_ kitchen concentration among households cooking with wood in Mbalmayo was four times higher during cooking periods than during non-cooking periods (811 μg/m^3^, 95% CI 569−1157, *vs* 202 μg/m^3^, 155−264; figure 1A). However, among households cooking primarily with charcoal in Obuasi, the mean PM_2·5_ kitchen concentration was 25 μg/m^3^ lower during cooking periods than during non-cooking periods (42 μg/m^3^, 95% CI 32−55, *vs* 67 μg/m^3^, 58−77).

Average PM_2·5_ cook exposures were significantly higher during cooking periods than during non-cooking periods across all primary cooking fuel types in Mbalmayo (p<0·0001) and Eldoret (p=0·002–0·04; appendix p 21). However, this difference was not significant among LPG (p=0·51) and charcoal users (p=0·37) in Obuasi (appendix p 21). Average PM_2·5_ child exposures during cooking periods were not significantly higher than exposures during non-cooking periods among any cooking fuel types in Obuasi or Eldoret (p=0·47–0·85; appendix p 21).

Mean CO cook exposures were not significantly higher during cooking periods than non-cooking periods across all cooking fuel types and communities (appendix p 20). Among households cooking primarily with wood, the mean CO kitchen concentration during cooking periods exceeded the WHO guideline (6·11 ppm) in all communities (7·39 ppm, 95% CI 0·82–23·19, in Obuasi; 14·74 ppm, 1·14–24·98, in Mbalmayo; 30·15 ppm, 1·45–54·00, in Eldoret; appendix p 20). Among households cooking primarily with charcoal, the mean CO level during cooking period exceeded the WHO guideline in Eldoret (21·52 ppm, 95% CI 1·24–35·00) but not in Obuasi (5·43 ppm, 1·44–10·06). Average CO kitchen concentrations and cook exposures during cooking periods were below the WHO guideline among households primarily cooking with LPG. Mean CO cook exposures exceeded the WHO guideline during cooking periods among households primarily cooking with wood and charcoal in all communities (appendix p 20).

**Figure 2:**
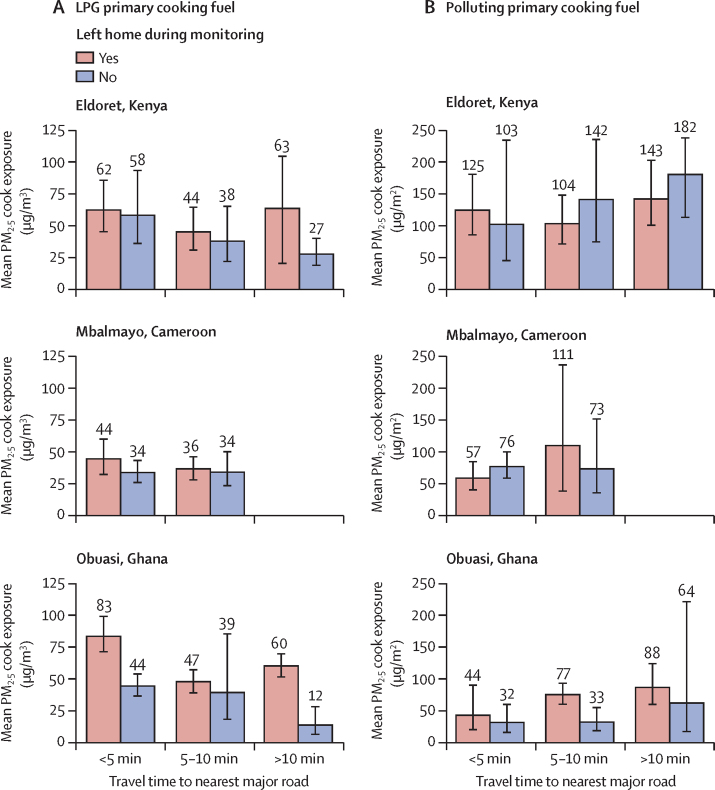
Geometric mean 24 h PM_2·5_ cook exposure among households primarily cooking with LPG (A) and households primarily cooking with polluting fuels (B) by travel distance to nearest major road and whether they left their home during the 24 h monitoring Error bars represent 95% CIs. LPG=liquefied petroleum gas. PM_2·5_=fine particulate matter.

In all communities, cooks primarily cooking with LPG who travelled outside their household had higher PM_2·5_ exposures than cooks who remained indoors (figure 2A). In Mbalmayo, LPG users living less than 5 min from the main road and leaving their household during the HAP monitoring had 10 μg/m^3^ higher mean PM_2·5_ exposures than LPG users living within 5 min of a main road but remaining in their home (46 μg/m^3^, 95% CI 41–52, *vs* 35 μg/m^3^, 32–38).

In Obuasi and Eldoret, mean 24 h PM_2·5_ cook exposures only met the WHO interim-1 target of 35 μg/m^3^ among primary cooks using LPG who lived more than 10 min away from a major road and never left their home (figure 2A).

Mean PM_2·5_ cook exposures among LPG users not leaving their home during the 24 h monitoring monotonically declined (p_trend_=0·10) with increasing distance to the main road in Obuasi and Eldoret (figure 2A). Among LPG users in Obuasi who remained indoors, the average PM_2·5_ exposure among cooks who lived less than 5 min from a main road was nearly four times that of cooks living more than 10 min from a main road who stayed inside (44 μg/m^3^, 95% CI 33–57, *vs* 12 μg/m^3^, 6–28; figure 2A). In Eldoret, the mean PM_2·5_ exposure among cooks using LPG who remained indoors and lived less than 5 min from a main road was double that of cooks using LPG and living more than 10 min from a main road that stayed indoors (58 μg/m^3^, 95% CI 36–93, *vs* 27 μg/m^3^, 19–40).

A monotonically increasing relationship (p_trend_=0·021) existed between times leaving the household and mean PM_2·5_ cook-to-kitchen ratio in Obuasi; the same relationship was not present in other communities or among CO cook-to-kitchen ratios (appendix p 25). In Obuasi, the median PM_2·5_ cook exposure was higher than the median PM_2·5_ kitchen concentration among those travelling outside more than four times (appendix p 25). Mean CO cook-to-kitchen exposure ratios (appendix p 25) did not differ on the basis of whether the cook left the household.

**Figure 3:**
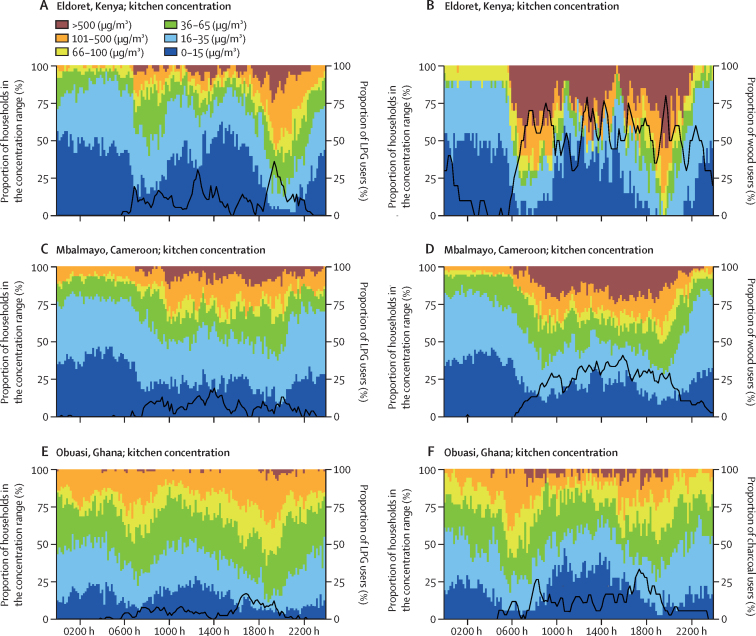
Real-time PM_2·5_ kitchen concentration measurements among households primarily cooking with LPG (left) and polluting fuels (right) in Mbalmayo (Cameroon), Obuasi (Ghana), and Eldoret (Kenya) The y axis on the left shows the proportion of households in each PM_2·5_ concentration range. The y axis on the right shows the proportion of households using their stove at any given time of day. LPG=liquefied petroleum gas. PM_2·5_=fine particulate matter.

Spikes in real-time PM_2·5_ kitchen concentrations (figure 3) and cook exposures (appendix pp 29–32) during typical breakfast hours (eg, 0600−0900 h) and dinner hours (eg, 1700–2000 h) were substantially larger in Eldoret than in Mbalmayo and Obuasi (reflected by the red bars). The proportion of households using their stove during any given hour among exclusive users of polluting fuels was also substantially higher in Eldoret than in Mbalmayo and Obuasi (figure 3). PM_2·5_ kitchen concentrations exceeded 500 μg/m^3^ during dinner time (1700–2000 h) in approximately 25% of households cooking with wood in Mbalmayo and Eldoret (figure 3) and charcoal in Eldoret (appendix p 27), compared with only around 10% of households cooking with charcoal (figure 3) and wood (appendix p 27) in Obuasi.

During the middle of the night (0100–0400 h), PM_2·5_ kitchen concentrations (figure 3) and cook exposures (appendix p 29–32) were noticeably higher in Obuasi than in Mbalmayo and Eldoret; from 0100 h to 0400 h, PM_2·5_ kitchen concentrations remained above the WHO interim-1 target (35 μg/m^3^) among approximately 50% of LPG-using households in Obuasi (figure 3E) compared with 15–25% of LPG-using households in Eldoret (figure 3A) and Mbalmayo (figure 3C).

Spikes in CO kitchen concentrations and cook exposures at breakfast and dinner time were similarly higher in Eldoret than in Mbalmayo and Obuasi (appendix pp 33–37). When cooking was taking place, the WHO guideline for CO of 6·11 ppm was exceeded in approximately 10–30% of kitchens in Mbalmayo (appendix p 33) and Obuasi (appendix p 34) compared with around 30–80% of kitchens in Eldoret (appendix p 35). Real-time CO cook exposures were slightly higher in households primarily cooking with charcoal than in those cooking with wood in Eldoret and Obuasi (appendix pp 33–37).

**Figure 4:**
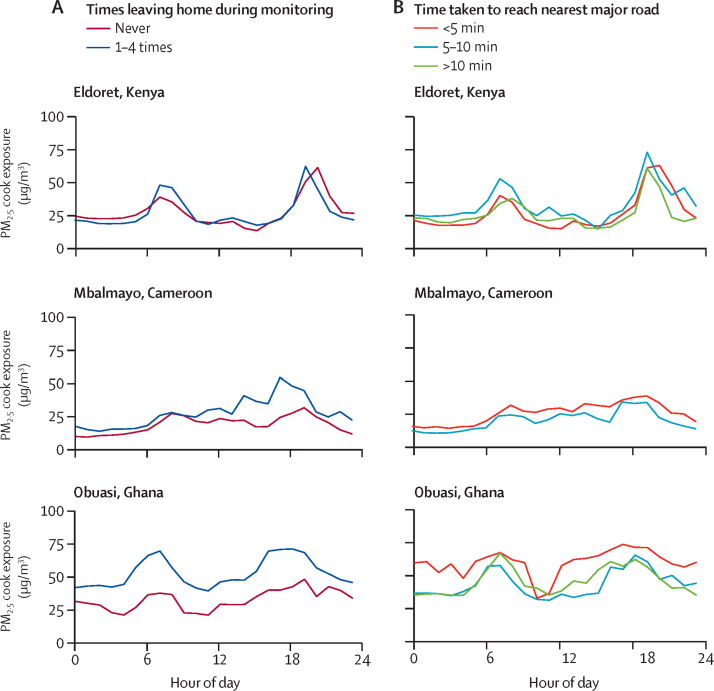
Average real-time PM_2·5_ cook exposures stratified by whether the participant left their household during the 24 h monitoring period (A) and travel time to the nearest major road (B) PM_2·5_=fine particulate matter.

Cooks who reported leaving their household during the 24 h monitoring period in Obuasi and Mbalmayo had consistently higher median PM_2·5_ exposures at every hour of the day than cooks who never left the household (~15–30 μg/m^3^ for Obuasi and ~5–20 μg/m^3^ for Mbalmayo; figure 4). In Eldoret, no differences in real-time PM_2·5_ cook exposures emerged according to whether they left their household.

Cooks living closest (<5 min) to major roads had higher median PM_2·5_ exposures than those living more than 5 min from a road at almost all hours of the day in Mbalmayo and Obuasi (figure 4). In Obuasi, median real-time PM_2·5_ exposures among cooks living less than 5 min from a main road exceeded 50 μg/m^3^ throughout the day. By contrast, median PM_2·5_ exposures among cooks living more than 5 min from a major road in Obuasi only exceeded 50 μg/m^3^ during breakfast (eg, 0600–0900 h) and dinner (eg, 1700–2000 h; figure 4). In Eldoret, no patterns in real-time PM_2·5_ cook exposures existed based on travel time to the nearest major road.

## Discussion

This multisite HAP measurement study uncovered large community-level variations in the relative difference in mean PM_2·5_ and CO kitchen concentrations and cook and child exposures among peri-urban households cooking with LPG and polluting fuels in sub-Saharan Africa.

Higher median PM_2·5_ exposures among cooks in Obuasi living less than a 5 min walk from a main road than among those living farther away might explain the minimal PM_2·5_ exposure differences between fuel groups in that community (a greater proportion of LPG users lived proximal to a main road compared with polluting fuel users; table 1). A higher proportion of primary cooks in Obuasi leaving their home during the 24 h monitoring period than those in Mbalmayo, regardless of their occupation (appendix p 41), might also explain the apparent stronger influence of ambient pollution sources on PM_2·5_ cook exposures in Obuasi.

The minor difference in average PM_2·5_ concentrations in Obuasi could also be attributed to a high prevalence of outdoor (veranda) cooking. As ambient PM_2·5_ concentrations were higher in Obuasi (mean 31 μg/m^3^) than in Mbalmayo (14 μg/m^3^) and Eldoret (6 μg/m^3^; appendix p 37), it is likely that infiltration of ambient PM_2·5_ into kitchens increased concentrations among households cooking with LPG.^36^ Another study conducted in urban Ghana found that road dust and vehicle emissions constituted 12−33% of kitchen PM_2·5_ mass.^37^ The authors reported a high air exchange rate between household and ambient environments in Ghana since homes typically have cracks in walls or windows, and doors are often kept open. The strong influence of ambient air pollution in Obuasi is also evidenced by the monotonically increasing relationship between (1) shorter travel time to the main road and mean PM_2·5_ cook exposure and (2) number of times leaving the household and a higher PM_2·5_ cook-to-kitchen exposure ratio (appendix p 25).

As CO levels are generally a better marker of combustion sources than PM_2·5_ concentrations, the potentially lower contribution of HAP to overall kitchen concentrations in Obuasi compared with the other two communities is further evidenced by a more than 50% lower Spearman correlation between mean cooking time PM_2·5_ and CO kitchen concentrations in Obuasi (*r*_S_=0·27) compared with Eldoret (*r*_S_=0·48) and Mbalmayo (*r*_S_=0·67; appendix p 22); the same pattern across communities was also found for mean non-cooking kitchen concentrations. A systematic review of HAP studies assessing the association between PM_2·5_ and CO levels reported a stronger correlation between measurements collected in rural (*R*^2^=0·42) versus peri-urban settings (*R*^2^=0·25), and stated that the difference might be attributed greater pollution from community-level and regional sources in peri-urban neighbourhoods relative to rural settings.^38^ HAP measurements in Obuasi occurring in the Harmattan season (November–March), when trade winds blow Sahara Desert dust across west Africa,^39^ might explain why ambient PM_2·5_ concentrations measured in Obuasi were higher than in the other two communities.^40^

A study conducted in west Africa found a rural-to-urban gradient in the relative contribution of polluting fuel use to PM_2·5_ levels: biomass burning accounted for 74−87% of PM_2·5_ kitchen concentrations in rural Gambia, but only 39−62% of concentrations in urban Ghana.^37^ Clean cooking interventions have also had mixed success in achieving HAP exposure reductions based on urbanicity:^23^ an intervention in rural Rwanda resulted in substantial PM_2·5_ exposure reductions (109 μg/m^3^ *vs* 43 μg/m^3^),^41^ while no reduction was found in a trial conducted in urban Nigeria (42 μg/m^3^ *vs* 42 μg/m^3^).^17^ The lack of PM_2·5_ exposure reductions in the Nigerian trial might be due to a high household density that led to HAP from nearby homes affecting neighbours’ exposures.^17^ In this study, we found that ambient air pollution might also curtail PM_2·5_ exposure reductions when using clean cooking fuels in peri-urban communities in sub-Saharan Africa, and that the influence of ambient air pollution on overall exposures can vary substantially across peri-urban settings.

Cooks in Eldoret who used polluting fuels in their main house had approximately 90% lower PM_2·5_ kitchen concentrations and 50% lower PM_2·5_ exposures than those cooking in a small, enclosed room behind their home (appendix p 27). This suggests that ventilation also influenced PM_2·5_ levels.^36^

Lower average PM_2·5_ exposures among cooks primarily cooking with LPG than among those using polluting cooking fuels in Eldoret and Mbalmayo indicates that a population-level transition might result in improved health due to the exposure−response relationship between PM_2·5_ exposures and cardiovascular and respiratory conditions.^12^ Conversely, the minimal difference in PM_2·5_ levels between cooks primarily using LPG and those using charcoal in Obuasi along with the higher PM_2·5_ kitchen concentrations during non-cooking periods than during cooking periods in Eldoret and Mbalmayo indicate that minimal health gains might be realised when substituting charcoal with LPG for cooking in that community.

Our study reveals that mean 24 h PM_2·5_ cook exposures only met the WHO interim-1 among cooks using LPG who lived more than 10 min walk from a major road and stayed inside their household. Another multinational HAP measurement study also reported a mean PM_2·5_ exposure of LPG users (45 μg/m^3^) above the WHO interim-1 and concluded that time spent outdoors affected PM_2·5_ exposures.^16^

Thus, an emphasis on transitioning to clean cooking fuels in areas where ambient PM_2·5_ pollution is lower in the short term might help to more efficiently allocate limited resources to have a larger health benefit. Ultimately, policies are probably needed to promote access to cleaner cooking fuels alongside programmes addressing ambient air pollution (eg, crop burning, trash burning, traffic)^15^, ^16^, ^17^ to help ensure that PM_2·5_ levels meet the WHO interim-1 target^18^ in rapidly urbanising sub-Saharan Africa. However, there is not a threshold for PM_2·5_ concentrations by which no adverse health effects are expected.^42^

Significantly higher mean CO kitchen levels were seen during cooking periods than those during non-cooking periods among households primarily cooking with charcoal in Obuasi and wood in Eldoret and Mbalmayo, which suggests that emissions from polluting fuels were a main contributor to indoor CO levels. A four times higher mean 24 h CO exposure among cooks primarily using wood in Eldoret than in Mbalmayo (table 2) might be largely due to ventilation; Kenyan women cooked with wood stoves indoors, whereas half of the women in Cameroon cooked outdoors on a porch.

Minimal differences in mean PM_2·5_ cook and child exposures during cooking periods in Obuasi deviates with the scientific literature, which postulates that women are consistently exposed to the highest PM_2·5_ levels in their family because of their typical role as the primary cook. Additionally, higher mean 24 h PM_2·5_ child exposures relative to that of the primary cook in LPG-using households in Eldoret were driven by children's exposure to higher PM_2·5_ levels during non-cooking periods relative to their mothers. A rural Ghanaian study reported that ambient PM_2·5_ levels were a better predictor of children's PM_2·5_ exposures than of their mother's exposure, and that a greater time spent playing outdoors might increase children's PM_2·5_ exposures.^43^

The integrated PM_2·5_, CO, and SUM measurements allowed for an objective comparison of HAP exposures under real-world scenarios, which is an advantage over intervention studies. However, seasonal measurements were not collected in study communities to measure potential changes in HAP kitchen concentrations and personal exposures due to differences in fuels used and other cooking patterns. Thus, our 24 h measurements might not reflect average annual HAP concentrations; future measurement studies conducted in sub-Saharan Africa should assess seasonal variability in HAP levels, especially as Sahara dust contributes to seasonal fluctuations in PM_2·5_ concentrations.^40^

There could be self-report bias introduced by participants providing their walking time to nearest major road; however, the rounding of travel time to 5 min intervals might have reduced reporting errors. We collected ambient measurements that depicted substantially higher ambient PM_2·5_ levels in Obuasi than in the other two communities (appendix p 38). However, more spatially resolved ambient air pollution measurements are needed in future studies to confirm the substantial contribution of ambient to HAP levels, as evidenced by the proxy variables.^44^

Although SUM data covering the HAP monitoring period were missing for 20% of study households, a sensitivity analysis revealed that the sociodemographic profile of the subset of households with SUM data did not differ from that of the full sample (appendix p 12), suggesting that the data were missing at random and were likely representative of cooking patterns within the study population.

Although our study collected extensive survey data on environmental factors that can affect HAP exposures and used objective measures obtained from stove use monitoring, other potential determinants were not evaluated. For example, we did not collect information on trash burning or cooking fuels used by participants’ neighbours. These sources can affect PM_2·5_ exposures, particularly if a participant spends substantial time in the immediate outdoor area surrounding their household.^45^ Moreover exposure misclassification of HAP kitchen concentrations might exist among households stacking fuels if the cooking location between primary and secondary fuels differed. For this reason, our analysis focused on characterising HAP levels by primary cooking fuel type (by which the monitors were placed).

As primary cooks in our study self-reported the number of times they travelled out of their households, it could be possible that some who reported leaving their home remained in the immediate vicinity;^45^ thus, their exposure to air pollution sources might not have been very different from that inside their home. Additionally, the absence of geolocated data precluded an assessment of the impact of community-level use of polluting cooking fuels on participants’ air pollution exposure levels. Future studies should combine HAP measurements with GPS or Bluetooth technology^46^ to estimate exposure levels in different microenvironments and better isolate the impact of ambient PM_2·5_ on overall exposure.

Although quantifying HAP variations during cooking and non-cooking periods illustrated the contribution of cooking emissions to overall exposures, the HAP exposure patterns in our study might not carry over to rural or urban settings because of differences in levels of ambient air pollution and LPG penetration, which can affect rates of stove stacking.

This study is one of few^43^, ^47^, ^48^ providing direct measurements of children's HAP exposures in sub-Saharan Africa. We collected measurements of PM_2·5_ child exposure in 74% of households that received PM_2·5_ cook exposure monitoring, a sensitivity analysis revealed no significant differences in socioeconomic characteristics between the full sample and the reduced sample of households where children were monitored (appendix p 40), suggesting that the data were missing at random. As ECM wearing compliance among children was poor (15% of monitoring period; table 1), child exposures should be interpreted with caution. Other studies have recommended using a microenvironmental method to estimate children's exposure due to low compliance.^48^

A diverse air pollution exposure landscape was found across peri-urban sub-Saharan Africa. An evident PM_2·5_ exposure gradient across proxies of ambient PM_2·5_ exposure suggests that background ambient air pollution is possibly driving the variation in overall PM_2·5_ cook exposures between communities. These results signal that transitioning to clean cooking fuels might lead to varied HAP exposure reductions, and possibly different health impacts, across peri-urban communities of sub-Saharan Africa.

### Data sharing

The air pollution monitoring data presented in this study is under use by CLEAN-Air(Africa) for other research but can be made available to researchers upon reasonable request directed to the corresponding author.

## Declaration of interests

We declare no competing interests.
