# Supplementary material for: Household concentrations and female and child exposures to air pollution in peri-urban sub-Saharan Africa: measurements from the CLEAN-Air(Africa) study
Source: Lancet Planet Health. 2024 Feb 6;8(2):e95–e107. doi: 10.1016/S2542-5196(23)00272-3 (PMC10864747; doi:10.1016/S2542-5196(23)00272-3)
Supplement: Supplementary appendix [file mmc1.pdf]

THE LANCET
Planetary Health

Supplementary appendix

This appendix formed part of the original submission and has been peer reviewed. We post it as supplied by the authors.

Supplement to: Shupler M, Tawiah T, Nix E, et al. Household concentrations and female and child exposures to air pollution in peri-urban sub-Saharan Africa: measurements from the CLEAN-Air(Africa) study. *Lancet Planet Health* 2024; **8:** e95–107.

Household concentrations and female and child exposures to air pollution in peri-urban sub-Saharan Africa: measurements from the CLEAN-Air(Africa) study

Matthew Shupler[1*], Theresa Tawiah[2], Emily Nix[1], Miranda Baame[3], Federico Lorenzetti[1], Emmanuel Betang[3], Ryan Chartier[4], Judith Mangeni[5], Adithi Upadhya[1], Rachel Anderson de Cuevas[1], Edna Sang[5], Ricardo Piedrahita[6], Michael Johnson[6], Daniel Wilson[7], Seeba Amenga-Etego[2], Mieks Twumasi[2], Sara Ronzi[1], Diana Menya[5], Elisa Puzzolo[1], Reginald Quansah[8], Kwaku Poku Asante[2], Daniel Pope[1], Bertrand Hugo Mbatchou Ngahane[3]

1. Department of Public Health, Policy and Systems, University of Liverpool, United Kingdom
2. Kintampo Health Research Centre, Kintampo, Ghana
3. Douala General Hospital, Douala, Cameroon
4. RTI International, Research Triangle Park, North Carolina, USA
5. School of Public Health, Moi University, Eldoret, Kenya
6. Berkeley Air Monitoring Group, Berkeley, California, USA
7. Geocene Inc., Berkeley, California, USA
8. School of Public Health, University of Ghana, Ghana

* Corresponding author: Matthew Shupler; m.shupler@liverpool.ac.uk

Contents

Monitor placement ... 4

Sample size calculation ... 5

Filter analysis ... 8

MicroPEM and ECM calibration and quality control procedures ... 9

Data cleaning of real-time PM2.5 measurements ... 9

Assumptions for mixed effect models ... 10

Socioeconomic characteristic comparison for sub-sample with stove use monitoring ... 12

Cooking times during 24-hour monitoring period ... 13

Integrating household air pollution and stove use measurements ... 14

Exceedance of WHO thresholds ... 23

Cook:kitchen ratio ... 25

Travel time to nearest main road ... 25

Fuel stacking ... 26

Real-time PM2.5 kitchen concentration measurements ... 29

Real-time PM2.5 cook exposure measurements ... 29

Real-time carbon monoxide kitchen concentration measurements ... 32

Real-time carbon monoxide cook exposure measurements ... 35

Ambient air pollution levels ... 37

Cooking hours ... 38

Wearing compliance ... 39

Sensitivity analysis: Socioeconomic characteristic comparison for sub-sample with child monitoring ... 40

Characteristics by occupation ... 41

Research protocol ... 43

The CLEAN-Air (Africa) Global Health Research Group sponsored by the UK National Institute of Health Research ... 44

Liquified Petroleum Gas (LPG) to address the burden of disease from household air pollution in Sub-Saharan Africa ... 44

Choice of focus countries for CLEAN-Air (Africa) ... 45

Over-arching research goal ... 46

Goal: ... 46

Aims and objectives ... 47

Methods ... 48

Study setting 48
Community survey (all countries) 48
Household air pollution measurement and stove use monitoring (all countries) 49
Data Management and Analysis 51
Quantitative analysis of cross-sectional data: 51
Quantitative methods – Household Air Pollution measurements: 51
Quantitative methods – Stove Use Monitors (SUMS): 52
Quantitative methods – Sample Size Calculations: 52
Sample size requirements 52
Summary of sample size requirements 53
References for sample size calculations: 54
Ethics approvals 54
Gaining informed consent and withdrawal 54
Data anonymization, processing, and storage 55
References 56

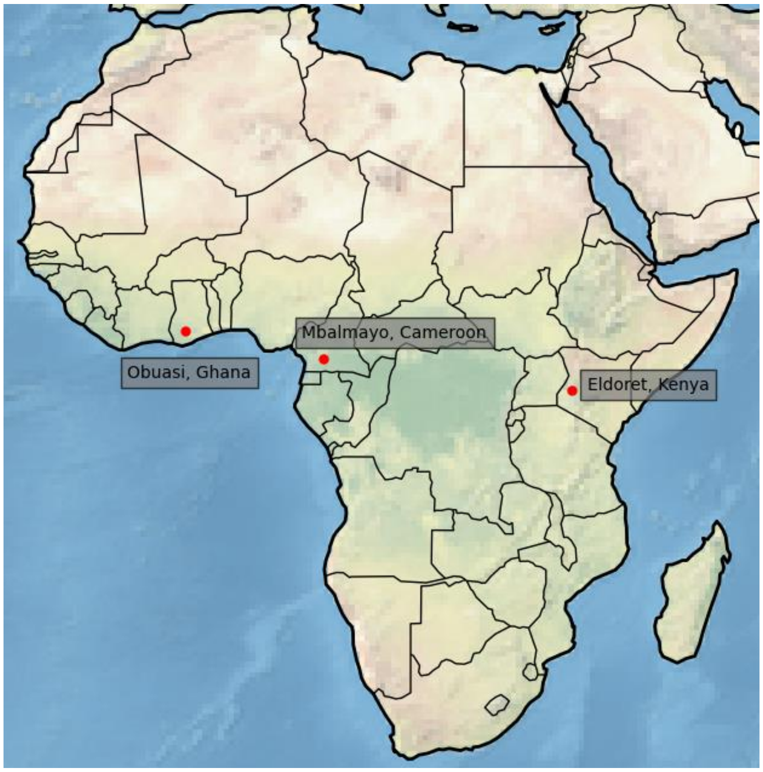

Supplementary Figure 1. Map of three study communities

Monitor placement

Field team members were recruited from universities/research centers within each country (see Ethical Approval section for list of institutions) to conduct the HAP monitoring, and all sampling equipment was procured by the University of Liverpool. Prior to data collection, the corresponding author and other researchers from the University of Liverpool traveled to the three communities to transport the equipment, train the field team on their use and collect pilot/calibration data. One to two days prior to the sampling date, field team members called participants that were randomly selected to receive HAP monitoring (using phone numbers obtained from phase 1 and phase 2 surveys) to determine their availability. The field team would arrange a household visit at a convenient time.

In each community, kitchen monitoring was conducted by placing the microPEM and Lascar monitors on a stand 1.5 meters above ground and 1 meter away from the primary stove in a location that would not constrain cooking activities. Exposures were monitored by wearing the monitors in the chest area, close to the breathing zone. Participants wore the monitor in a locally designed apron or sling (cook; at their discretion) (Supplementary Figure 1) or apron (child) If a small child was unable to wear the ECM for part of the sampling (commonly due to fear of the noise generated by the pump or heaviness of the monitor), their mother was instructed to place the monitor on a table near the child.

The same set of HAP monitors (19 MicroPEMs and 11 ECMs) were used in all three communities; monitors were shipped to the next site once sampling concluded in one community. Monitoring began in Mbalmayo, Cameroon (June-August 2019), continued in Eldoret, Kenya (September 2019-January 2020) and concluded in Obuasi, Ghana (February-March 2020). Between monitoring campaigns, MicroPEM/ECM data was downloaded to a laptop computer and transferred to cloud storage. Filters were extracted from the MicroPEM/ECM and placed in plastic filter keepers and filter cassettes were cleaned as needed. Rechargeable batteries were charged overnight before each deployment of the MicroPEM.

Prior to each sample, new filters were installed in the MicroPEM/ECM and monitors were calibrated with a flow meter to ensure a flow rate of 0.4 and 0.3 (+/- 0.15) L/min, respectively. The pumps within the MicroPEM/ECM were also checked before and after each deployment to ensure the devices maintained the proper flow rate. Pumps were replaced as needed when they became too noisy or did not maintain flow. Any between-monitor variability in measurements, which may be +/- 20% due to variability in laser output power and detector response, were minimized with filter correction. We monitored baseline detector voltage to identify monitors that may be getting dirty and contaminate the sample. When this occurred, the beam trap glass was cleaned to revert the baseline voltage back into normal range.

Air pollution monitoring data wasere stored in csvcomma-separated value (CSV) files uploaded to Dropbox weekly by study coordinators in each respectively country. Files were reviewed weekly by RTI to check for errors in data (e.g. sampling period too short, flow rate not within acceptable range) that would trigger the need for a re-sample in the household (see Appendix for more details). Stove monitoring data was transferred to the Geocene platform via a wireless internet connectionwifi. Once the temperature data was converted to 'stove use/disuse' using machine learning (ML) algorithms, the data wasfiles were downloaded into csv files. Themerged with HAP and SUMs csv files from each country were collated and merged with survey data by household ID using R. Any potential issues with mislabeling of household identifiers were reconciled with the field coordinators.

Sample size calculation

The sample size for HAP monitoring was calculated based on a modelling paper of $PM_{2.5}$ exposure reductions from previous LPG cookstove interventions.[29] The study reported a mean female exposure in polluting fuel-using homes of 270 $\mu g/m^3$ (SD = 250 $\mu g/m^3$) and a mean female exposure of 70 $\mu g/m^3$ (SD = 50 $\mu g/m^3$) in LPG-using homes. Targeting a 5% level of significance (alpha = 0.05) and 80% power yielded a sample size of 26 female cooks per fuel group (clean and polluting). Adding a 50% buffer to account for issues with monitoring equipment or sample contamination led to a target sample of 39 cooks per fuel group. This was rounded up to 40 households primarily cooking with LPG and 40 exclusively cooking with polluting fuels in each community.

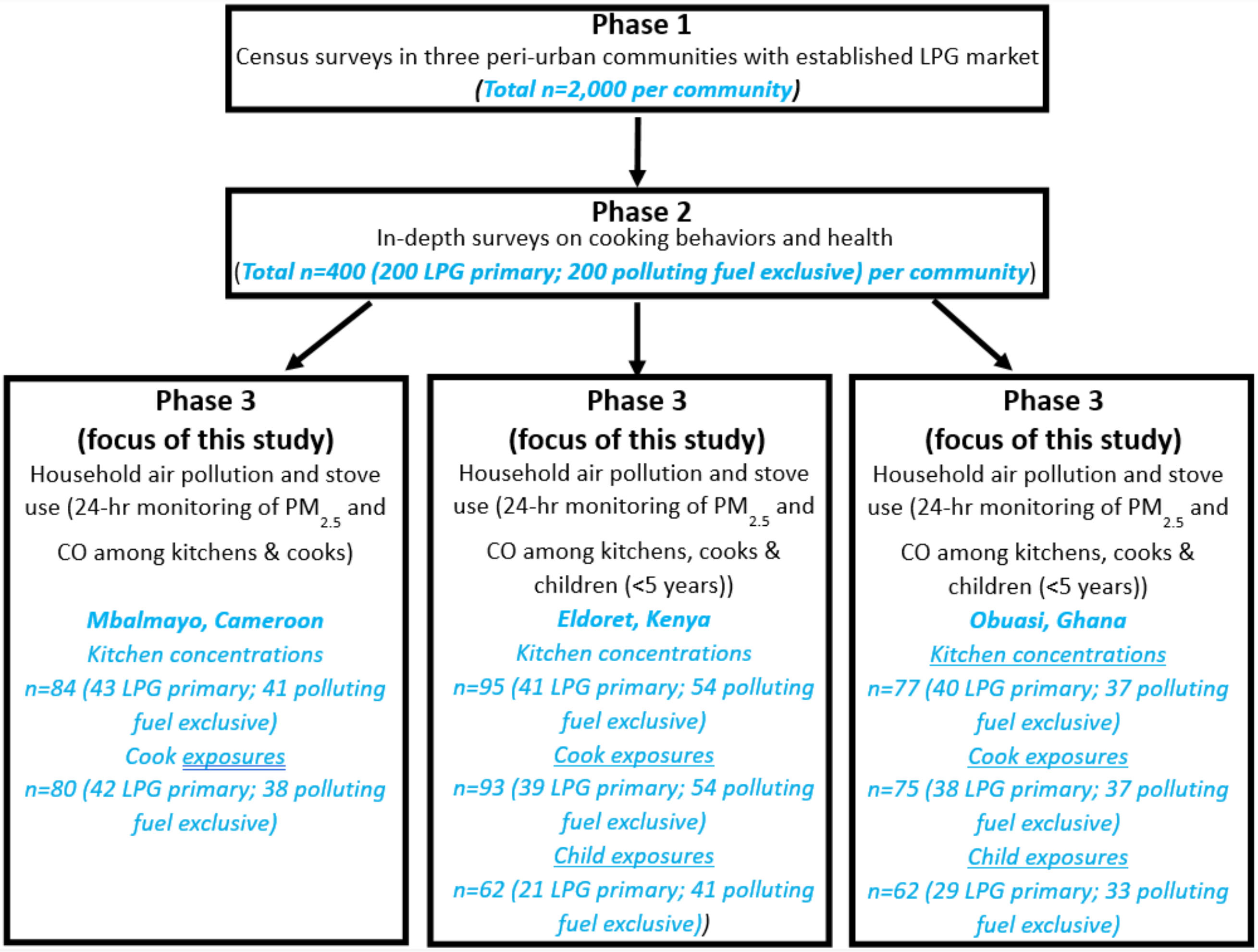

Supplementary Figure 2. Flow diagram of the different phases of the CLEAN-Air(Africa) Group study

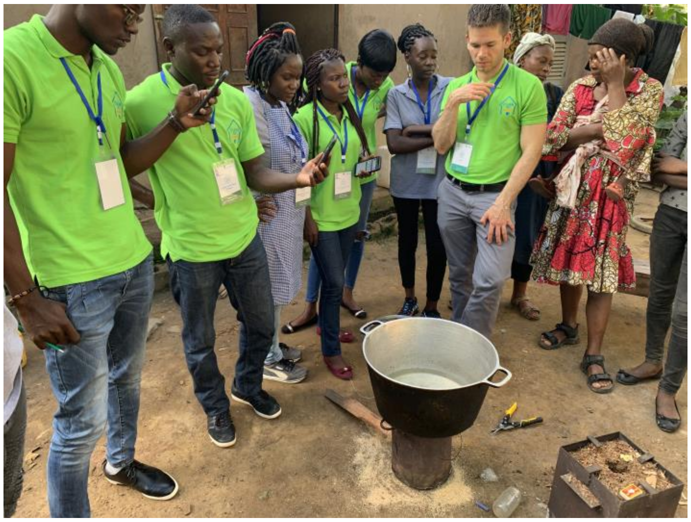
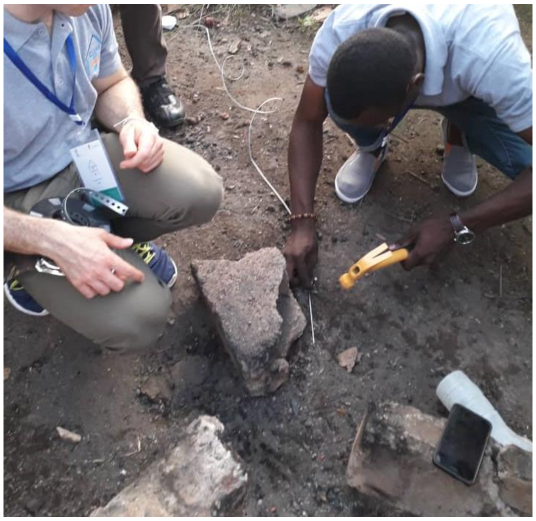
Supplementary Figure 3. HAP and stove use monitoring training conducted with field team members prior to data collection phase

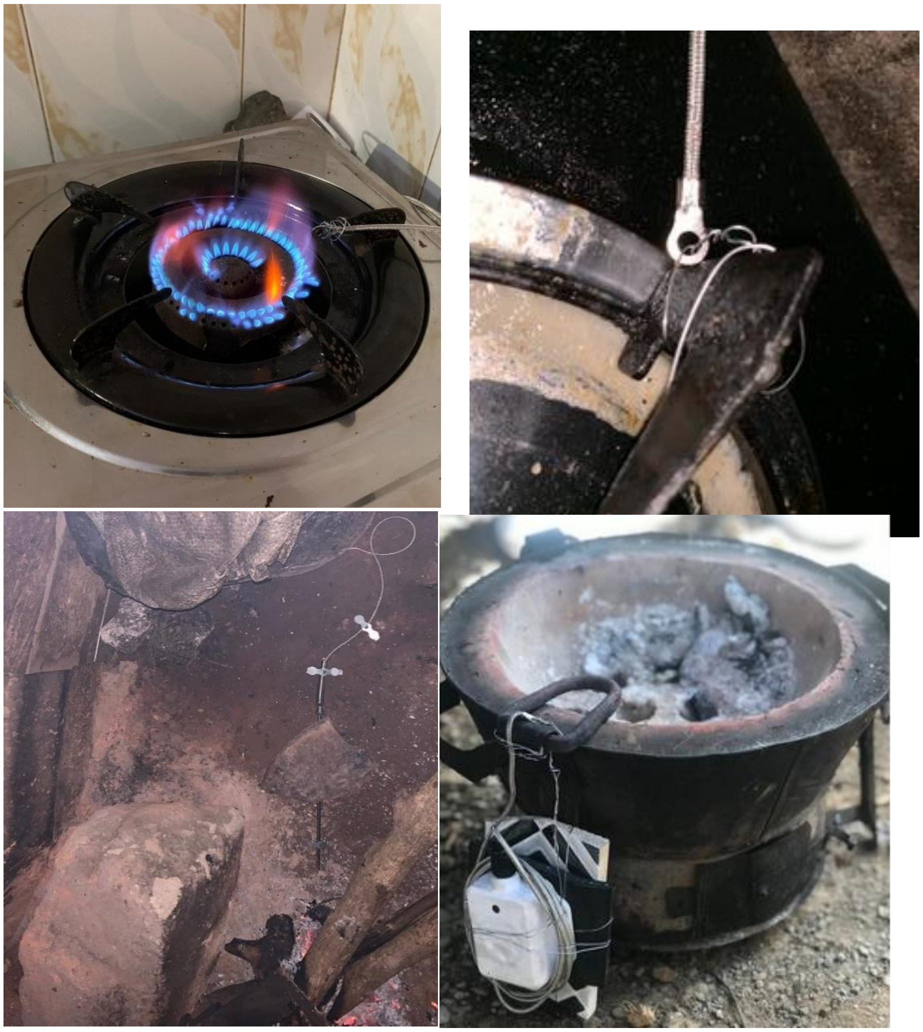

Supplementary Figure 4. Stove use monitors (Geocene dots) placed on an LPG stove (top), three stone fire (bottom left) and charcoal stove (bottom right)

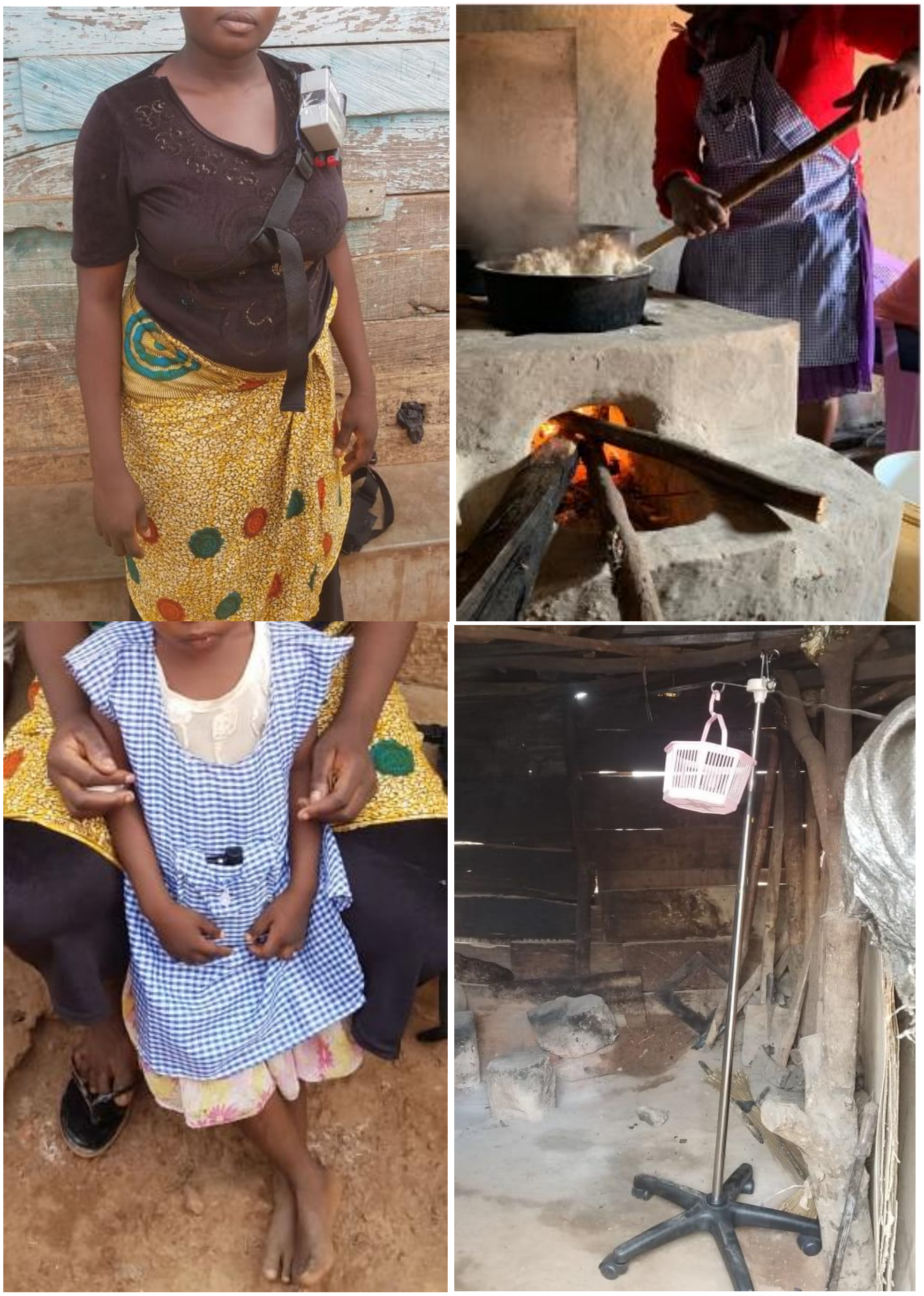

Supplementary Figure 5. Pictures of HAP monitoring set up. Primary cook wearing monitors in a sling (top left) and apron (top right). Child wearing monitor in an apron (bottom left). Monitors placed 1 meter away from the primary stove on a stand (bottom right).

Filter analysis

Gravimetric measurements were obtained by pre- and post-weighing PTFE filters using an electronic microbalance (Mettler Toledo, USA) in a temperature and humidity-controlled chamber. The filter's limit

of detection (4 µg/m^3) was calculated by taking three times the standard deviation of $PM_{2.5}$ concentrations on blank filters.

Any errors detected from the real-time $PM_{2.5}$ data triggered a re-sampling of HAP measurements to maximize the sample size in each community. If a gravimetric sample was deemed invalid due to a damaged filter or faulty flow rate, nephelometer data was used to estimate $PM_{2.5}$ concentrations normalized to per-device field-based filters.[1] We excluded $PM_{2.5}$ samples for which the monitor operated for less than 85% (20 hours) of the 24-hour measurement period.

MicroPEM and ECM calibration and quality control procedures

We did not conduct a between-device comparison as the timing and scheduling of the measurements in the three countries was not conducive to doing an intercomparison study. However, previous data from collocated MicroPEMs in sub-Saharan Africa has shown high correlation between monitors.[2,3]

Any between-device differences in measurements, which may be +/- 20% due to variability in laser output power and detector response, are minimized after filter correction. This ensures that the mean real-time concentration is equal to the integrated filter concentration.

We monitored baseline detector voltage to identify units that may be getting dirty and risk contaminating the sample. When this occurred, the beam trap glass was cleaned to revert the baseline voltage back into normal range. The pumps within the MicroPEM/ECM were checked before and after each deployment to ensure the devices maintained the proper flow rate. Pumps were replaced as needed when they became too noisy or did not maintain flow. We maintained the devices continuously throughout the entire study and therefore no special cleaning/maintenance steps were needed when transferring the monitors between countries.

Data cleaning of real-time PM2.5 measurements

Baseline drift is a known issue with nephelometers and is usually a combination of 1) temperature changes, 2) vibration, and 3) soiling of the optics. Normal vibration has been ruled out as a problem for the MicroPEM and ECM during lab tests done at RTI using a sieve shaker while having a HEPA filter on the inlet. When the glass gets dirty it can scatter incident laser light back into the optical chamber where it can be measured by the detector; we minimized drift associated with the beam trap glass by monitoring the baseline voltage and cleaning the glass when needed. The main source of drift in field measurements in sub-Saharan Africa has therefore been temperature drift. It is not feasible to calculate unique temperature drift corrections for each individual device as this is time consuming; we relied on adjusting the baseline after the measurement period. For samples with small drift (<10 µg/m^3), we adjusted all

measurements by the absolute value of the smallest concentration e.g. if the minimum concentration during a monitoring period was -6 μg/m^3, then 6 μg/m^3 was added to all other real-time data points.

Samples with a negative measurement that was less than -10 μg/m^3 were analyzed for the number of datapoints within 10% of this minimum value, if <20 data points (reflecting 0.2% out of ~10000 measurements in the file) were near this value, then these data points were removed. The cause for this error was often associated with an incorrect relative humidity reading (e.g. >90% or at negative value) as the MicroPEM/ECM apply an RH correction to the PM concentration data. This issue was rare (~5% total files from Ghanian community, for example).

If there were >20 data points near the minimum value, the data was manually inspected for the source of the baseline drift. If it appears the baseline drifted by a constant amount during the sample period, then the baseline was adjusted accordingly. If only a portion of the baseline has drifted then we determined the cause the drift (e.g. rapid temperature swing, relative humidity swing, device dropped, etc). If there was a large and uneven baseline drift with no cause identified, then we invalidated the real-time data and conducted a re-sample if possible.

Assumptions for mixed effect models

In the mixed effect models, the assumptions for the covariance matrix are:

1. The error terms of the model are mean independent.
2. The regression model has a linear form.
3. Residual errors of the fitted model are unbiased estimates of the error terms
4. The model's errors are homoscedastic and uncorrelated

The residual degrees of freedom of the models was calculated as n-2, where n is the sample size.

Socioeconomic characteristic comparison by primary fuel type and community

Supplementary Table 1. Differences in socioeconomic characteristics between primary LPG users and exclusive polluting fuel users across all communities (N=256)

	**LPG (N=124)**	**Polluting (N=132)**	**p-value***
Age of primary cook (Mean (SD))	35 (11)	37 (12)	0.25
Age of child (Mean (SD))	3.1 (1.3)	3.1 (1.3)	0.88
Sex of child<br>Female	 27 (51%)	 44 (57%)	0.11
Age of household head (Mean	39 (11)	45 (12)	<0.001*

(SD))			
Participant is household head<br>Yes (female head)	 22 (18%)	 30 (22%)	0.36
Marital status<br>Married<br>Single<br>Cohabitating<br>Widowed	 72 (58%)<br>32 (26%)<br>13 (10%)<br>8 (6%)	 77 (56%)<br>28 (22%)<br>21 (17%)<br>11 (8%)	0.56
Household size (# members)<br>1-2<br>3-4<br>5-6<br>7+	 13 (10%)<br>44 (35%)<br>45 (36%)<br>23 (18%)	 8 (6%)<br>32 (23%)<br>41 (30%)<br>56 (41%)	0.002*
Financial security<br>Have enough money<br>Not quite enough<br>Definitely not enough	 43 (34%)<br>62 (50%)<br>20 (16%)	 22 (16%)<br>64 (47%)<br>51 (37%)	<0.001*
Highest education level<br>No formal education<br>Primary<br>Secondary/high school<br>University	 5 (4%)<br>17 (14%)<br>71 (57%)<br>32 (26%)	 10 (7%)<br>41 (30%)<br>72 (53%)<br>14 (10%)	0.001*

*t-test used for continuous data and chi-squared test used for categorical data

Supplementary Table 2. Differences in socioeconomic characteristics among LPG users by community (N=124)

	**Mbalmayo, Cameroon (N=43)**	**Obuasi, Ghana (N=40)**	**Eldoret, Kenya (N=41)**	**p-value**
Age of primary cook (Mean (SD))	38 (14)	35 (9)	31 (10)	0.49
Age of child (Mean (SD))	N/A	3.3 (1.3)	2.8 (1.4)	0.34
Sex of child<br>Female	 N/A	 15 (50%)	 12 (52%)	0.71
Age of household head (Mean (SD))	41 (14)	35 (9)	39 (9)	0.52
Participant is household head<br>Yes (female head)	 12 (28%)	 6 (15%)	 4 (10%)	0.07
Marital status<br>Married<br>Single<br>Cohabitating<br>Widowed	 17 (40%)<br>10 (23%)<br>11 (26%)<br>5 (12%)	 29 (73%)<br>7 (18%)<br>2 (5%)<br>2 (5%)	 26 (62%)<br>15 (36%)<br>0<br>1 (3%)	<0.001*
Household size (# members)<br>1-2<br>3-4<br>5-6<br>7+	 1 (2%)<br>12 (28%)<br>16 (37%)<br>14 (33%)	 10 (25%)<br>15 (38%)<br>14 (35%)<br>1 (3%)	 2 (5%)<br>17 (40%)<br>15 (35%)<br>8 (19%)	0.07
Financial security<br>Have enough money<br>Not quite enough<br>Definitely not enough	 10 (23%)<br>25 (58%)<br>8 (19%)	 17 (43%)<br>16 (40%)<br>7 (17%)	 16 (38%)<br>21 (50%)<br>5 (12%)	0.34
Highest education level<br>No formal education<br>Primary<br>Secondary/high school<br>University	 2 (5%)<br>10 (23%)<br>24 (56%)<br>7 (16%)	 6 (15%)<br>8 (20%)<br>21 (53%)<br>5 (13%)	 0<br>6 (14%)<br>14 (33%)<br>22 (52%)	<0.001*

*t-test used for continuous data and chi-squared test used for categorical data

Supplementary Table 3. Differences in socioeconomic characteristics among exclusive polluting cooking fuel users by community (N=132)

	**Mbalmayo, Cameroon (N=41)**	**Obuasi, Ghana (N=37)**	**Eldoret, Kenya (N=54)**	**p-value**
Age of primary cook (Mean (SD))	41 (13)	35 (11)	35 (11)	0.14
Age of child (Mean (SD))	N/A	3.0 (1.3)	3.2 (1.2)	0.56
Sex of child				0.22
Female	N/A	20 (61%)	24 (55%)	
Age of household head (Mean (SD))	48 (13)	42 (11)	45 (11)	0.34
Participant is household head				0.23
Yes (female head)	13 (30%)	8 (22%)	9 (16%)	
Marital status				<0.001*
Married	12 (28%)	25 (68%)	40 (70%)	
Single	12 (28%)	2 (5%)	14 (25%)	
Cohabitating	13 (30%)	8 (22%)	0	
Widowed	2 (4%)	2 (5%)	3 (5%)	
Household size (# members)				0.09
1-2	0	7 (19%)	1 (2%)	
3-4	4 (9%)	12 (32%)	16 (28%)	
5-6	10 (23%)	6 (16%)	25 (44%)	
7+	29 (67%)	12 (32%)	15 (26%)	
Financial security				0.08
Have enough money	3 (7%)	6 (16%)	13 (23%)	
Not quite enough	17 (40%)	17 (46%)	30 (53%)	
Definitely not enough	23 (53%)	14 (38%)	14 (25%)	
Highest education level				<0.001*
No formal education	0	7 (19%)	3 (5%)	
Primary	6 (14%)	7 (19%)	20 (35%)	
Secondary/high school	31 (72%)	23 (62%)	22 (39%)	
University	6 (14%)	0	12 (21%)	

*t-test used for continuous data and chi-squared test used for categorical data

Socioeconomic characteristic comparison for sub-sample with stove use monitoring

Supplementary Table 4. Comparing socioeconomic characteristics of study population with HAP data to sample with HAP data and stove use monitoring data

	**Households with HAP monitoring (N=256)**	**Households with HAP and stove monitoring (N=206)**	**p-value (X^2 test of independence)**
Age of household head (Mean (SD))	42 (12)	41 (12)	0.80 (t-test)
Head of household			0.87
Yes	52 (20%)	44 (21%)	
Marital status			0.55
Married	149 (57%)	105 (51%)	
Single	60 (23%)	55 (27%)	
Cohabitating	34 (13%)	30 (15%)	
Widowed	18 (7%)	18 (9%)	
Financial security			0.31
Have enough money	65 (25%)	42 (20%)	
Not quite enough	126 (48%)	98 (48%)	
Definitely not enough	71 (27%)	68 (32%)	
Highest education level			0.82
No formal education	15 (6%)	10 (5%)	

Primary<br>Secondary/high school<br>University	58 (23%)<br>141 (55%)<br>42 (16%)	53 (25%)<br>115 (56%)<br>30 (14%)	
Toilet in home<br>Yes	 96 (37%)	 76 (37%)	0.97
Primary lighting fuel<br>Electricity<br>Solar powered lantern/ flashlight/ oil lamp	 244 (93%)<br>18 (7%)	 193 (93%)<br>15 (7%)	0.97
Cooking location<br>In home (no separate room)<br>In home (separate room)<br>Outside home (separate room)<br>Veranda/porch<br>Outside home (open air)	 16 (7%)<br>86 (34%)<br>66 (26%)<br>66 (26%)<br>20 (8%)	 6 (5%)<br>41 (31%)<br>31 (23%)<br>45 (34%)<br>9 (7%)	0.56

Supplementary Table 5. Median (inner quartile range (IQR)) of study population ages and wearing compliance by community and primary cooking fuel

	**All communities (N=256)**		**Mbalmayo, Cameroon (N=84)**		**Obuasi, Ghana (N=77)**		**Eldoret, Kenya (N=95)**	
	**LPG (N=124)**	**Polluting (N=132)**	**LPG (N=43)**	**Polluting (N=41)**	**LPG (N=40)**	**Polluting (N=37)**	**LPG (N=41)**	**Polluting (N=54)**
Age of primary cook	33 (26, 39)	34 (28, 46)	36 (25, 53)	40 (31, 50)	34 (30, 38)	32 (27, 42)	30 (24, 36)	31 (30, 33)
Wearing compliance (cook)	48 (38, 60)	51 (40, 64)	39 (22, 54)	41 (29, 50)	48 (39, 57)	52 (44, 61)	58 (46, 66)	59 (48, 72)
Wearing compliance (child)	9 (3, 23)	20 (10, 31)	N/A	N/A	5 (1, 13)	17 (9, 27)	21 (9, 41)	20 (10, 35)

Cooking times during 24-hour monitoring period

While previous studies have reported shorter cooking times among households using LPG compared to polluting fuels,[4] this study finds that households stacking LPG and polluting fuels have a longer overall cooking time than those cooking exclusively with polluting fuels (Supplementary Figure 2). Thus, households use LPG for cooking may not reduce their total cooking time unless they exclusively cook with LPG.

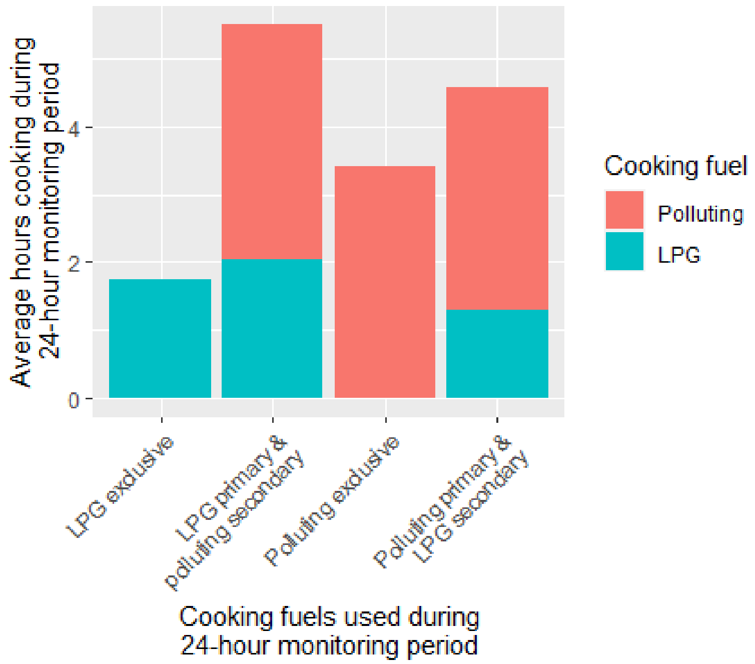

Supplementary Figure 2. Average cooking time during the 24-hour monitoring period by cooking fuels used according to SUM data

Integrating household air pollution and stove use measurements

Linear regression was used to characterise the relationship between PM2.5 and CO measurements for potential utility in estimating CO levels based on PM2.5 concentrations. Spearman's correlation coefficients (r) were calculated.

When assessing the bivariable relationship between average cooking time and $PM_{2.5}$ kitchen concentration during the 24-hour monitoring period, there was no association (R^2=0.00) among households cooking with LPG (Supplementary Figure 3). However, there was a positive association between increased cooking time and average 24-hour $PM_{2.5}$ concentration among households cooking with wood (R^2=0.02) and charcoal (R^2=0.12). There was no relationship between average cooking time and CO concentration among all fuel types during the 24-hour monitoring period (Supplementary Figure 4).

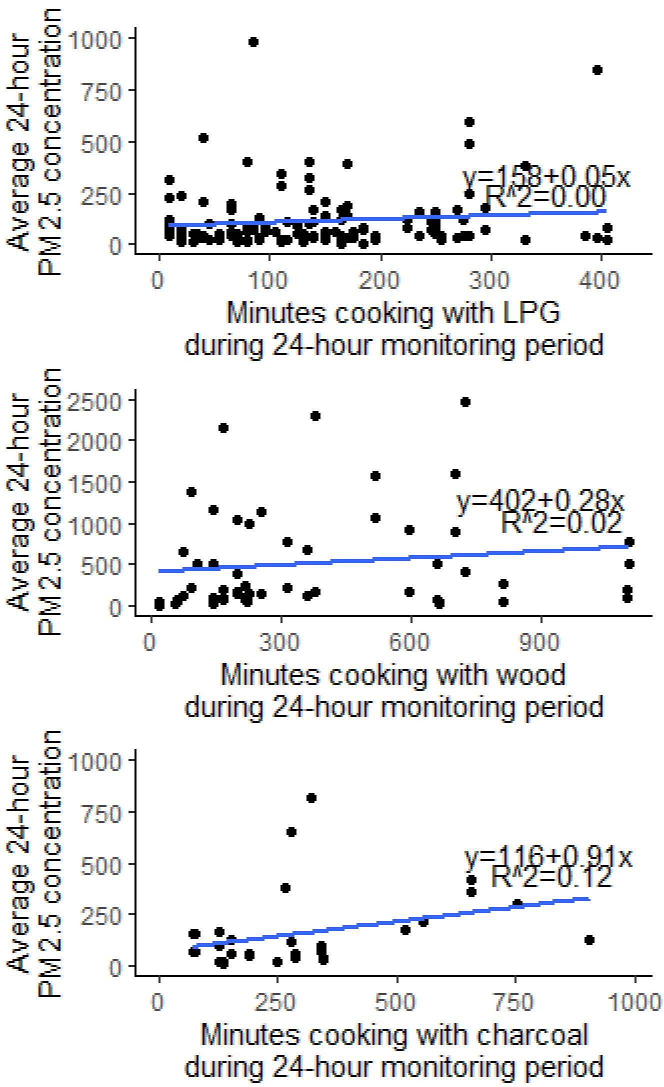

Supplementary Figure 3. Correlation between average 24-hour $PM_{2.5}$ concentration and cooking time during the monitoring period

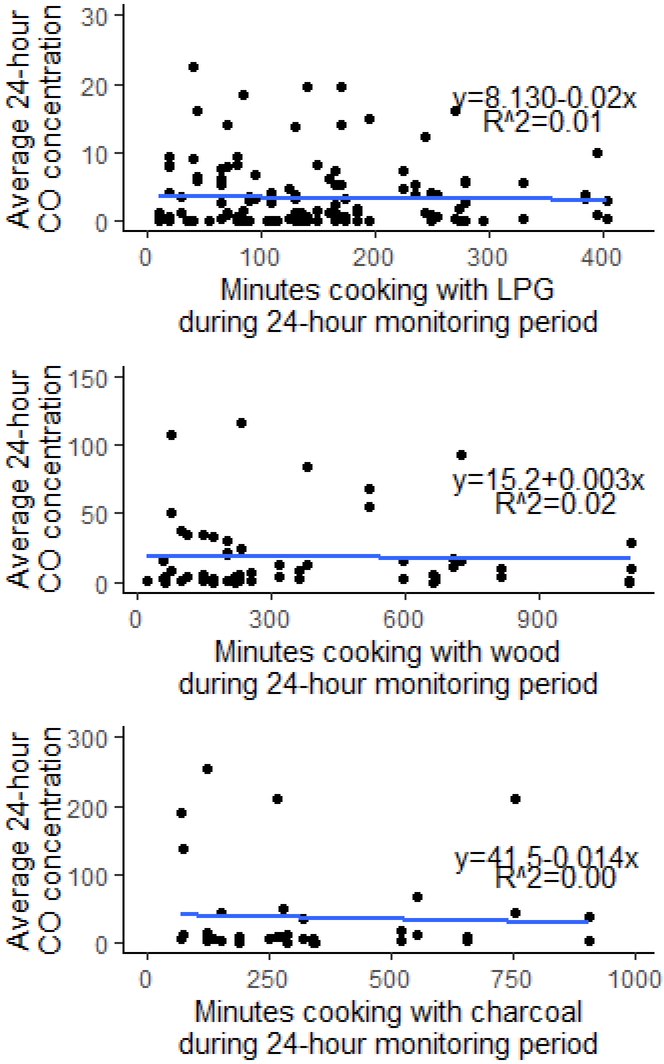

Supplementary Figure 4. Correlation between average 24-hour CO concentration and cooking time during the monitoring period

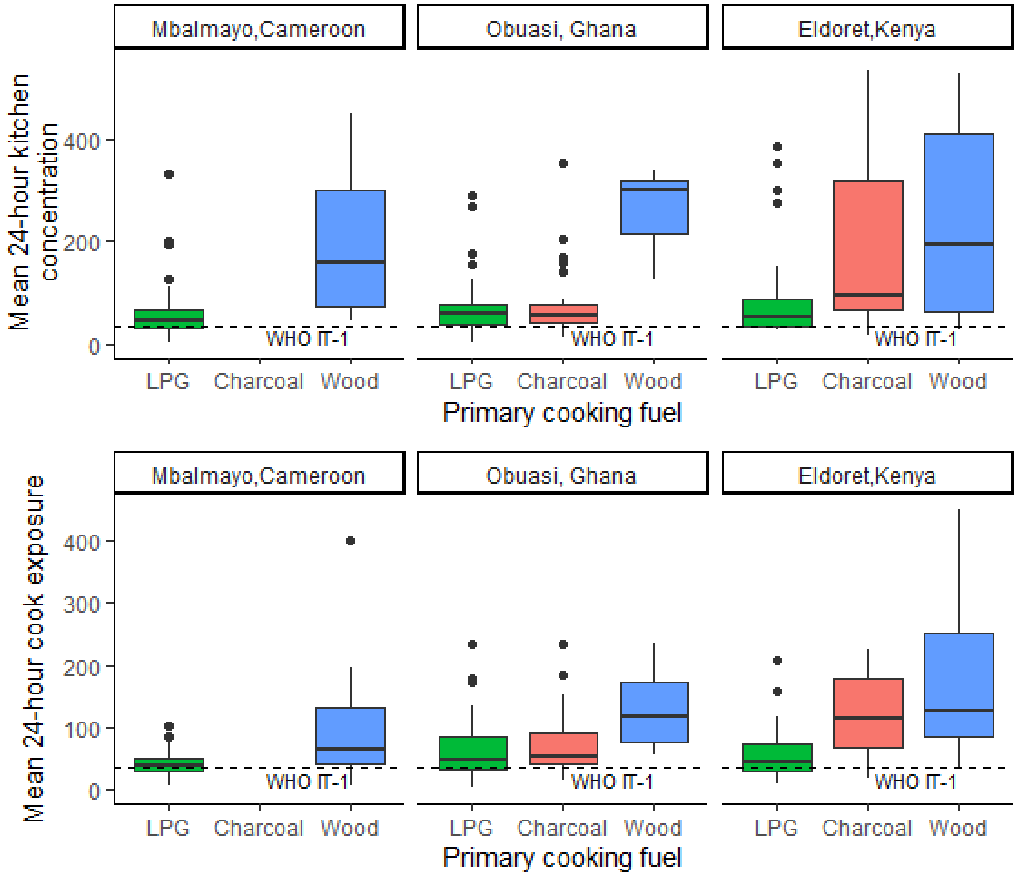

Supplementary Figure 5. Distribution of 24-hour $PM_{2.5}$ kitchen concentrations (top) and personal exposures (bottom) by primary cooking fuel type and community

Supplementary Table 6. Intraclass correlation coefficients for 24-hour $PM_{2.5}$ and CO kitchen concentrations and primary cook and child exposures

**Monitoring type**	**$PM_{2.5}$[1]**	**CO[2]**
**Kitchen concentrations**	0.11	0.08
**Cook exposures**	0.08	0.01
**Child exposures**	0.13	N/A

1. $PM_{2.5}$ = fine particulate matter
2. CO = carbon dioxide

Supplementary Table 7. Median (IQR)[1] 24-hour $PM_{2.5}$ and CO kitchen concentrations and personal (main cook, child) exposures stratified by primary cooking fuel type and community

**Primary cooking fuel**	**All communities (N=256)**			**Mbalmayo, Cam. (N=84)**			**Obuasi, Ghana (N=77)**			**Eldoret, Kenya (N=95)**		
**Kitchen concentrations (Median (IQR)[1])**												
	**N**	**$PM_{2.5}$ (ug/m^3)**	**CO (ppm)**	**N**	**$PM_{2.5}$ (ug/m^3)**	**CO (ppm)**	**N**	**$PM_{2.5}$ (ug/m^3)**	**CO (ppm)**	**N**	**$PM_{2.5}$ (ug/m^3)**	**CO (ppm)**
LPG	124	51 (34, 71)	0.65 (0.39, 1.07)	43	43 (31, 60)	0.99 (0.55, 1.78)	40	58 (39, 75)	0.31 (0.12, 0.81)	41	51 (34, 87)	1.06 (0.34, 3.31)
Charcoal	53	73 (46, 326)	8.18 (5.25, 11.10)	--	N/A	N/A	33	56 (40, 77)	1.77 (1.04, 2.99)	20	483 (89, 943)	15.81 (8.71, 28.72)
Wood	79	430 (159, 768)	14.5 (9.86, 19.20)	41	269 (152, 605)	5.88 (3.87, 8.95)	4	319 (257, 442)	N/A	34	600 (224, 898)	17.09 (11.13, 26.26)
**Cook exposures (Median (IQR)[1])**												
	**All communities (N=248)**			**Mbalmayo, Cam. (N=80)**			**Obuasi, Ghana (N=75)**			**Eldoret, Kenya (N=93)**		
LPG	119	42 (30, 61)	0.57 (0.37, 0.88)	43	40 (30, 51)	0.23 (0.11, 0.51)	38	49 (33, 85)	0.25 (0.12, 0.81)	38	44 (30, 73)	1.31 (0.65,2.65)
Charcoal	53	79 (48, 130)	3.76 (2.53, 4.99)	--	N/A	N/A	33	58 (44, 94)	1.52 (0.86, 2.67)	20	120 (73, 178)	3.76 (2.18, 6.51)
Wood	76	95 (56, 173)	2.52 (1.59, 3.45)	37	60 (43, 154)	0.70 (0.40, 1.20)	4	118 (76, 174)	N/A	35	113 (75, 273)	2.65 (1.74, 4.02)
**Child exposures (Median (IQR)[1])**												
	**All communities (N=124)**			**Mbalmayo, Cam. (N=0)**			**Obuasi, Ghana (N=62)**			**Eldoret, Kenya (N=62)**		
LPG	50	45 (30, 70)	N/A	--	N/A	N/A	29	45 (28, 57)	N/A	21	59 (30, 77)	N/A
Charcoal	47	59 (33, 83)	N/A	--	N/A	N/A	29	49 (28, 66)	N/A	18	78 (58, 107)	N/A
Wood	27	113 (61, 161)	N/A	--	N/A	N/A	4	70 (34, 117)	N/A	23	115 (69, 161)	N/A

1. IQR = inner quartile range

Supplementary Table 8. Differences in $PM_{2.5}$ and CO measurements (log-scale) by primary cooking fuel type and community using two-sample t-tests

	Kitchen concentrations											
Primary cooking fuel	Mbalmayo, Cam. (N=84)				Obuasi, Ghana (N=77)				Eldoret, Kenya (N=95)			
	Difference in $PM_{2.5}$ (95%CI) (log-scale)	p-value	Difference in CO (95%CI) (log-scale)	p-value	Difference in $PM_{2.5}$ (95%CI) (log-scale)	p-value	Difference in CO (95%CI) (log-scale)	p-value	Difference in $PM_{2.5}$ (95%CI) (log-scale)	p-value	Difference in CO (95%CI) (log-scale)	p-value
**Charcoal - LPG**	N/A	N/A	N/A	N/A	0.07 (-0.30, 0.43)	0.71	2.21 (1.21,3.22)	<0.001 *	1.58 (0.91, 2.25)	<0.001 *	2.41 (1.07, 3.76)	<0.001 *
**Wood - LPG**	1.87 (1.46, 2.29)	<0.001 *	1.64 (0.72, 2.56)	<0.001*	1.79 (0.70, 2.89)	0.01*	3.00 (1.30,4.70)	0.003 *	1.94 (1.49, 2.39)	<0.001 *	2.94 (1.71, 4.16)	<0.001 *
	**Cook exposures**											
	Mbalmayo, Cam. (N=80)				Obuasi, Ghana (N=75)				Eldoret, Kenya (N=93)			
	Difference in $PM_{2.5}$ (95%CI) (log-scale)	p-value	Difference in CO (95%CI) (log-scale)	p-value	Difference in $PM_{2.5}$ (95%CI) (log-scale)	p-value	Difference in CO (95%CI) (log-scale)	p-value	Difference in $PM_{2.5}$ (95%CI) (log-scale)	p-value	Difference in CO (95%CI) (log-scale)	p-value
**Charcoal - LPG**	N/A	N/A	N/A	N/A	0.20 (-0.15, 0.56)	0.25	1.70 (-0.28, 3.69)	0.09	0.88 (0.49, 1.28)	<0.001 *	1.22 (-1.08, 3.51)	0.29
**Wood - LPG**	0.69 (0.38, 1.00)	<0.001 *	2.33 (0.10, 4.56)	0.04*	0.85 (-0.07, 1.78)	0.06	2.50 (-2.70, 7.70)	0.16	1.10 (0.75, 1.46)	<0.001 *	2.01 (-0.19, 4.22)	0.07
	**Child exposures**											
	Mbalmayo, Cam. (N=0)				Obuasi, Ghana (N=62)				Eldoret, Kenya (N=62)			
	Difference in $PM_{2.5}$ (95%CI) (log-scale)	p-value	Difference in CO (95%CI) (log-scale)	p-value	Difference in $PM_{2.5}$ (95%CI) (log-scale)	p-value	Difference in CO (95%CI) (log-scale)	p-value	Difference in $PM_{2.5}$ (95%CI) (log-scale)	p-value	Difference in CO (95%CI) (log-scale)	p-value
**Charcoal - LPG**	N/A	N/A	N/A	N/A	0.14 (-0.35, 0.64)	0.56	N/A	N/A	0.47 (-0.09,1.04)	0.10	N/A	N/A
**Wood - LPG**	N/A	N/A	N/A	N/A	0.41 (-0.95, 1.77)	0.43	N/A	N/A	0.78 (0.21, 1.34)	0.01*	N/A	N/A

* statistically significant at 95% confidence level

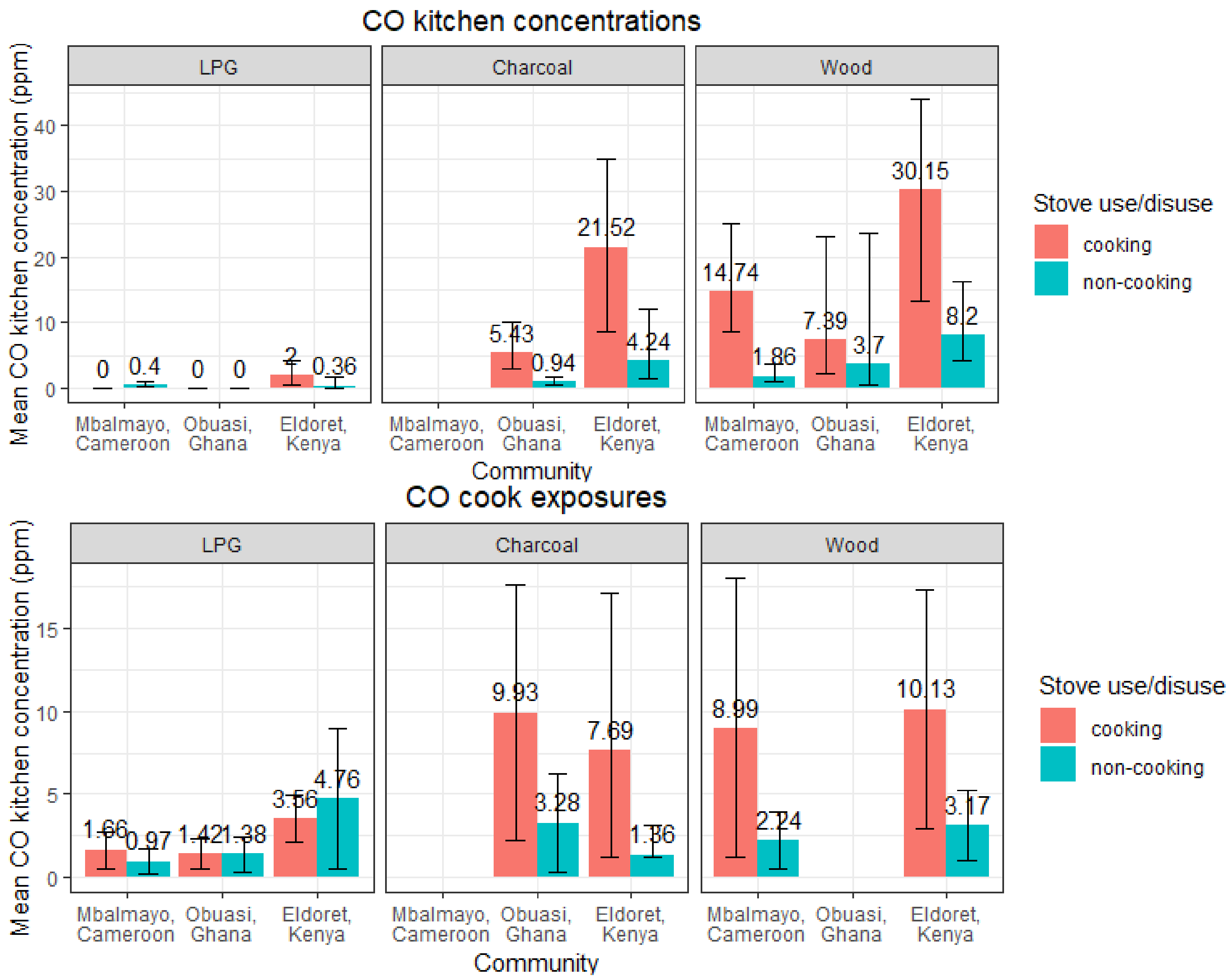

Supplementary Figure 7. Geometric mean CO kitchen concentrations (top) and cook exposures (bottom) during cooking and non-cooking periods stratified by community and primary cooking fuel type. Error bars represent the 95% confidence interval.

Supplementary Table 9. Differences in $PM_{2.5}$ measurements (log-scale) during cooking and non-cooking periods by primary cooking fuel type and community using two-sample t-tests

	**Kitchen concentrations**											
**Primary cooking fuel**	**Mbalmayo, Cam. (N=84)**				**Obuasi, Ghana (N=77)**				**Eldoret, Kenya (N=95)**			
	**Difference in $PM_{2.5}$ levels during cooking & non-cooking periods (95%CI) (log-scale)**	**p-value**	**Difference in CO levels during cooking & non-cooking periods (95%CI) (log-scale)**	**p-value**	**Difference in $PM_{2.5}$ levels during cooking & non-cooking periods (95%CI) (log-scale)**	**p-value**	**Difference in CO levels during cooking & non-cooking periods (95%CI) (log-scale)**	**p-value**	**Difference in $PM_{2.5}$ levels during cooking & non-cooking periods (95%CI) (log-scale)**	**p-value**	**Difference in CO levels during cooking & non-cooking periods (95%CI) (log-scale)**	**p-value**
**LPG**	1.37 (0.82, 1.93)	<0.001*	1.85 (0.47, 3.24)	0.001*	0.09 (-0.38, 0.57)	0.70	0.31 (-1.50, 2.12)	0.73	1.17 (0.38, 1.96)	0.006*	1.70 (-0.39, 3.80)	0.11
**Charcoal**	N/A	N/A	N/A	N/A	-0.55 (-1.14, 0.04)	0.07	1.75 (0.90, 2.61)	<0.001*	1.29 (-0.83, 3.41)	0.21	1.62 (0.08, 3.17)	0.04*
**Wood**	1.43 (0.74, 2.11)	<0.001*	2.07 (1.15, 2.99)	<0.001*	0.86 (-0.85, 2.58)	0.24	0.69 (-2.39, 3.77)	0.57	1.28 (0.02, 2.58)	0.04*	1.30 (0.17, 2.43)	0.03*
	**Cook exposures**											
	**Mbalmayo, Cam. (N=80)**				**Obuasi, Ghana (N=75)**				**Eldoret, Kenya (N=93)**			
	**Difference in $PM_{2.5}$ levels during cooking & non-cooking periods (95%CI) (log-scale)**	**p-value**	**Difference in CO levels during cooking & non-cooking periods (95%CI) (log-scale)**	**p-value**	**Difference in $PM_{2.5}$ levels during cooking & non-cooking periods (95%CI) (log-scale)**	**p-value**	**Difference in CO levels during cooking & non-cooking periods (95%CI) (log-scale)**	**p-value**	**Difference in $PM_{2.5}$ levels during cooking & non-cooking periods (95%CI) (log-scale)**	**p-value**	**Difference in CO levels during cooking & non-cooking periods (95%CI) (log-scale)**	**p-value**
**LPG**	0.96 (0.43, 1.48)	<0.001*	0.35 (-3.41, 4.13)	0.85	0.15 (-0.29, 0.59)	0.51	-1.67 (-4.06, 0.73)	0.17	0.89 (0.23, 1.53)	0.009*	0.41 (-2.61, 3.42)	0.78
**Charcoal**	N/A	N/A	N/A	N/A	-0.19 (-0.63, 0.24)	0.37	-0.54 (-4.18, 3.11)	0.77	1.67 (0.72, 2.61)	0.002*	0.69 (-2.81, 4.19)	0.67
**Wood**	1.21 (0.54, 1.89)	<0.001*	2.23 (-1.74, 6.20)	0.24	N/A	N/A	N/A	N/A	0.89 (0.03, 1.74)	0.04*	0.91 (-0.11, 1.93)	0.08
	**Child exposures**											
	**Mbalmayo, Cam. (N=0)**				**Obuasi, Ghana (N=62)**				**Eldoret, Kenya (N=62)**			
	**Difference in $PM_{2.5}$ levels during cooking & non-cooking periods (95%CI) (log-scale)**	**p-value**	**Difference in CO levels during cooking & non-cooking periods (95%CI) (log-scale)**	**p-value**	**Difference in $PM_{2.5}$ levels during cooking & non-cooking periods (95%CI) (log-scale)**	**p-value**	**Difference in CO levels during cooking & non-cooking periods (95%CI) (log-scale)**	**p-value**	**Difference in $PM_{2.5}$ levels during cooking & non-cooking periods (95%CI) (log-scale)**	**p-value**	**Difference in CO levels during cooking & non-cooking periods (95%CI) (log-scale)**	**p-value**
**LPG**	N/A	N/A	N/A	N/A	0.07 (-0.54, 0.68)	0.82	N/A	N/A	0.21 (-0.54, 0.96)	0.54	N/A	N/A
**Charcoal**	N/A	N/A	N/A	N/A	-0.13 (-0.73, 0.46)	0.65	N/A	N/A	0.50 (-1.02, 2.03)	0.47	N/A	N/A
**Wood**	N/A	N/A	N/A	N/A	N/A	N/A	N/A	N/A	0.08 (-0.86, 1.03)	0.85	N/A	N/A

* statistically significant at 95% confidence level

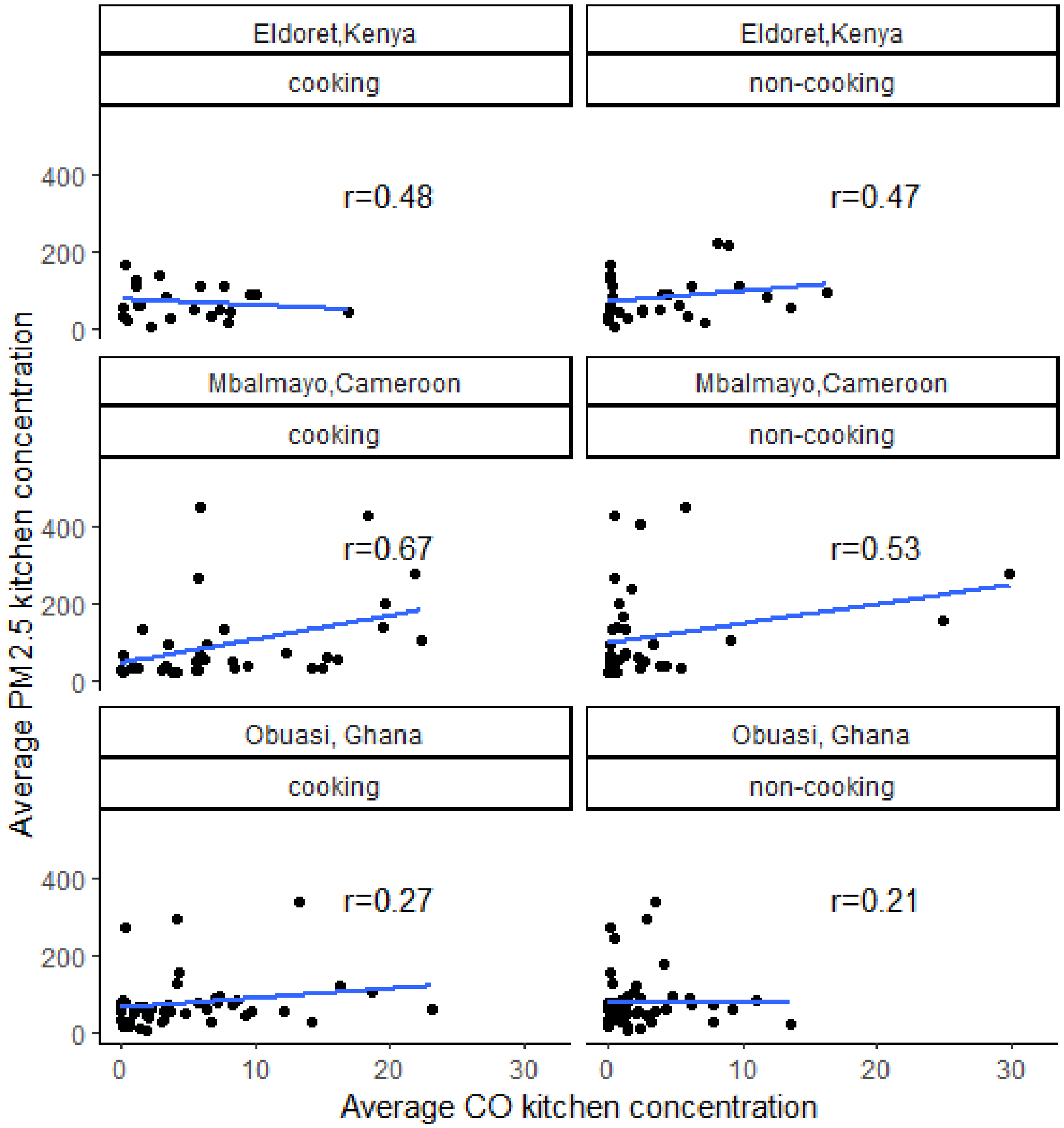

Supplementary Figure 8. Spearman correlation between mean $PM_{2.5}$ and CO kitchen concentrations during cooking and non-cooking periods by community

Exceedance of WHO thresholds

Supplementary Table 10. Percent of HAP measurements exceeding WHO thresholds by monitoring type and community

**Monitoring Type**	**Community**	**Primary cooking fuel**	**Percentage of $PM_{2.5}$ measurements above WHO interim-1 target (35 ug/m3)**	**Percentage of CO measurements above WHO AQG[1] (6.11 ppm)**
Kitchen	All communities	LPG	48	9
		charcoal	51	27
		wood	45	21
	Mbalmayo, Cameroon	LPG	38	9
		wood	44	21
	Obuasi, Ghana	LPG	66	5
		charcoal	58	14
		wood	50	14
	Eldoret, Kenya	LPG	38	20
		charcoal	44	39
		wood	44	39
Cook	All communities	LPG	38	4
		charcoal	58	17
		wood	40	7
	Mbalmayo, Cameroon	LPG	28	3
		wood	32	6
	Obuasi, Ghana	LPG	60	2
		charcoal	75	13
		wood	81	5
	Eldoret, Kenya	LPG	29	9
		charcoal	41	21
		wood	43	12
Child	All communities	LPG	48	N/A

		charcoal	44	N/A
		wood	59	N/A
	Eldoret, Kenya	LPG	38	N/A
		charcoal	40	N/A
		wood	56	N/A
	Obuasi, Ghana	LPG	52	N/A
		charcoal	49	N/A
		wood	62	N/A

Cook:kitchen ratio

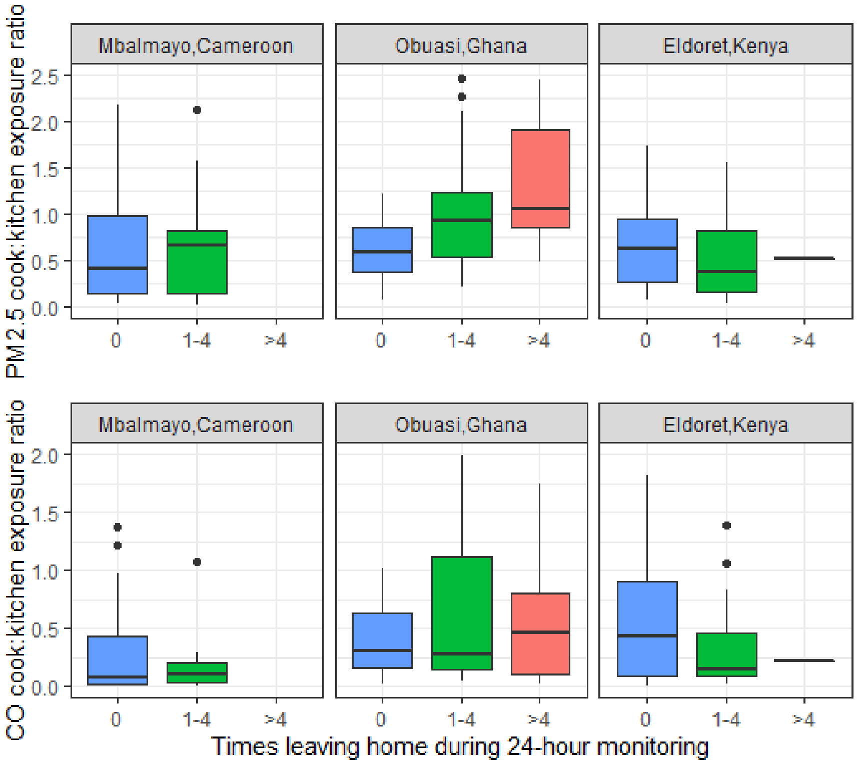

Supplementary Figure 8. Median $PM_{2.5}$ (top) and CO (bottom) cook:kitchen ratio by number of times leaving the household during the HAP monitoring period

Travel time to nearest main road

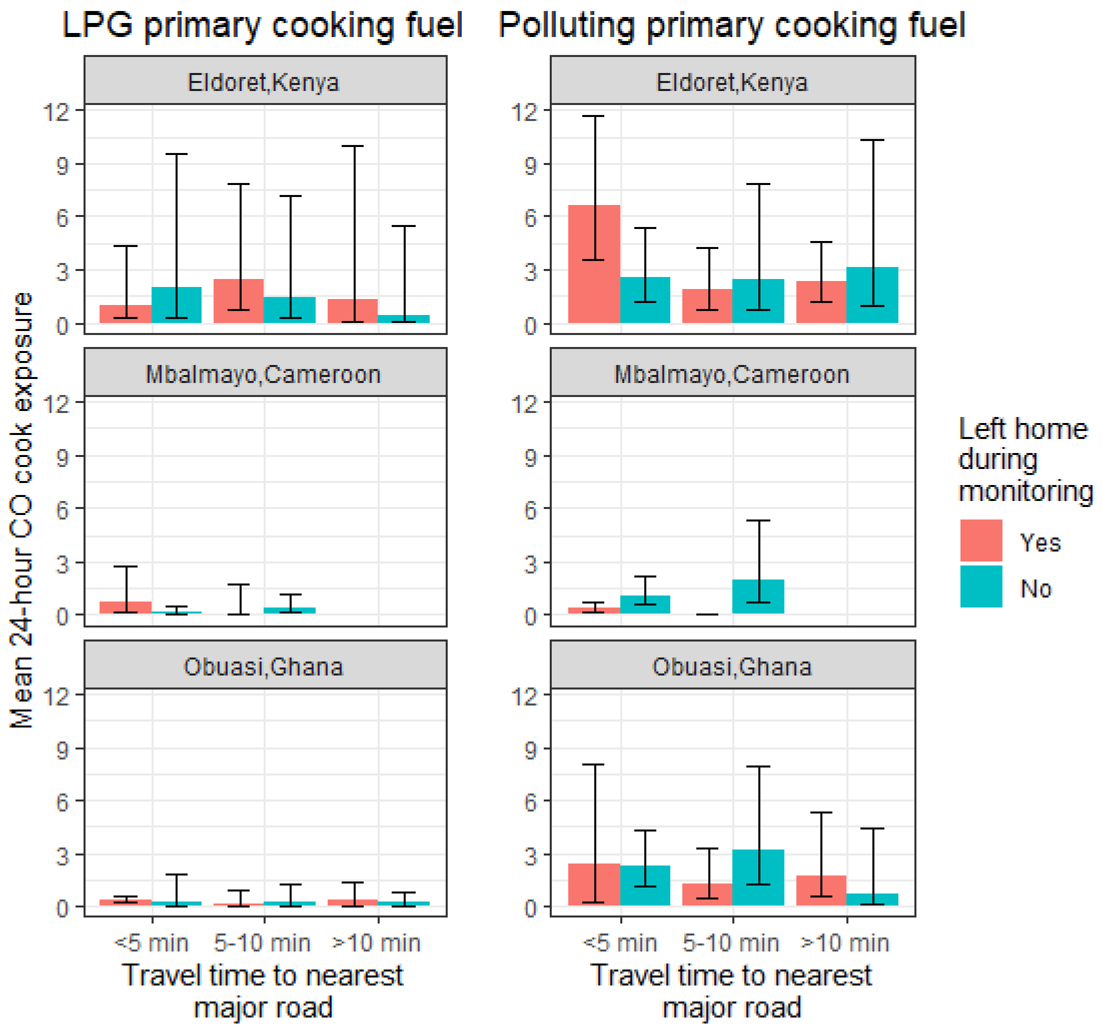

Supplementary Figure 9. Average 24-hour CO cook exposure among households primarily cooking with LPG (left) and households primarily cooking with polluting fuels (right) by travel distance to nearest major road and whether they left their home during the 24-hour monitoring

Fuel stacking

Exclusive use of LPG only led to marginally lower average $PM_{2.5}$ (50 μg/m^3 95%CI:[41,61])) and CO exposures (0.48 ppm 95%CI:[0.28,0.81]) relative to those stacking LPG with a polluting fuel (54 μg/m^3 95%CI:[47,63] and 0.65 95%CI:[0.39,1.07], respectively) (Supplementary Table 3). This indicates that efforts to reduce fuel stacking may not significantly lower $PM_{2.5}$ exposures if individuals are exposed to high outdoor $PM_{2.5}$ levels. To this end, a complete transition to LPG or other clean fuels likely lead to a more significant reduction in $PM_{2.5}$ exposures only once ambient air pollution is reduced.

Supplementary Table 11. Average 24-hour $PM_{2.5}$ and CO levels by cooking location and community

Kitchen concentrations (GM[1] (95% CI))										
Primary cooking fuel	Cooking location	Mbalmayo, Cameroon			Obuasi, Ghana			Eldoret, Kenya		
		N	$PM_{2.5}$ (ug/m^3)	CO (ppm)	N	$PM_{2.5}$ (ug/m^3)	CO (ppm)	N	$PM_{2.5}$ (ug/m^3)	CO (ppm)
LPG	In main house: separate room	16	53 (40, 70)	0.59 (0.24, 1.47)	6	70 (24, 202)	1.57 (0.57, 4.32)	25	61 (47, 81)	1.50 (0.46, 4.87)
	On veranda/porch	--	N/A	N/A	23	49 (35, 67)	0.26 (0.10, 0.68)	--	N/A	N/A
	In open air	--	N/A	N/A	3	70 (55, 88)	0.06 (0.01, 0.39)	--	N/A	N/A
Charcoal	In main house: separate room	--	N/A	N/A	--	N/A	N/A	6	56 (32, 98)	3.53 (1.29, 9.72)
	Outside of main house: in separate room	--	N/A	N/A	--	N/A	N/A	9	786 (573, 1077)	20.09 (11.02, 36.63)
	On veranda/porch	--	N/A	N/A	18	58 (45, 75)	1.95 (1.12, 3.42)	--	N/A	N/A
Wood	In main house: separate room	--	N/A	N/A	--	N/A	N/A	4	90 (25, 335)	6.31 (2.30, 17.32)
	On veranda/porch	10	234 (114, 476)	10.35 (5.46, 19.62)	--	N/A	N/A	--	N/A	N/A
	Outside of main house: in a separate room	13	478 (329, 694)	3.42 (1.70, 6.88)	--	N/A	N/A	13	689 (443, 1072)	24.61 (11.00, 55.09)
Cook exposures (GM[1] (95% CI))										
		Mbalmayo, Cameroon			Obuasi, Ghana			Eldoret, Kenya		
LPG	In main house: separate room	16	39 (28, 55)	0.76 (0.24, 2.47)	8	49 (18, 135)	0.46 (0.08, 2.56)	26	44 (32, 59)	1.41 (0.58, 3.45)
	On veranda/porch	--	N/A	N/A	30	49 (36, 66)	0.20 (0.11, 0.38)	--	N/A	N/A
	In open air	--	N/A	N/A	6	49 (31, 78)	0.06 (0.01, 0.45)	--	N/A	N/A
Charcoal	In main house: separate room	--	N/A	N/A	--	N/A	N/A	6	73 (37, 143)	4.04 (1.31, 12.46)
	Outside of main house: in separate room	--	N/A	N/A	--	N/A	N/A	10	149 (19, 186)	3.10 (1.72, 5.60)
	On veranda/porch	--	N/A	N/A	13	67 (51, 87)	1.13 (0.36, 3.60)	--	N/A	N/A
Wood	In main house: separate room	--	N/A	N/A	--	N/A	N/A	6	78 (36, 168)	4.01 (1.78, 9.04)
	On veranda/porch	10	61 (43, 87)	0.72 (0.09, 5.76)	--	N/A	N/A	--	N/A	N/A
	Outside of main house: in separate room	15	60 (40, 92)	0.45 (0.15, 1.30)	--	N/A	N/A	13	201 (143, 281)	2.40 (1.15, 5.01)

Supplementary Table 12. Average 24-hour $PM_{2.5}$ and CO levels by cooking location, community and fuel stacking

Kitchen concentrations (GM[1] (95% CI))												
**Cooking fuel(s) used**	**All communities (N=246)**			**Mbalmayo, Cam. (N=82)**			**Obuasi, Ghana (N=71)**			**Eldoret, Kenya (N=93)**		
	**N**	**$PM_{2.5}$ (ug/m^3)**	**CO (ppm)**	**N**	**$PM_{2.5}$ (ug/m^3)**	**CO (ppm)**	**N**	**$PM_{2.5}$ (ug/m^3)**	**CO (ppm)**	**N**	**$PM_{2.5}$ (ug/m^3)**	**CO (ppm)**
LPG primary	115	54 (47, 63)	0.65 (0.39, 1.07)	35	47 (35, 63)	0.99 (0.55, 1.78)	39	54 (42, 70)	0.31 (0.12, 0.81)	41	61 (49, 76)	1.06 (0.34, 3.31)
LPG exclusive[2]	75	50 (41, 61)	0.48 (0.28, 0.81)	22	45 (29, 69)	0.71 (0.26,1.97)	30	52 (38, 71)	0.42 (0.21,0.85)	23	53 (42, 68)	0.49 (0.15,1.64)
Charcoal primary	53	109 (77, 155)	8.18 (5.25, 11.10)	--	N/A	N/A	33	56 (44, 72)	1.77 (1.04, 2.99)	20	297 (163, 538)	15.81 (8.71, 28.72)
Charcoal exclusive	28	165 (75, 364)	9.20 (5.50, 15.38)	--	N/A	N/A	18	51 (38,68)	4.61 (1.97, 10.81)	10	317 (117, 857)	13.65 (8.64, 21.55)
Wood primary	67	402 (284, 481)	14.5 (9.86, 19.20)	35	290 (199, 423)	5.88 (3.87, 8.95)	4	314 (154, 643)	N/A	28	501 (339, 740)	17.09 (11.13, 26.26)
Wood exclusive	23	300 (176, 510)	12.64 (7.61, 20.98)	18	298 (169, 523)	8.27 (4.08, 16.82)	--	N/A	N/A	5	727 (436, 1,212)	22.13 (12.07, 40.57)
**Cook exposures (GM[1] (95% CI))**												
	**All communities (N=223)**			**Mbalmayo, Cam. (N=63)**			**Obuasi, Ghana (N=67)**			**Eldoret, Kenya (N=89)**		
LPG primary	108	43 (38, 48)	0.57 (0.37, 0.88)	32	36 (30, 42)	0.23 (0.11, 0.51)	38	47 (36, 61)	0.25 (0.12, 0.81)	38	45 (36, 56)	1.31 (0.65,2.65)
LPG exclusive	71	44 (37, 52)	0.44 (1.74, 4.55)	20	36 (29, 44)	0.50 (0.17,1.43)	29	57 (43, 75)	0.30 (0.16,0.55)	20	40 (27, 60)	1.07 (0.48,2.40)
Charcoal primary	51	77 (63, 95)	3.76 (2.53, 4.99)	--	N/A	N/A	29	59 (46, 74)	1.52 (0.86, 2.67)	22	110 (80, 150)	3.76 (2.18, 6.51)
Charcoal exclusive	7	63 (36, 109)	2.31 (0.95, 5.63)	--	N/A	N/A	3	49 (16, 151)	1.66 (0.16, 17.35)	4	76 (42, 136)	2.72 (1.23,6.07)
Wood primary	64	98 (80, 119)	2.52 (1.59, 3.45)	31	66 (51, 86)	0.70 (0.40, 1.20)	4	114 (62, 211)	N/A	29	146 (112, 191)	2.65 (1.74, 4.02)
Wood exclusive	31	77 (60, 100)	1.16 (0.49, 2.74)	24	64 (49, 83)	0.52 (0.09, 3.02)	1	154 (N/A)	N/A	6	152 (105, 219)	2.18 (0.99, 4.83)
**Child exposures (GM[1] (95% CI))**												
	**All communities (N=119)**			**Mbalmayo, Cam. (N=0)**			**Obuasi, Ghana (N=59)**			**Eldoret, Kenya (N=60)**		
LPG primary	50	47 (37, 58)	N/A	--	N/A	N/A	31	41 (31, 53)	N/A	21	56 (38, 83)	N/A
LPG exclusive	34	49 (36, 66)	N/A	--	N/A	N/A	21	39 (28, 54)	N/A	12	71 (39, 129)	N/A
Charcoal primary	47	59 (45, 79)	N/A	--	N/A	N/A	28	46 (32, 67)	N/A	18	90 (61, 132)	N/A
Charcoal exclusive	25	77 (43, 135)	N/A	--	N/A	N/A	5	43 (28, 66)	N/A	7	115 (51, 262)	N/A
Wood primary	27	107 (76, 150)	N/A	--	N/A	N/A	4	61 (25, 148)	N/A	23	118 (82,169)	N/A
Wood exclusive	10	205 (119, 353)	N/A	--	N/A	N/A	1	161 (N/A)	N/A	9	210 (115, 386)	N/A

Real-time PM2.5 kitchen concentration measurements

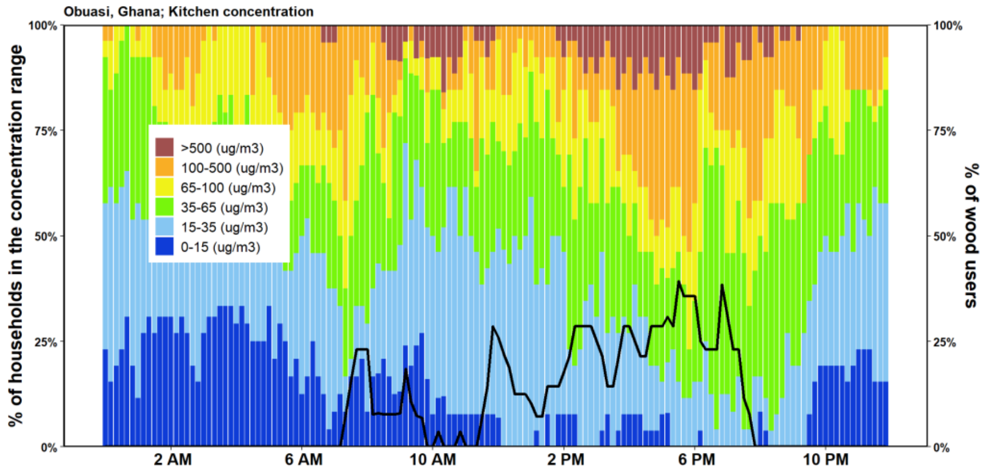

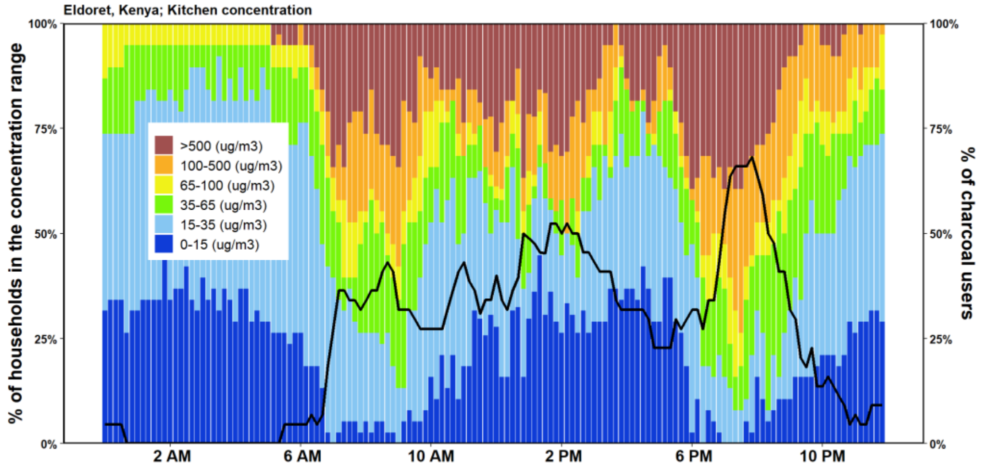

Supplementary Figure 10. Real-time $PM_{2.5}$ kitchen concentration measurements among households primarily cooking with wood in Obuasi, Ghana (top) and charcoal in Eldoret, Kenya (bottom).

Real-time PM2.5 cook exposure measurements

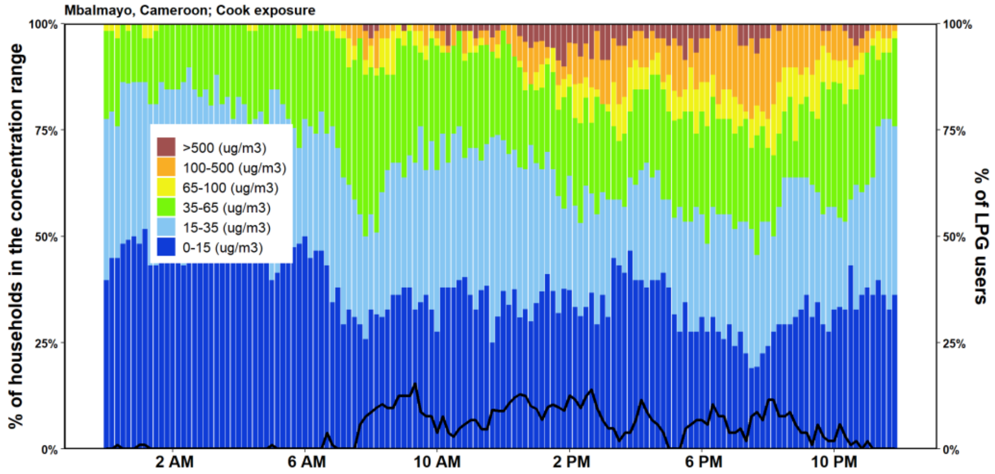

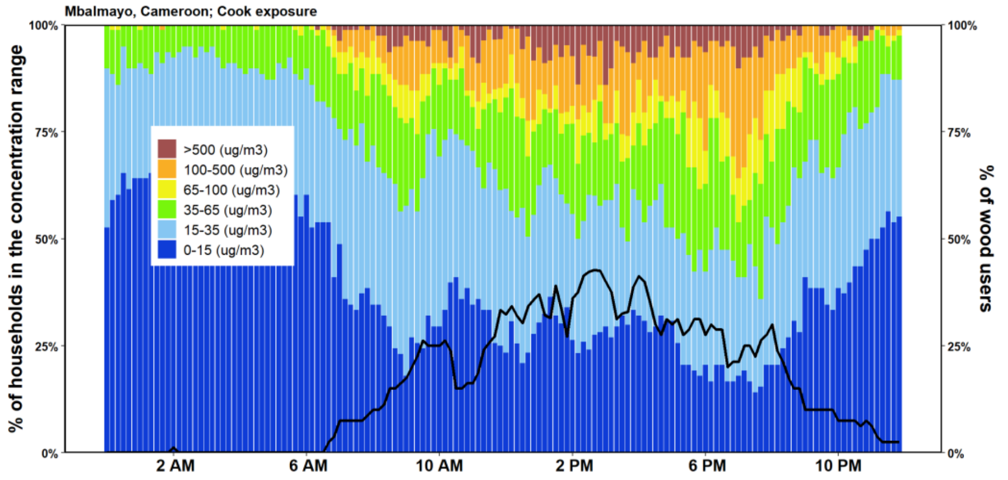

Supplementary Figure 11. Real-time $PM_{2.5}$ cook exposure measurements in Mbalmayo, Cameroon among households primarily cooking with LPG (top) and wood (bottom).

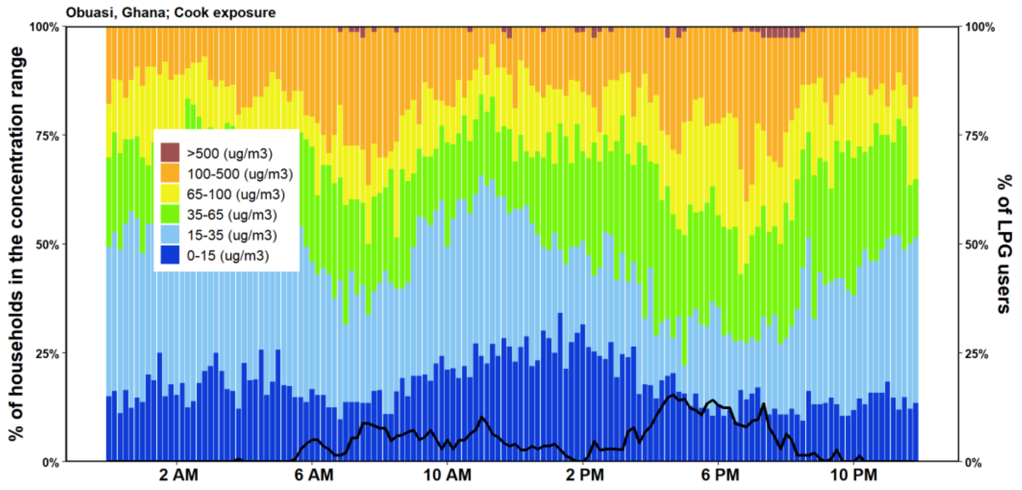

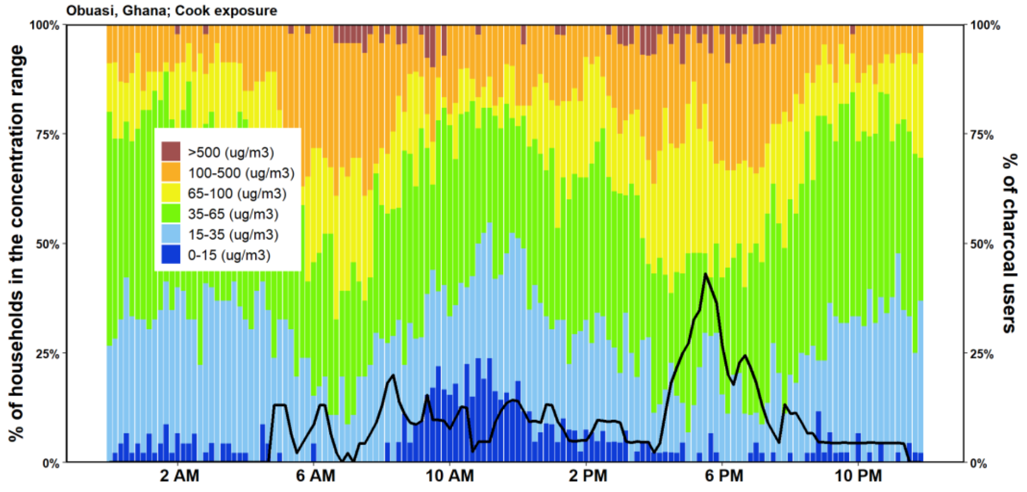

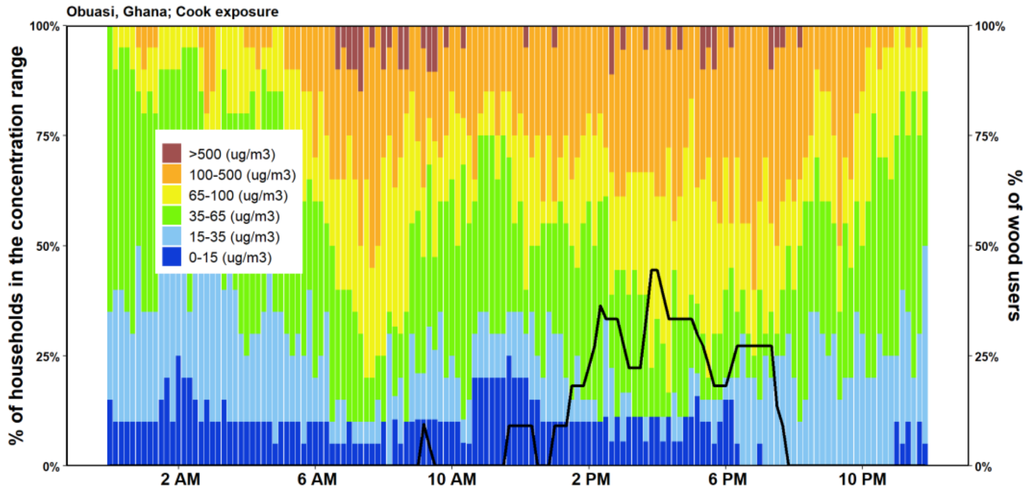

Supplementary Figure 12. Real-time $PM_{2.5}$ cook exposure measurements in Obuasi, Ghana among households primarily cooking with LPG (top), charcoal (middle) and wood (bottom).

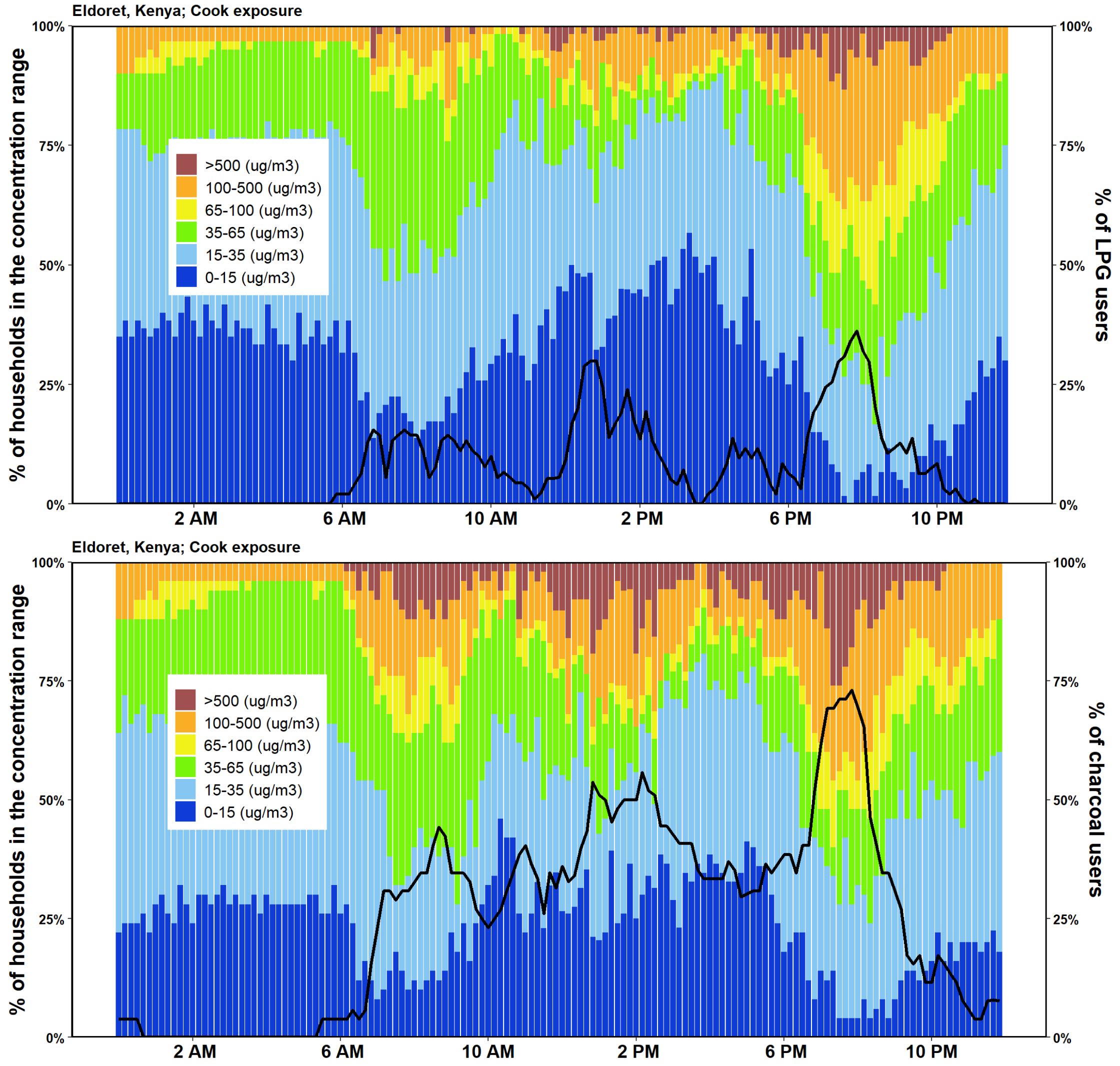

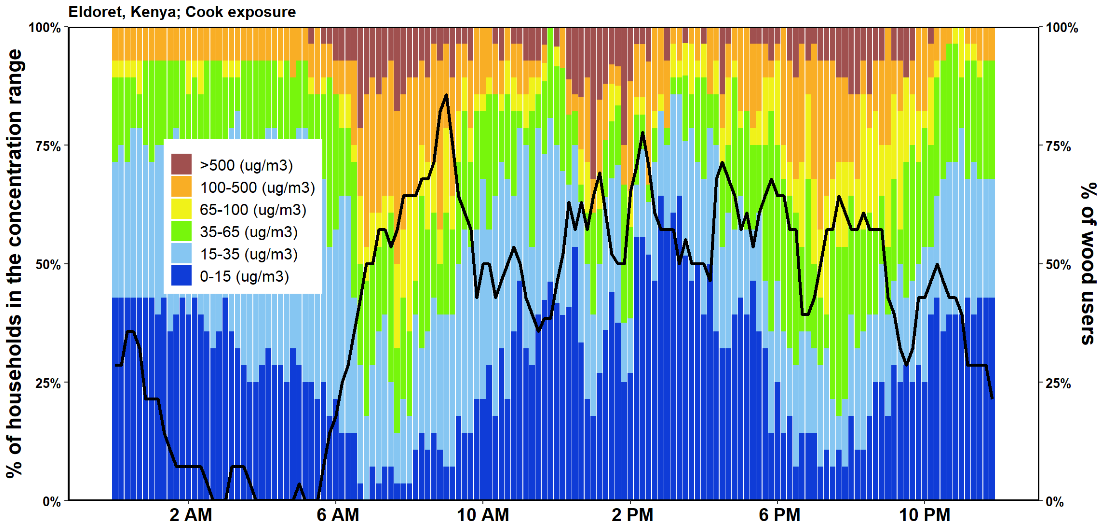

Supplementary Figure 13. Real-time $PM_{2.5}$ cook exposure measurements in Eldoret, Kenya among households primarily cooking with LPG (top), charcoal (middle) and wood (bottom).

Real-time carbon monoxide kitchen concentration measurements

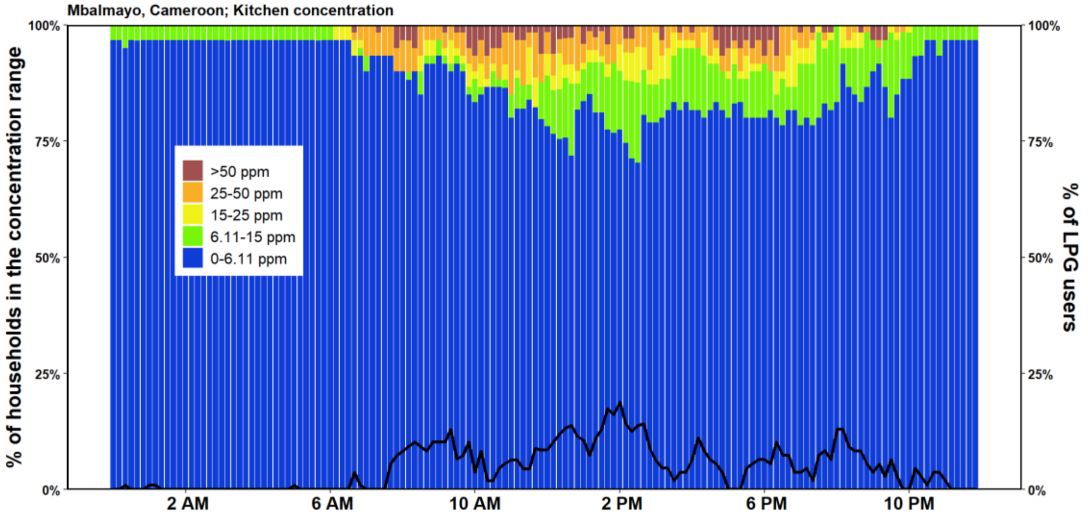

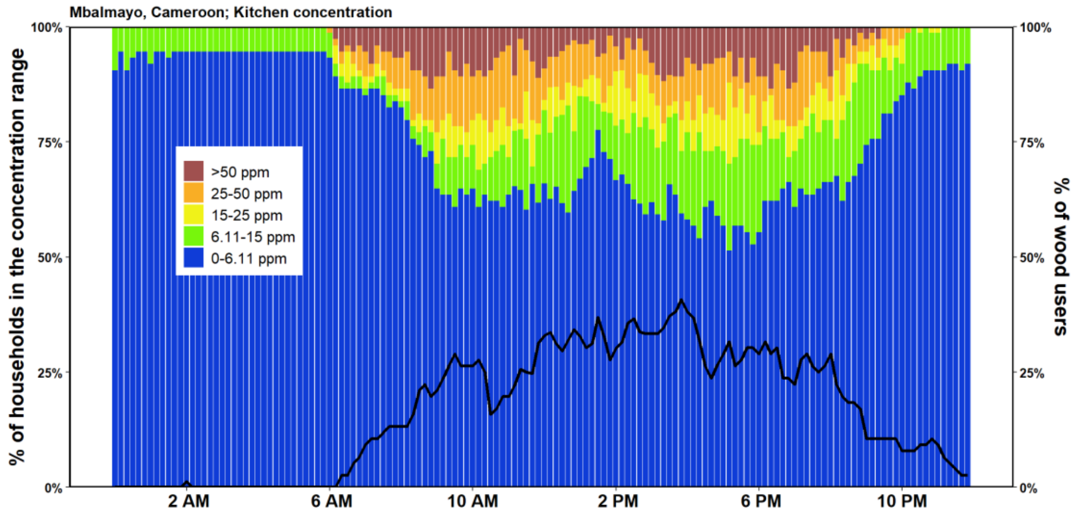

Supplementary Figure 14. Real-time CO kitchen concentration measurements in Mbalmayo, Cameroon among households primarily cooking with LPG (top) and wood (bottom).

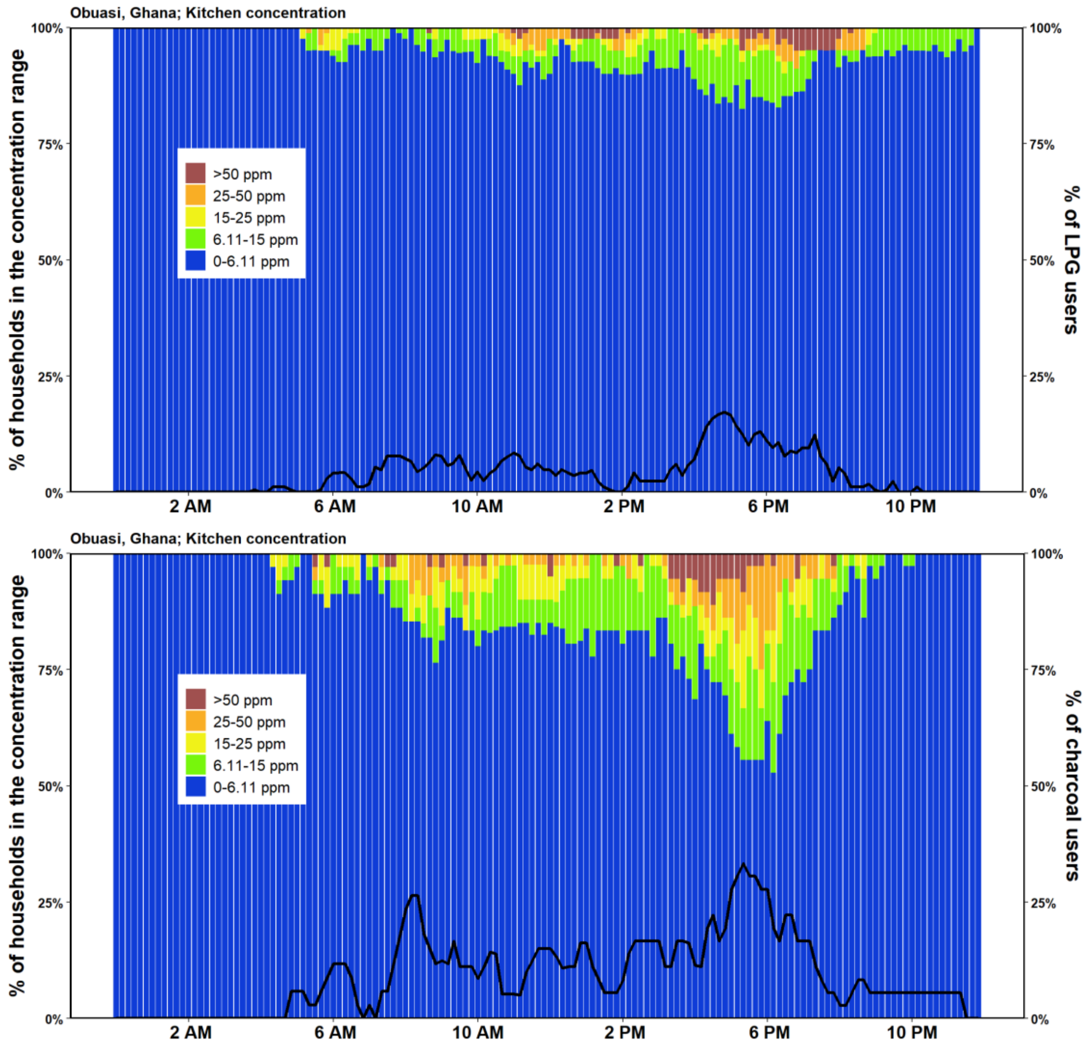

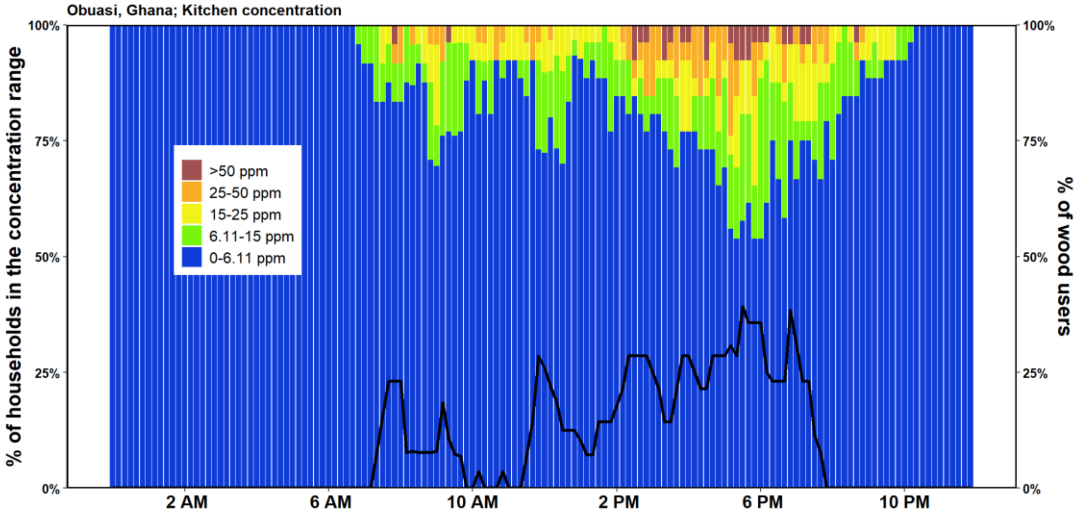

Supplementary Figure 15. Real-time CO kitchen concentration measurements in Obuasi, Ghana among households primarily cooking with LPG (top), charcoal (middle) and wood (bottom).

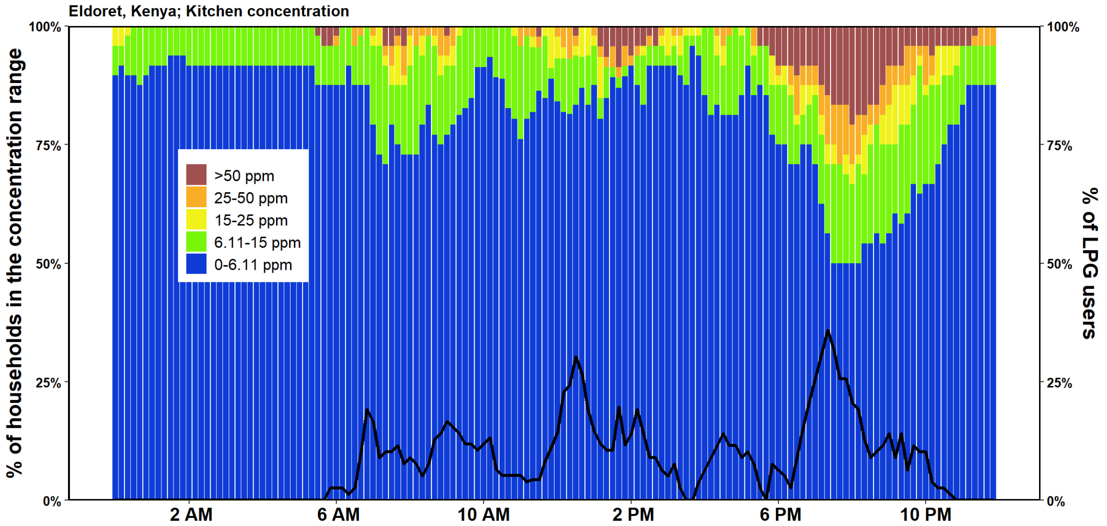

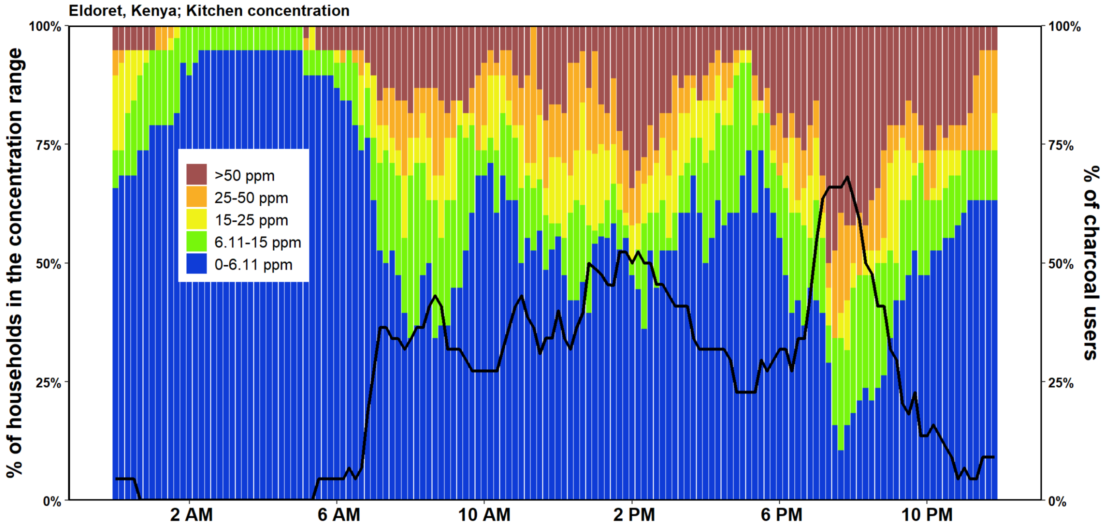

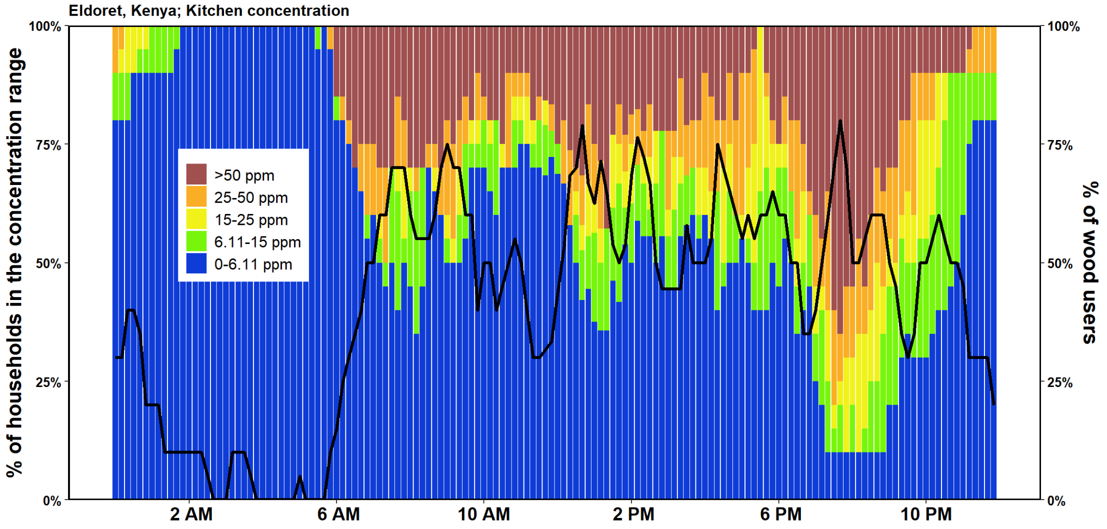

Supplementary Figure 16. Real-time $PM_{2.5}$ kitchen concentration measurements in Eldoret, Kenya among households primarily cooking with LPG (top), charcoal (middle) and wood (bottom).

Real-time carbon monoxide cook exposure measurements

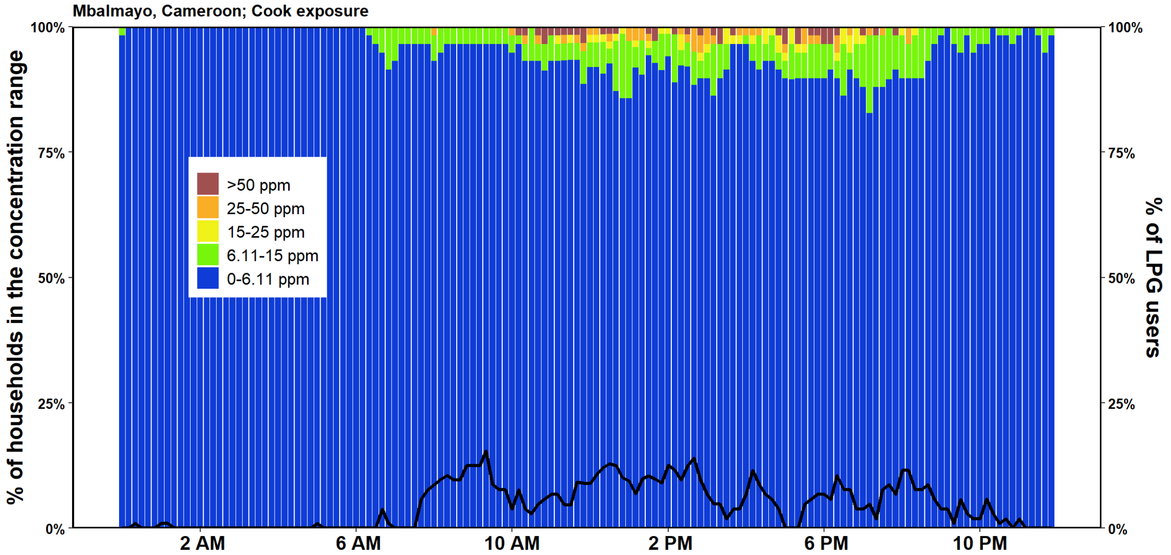

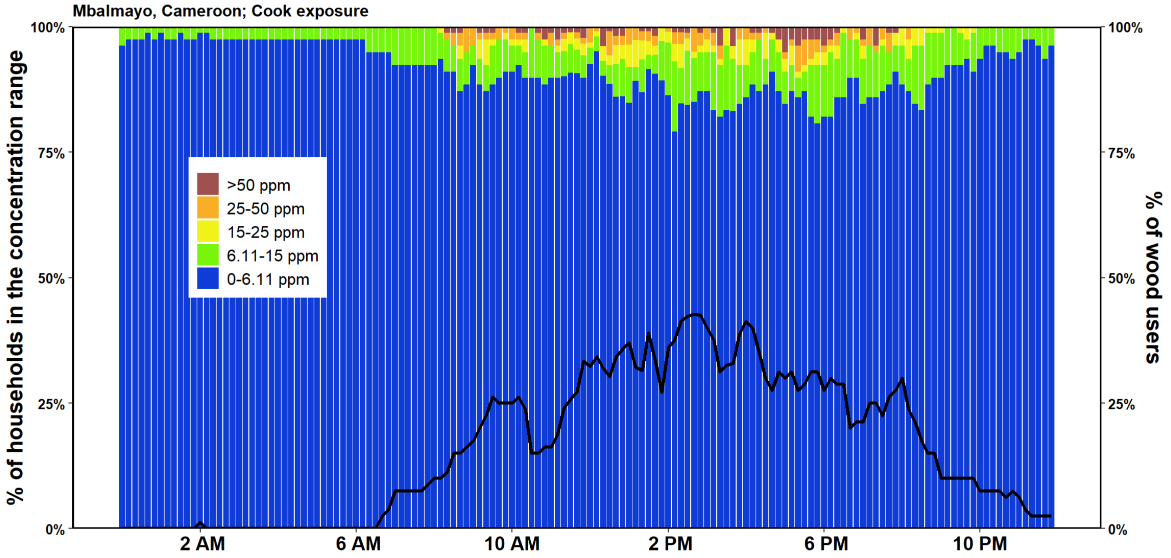

Supplementary Figure 17. Real-time CO cook exposure measurements in Mbalmayo, Cameroon among households primarily cooking with LPG (top) and wood (bottom).

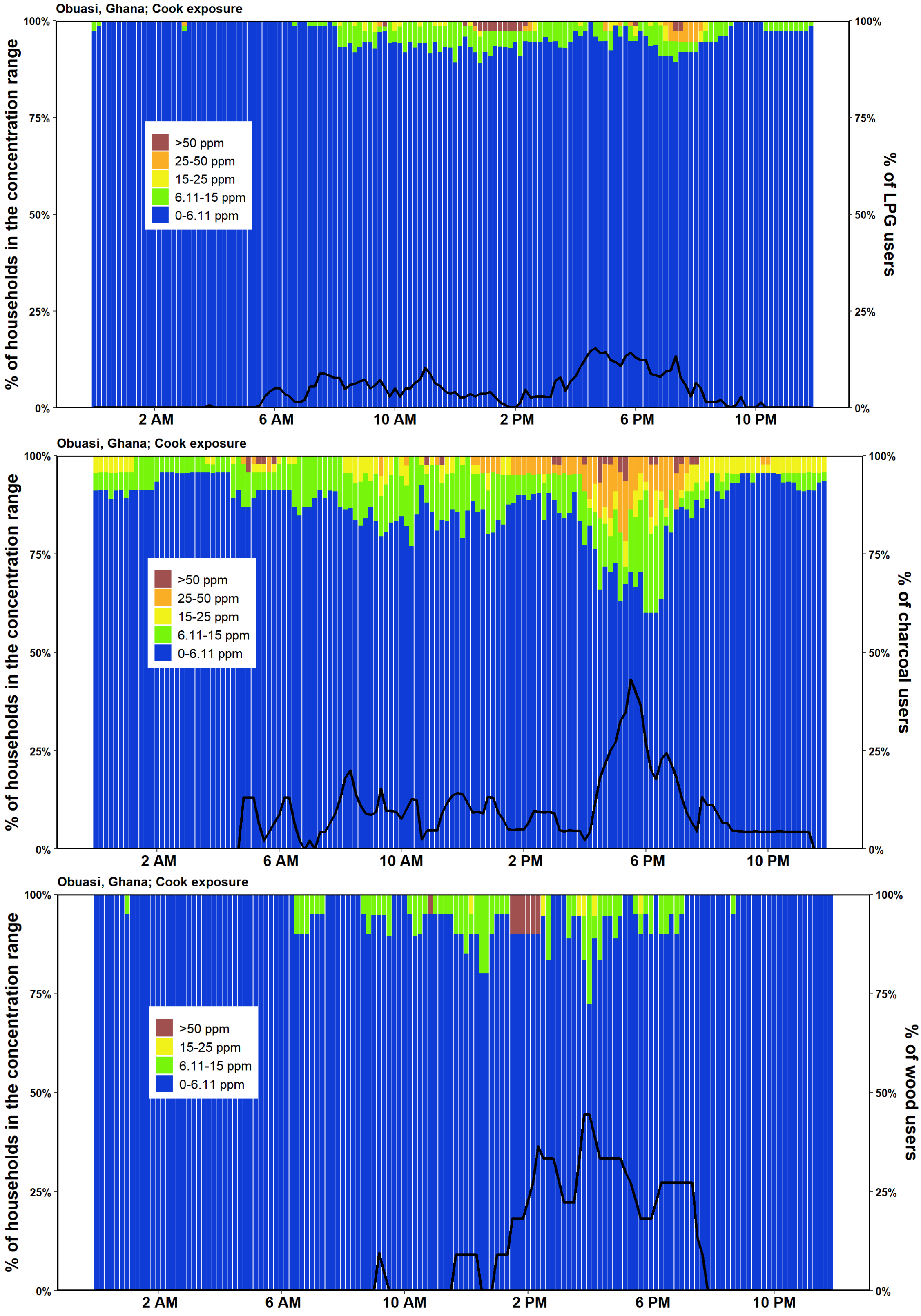

Supplementary Figure 18. Real-time CO cook exposure measurements in Obuasi, Ghana among households primarily cooking with LPG (top), charcoal (middle) and wood (bottom).

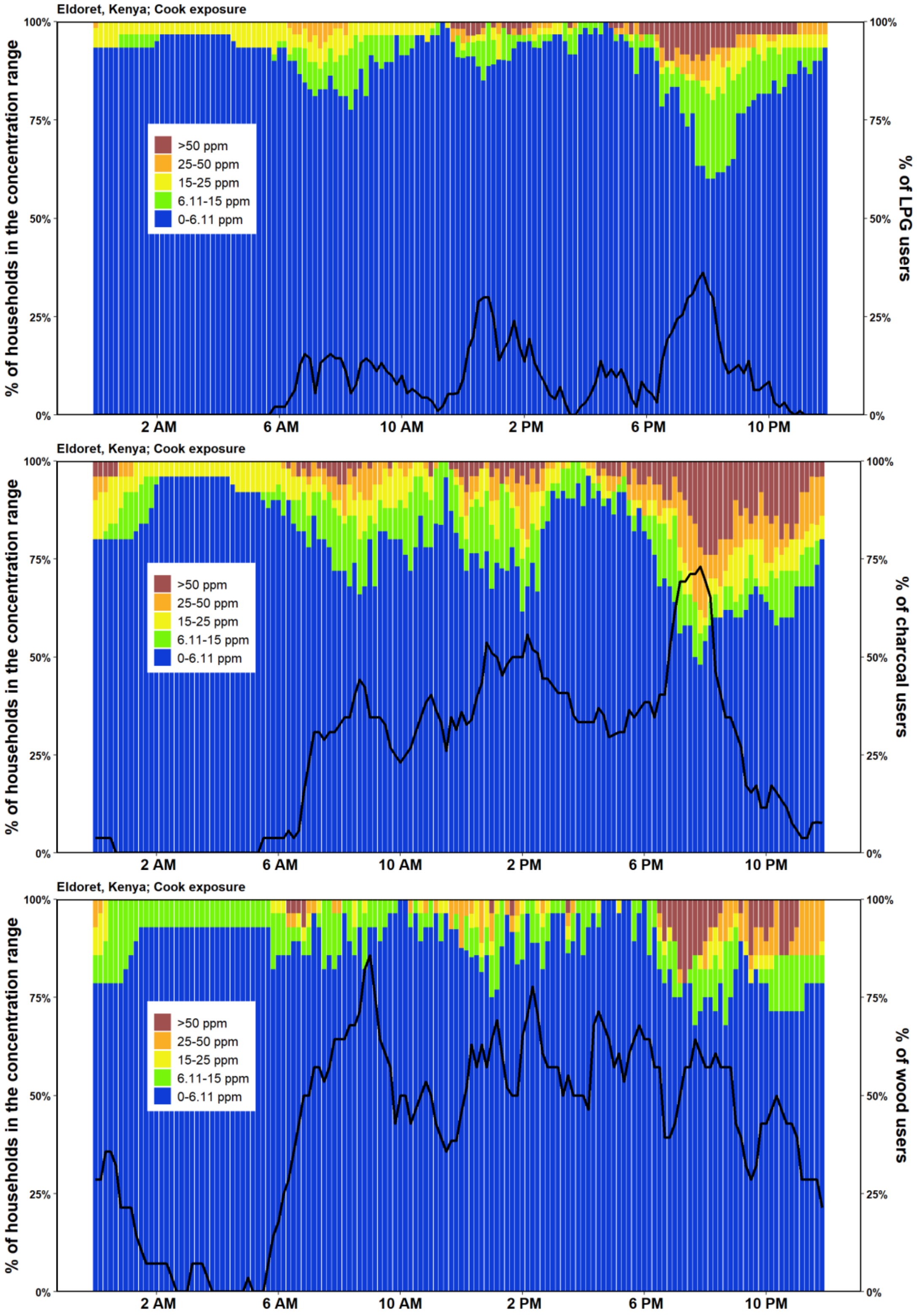

Supplementary Figure 19. Real-time $PM_{2.5}$ cook exposure measurements in Eldoret, Kenya among households primarily cooking with LPG (top), charcoal (middle) and wood (bottom).

Ambient air pollution levels

Supplementary Table 10. Ambient air pollution measurements collected in three peri-urban communities during study period

Community	Community-level average $PM_{2.5}$ concentration	Date	24-hour average $PM_{2.5}$ concentration	Village name
Mbalmayo, Cameroon	14 μg/m^3	May 31, 2019	18 μg/m^3	Newton
		May 31, 2019	11 μg/m^3	Ngalan
		May 31, 2019	8 μg/m^3	Obeck
		August 2, 2019	21 μg/m^3	Ngock
Obuasi, Ghana	31 μg/m^3	March 12, 2020	42 μg/m^3	Odumasi
		May 28, 2020	13 μg/m^3	Bogobri
		May 28, 2020	12 μg/m^3	Sam Jonah
		June 2, 2020	27 μg/m^3	Asonkore
		June 15, 2020	14 μg/m^3	Bedieso
		June 18, 2020	18 μg/m^3	Tutuka
		June 24, 2020	106 μg/m^3	Akaporiso
		June 24, 2020	31 μg/m^3	Boete
		June 28, 2020	26 μg/m^3	Odumasi
		July 5, 2020	18 μg/m^3	Kwabenakwa
Eldoret, Kenya	6 μg/m^3	September 18, 2019	2 μg/m^3	Kesses
		September 23, 2019	3 μg/m^3	Kesses
		October 8, 2019	7 μg/m^3	Kesses
		October 15, 2019	9 μg/m^3	Kesses
		November 11, 2019	5 μg/m^3	Kesses
		November 18, 2019	8 μg/m^3	Kesses
		November 25, 2019	10 μg/m^3	Kesses
		December 9, 2019	1 μg/m^3	Kesses
		December 16, 2019	6 μg/m^3	Kesses
		January 7, 2020	4 μg/m^3	Kesses
		January 14, 2020	9 μg/m^3	Kesses

Cooking hours

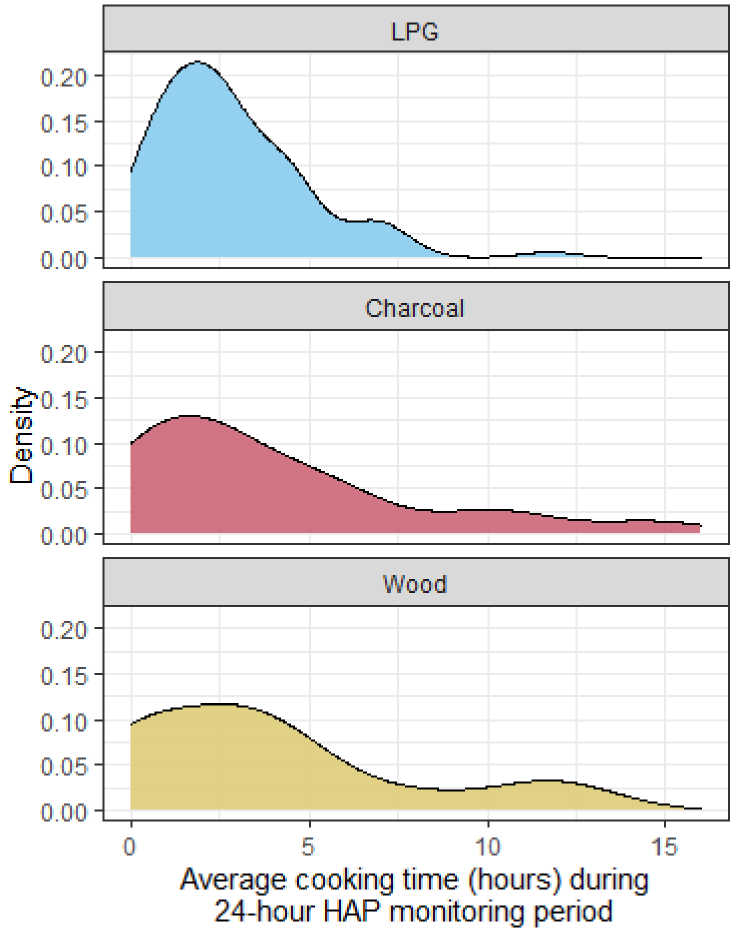

Supplementary Figure 20. Distribution of cooking time (hours) during 24-hour HAP monitoring by primary cooking fuel

Wearing compliance

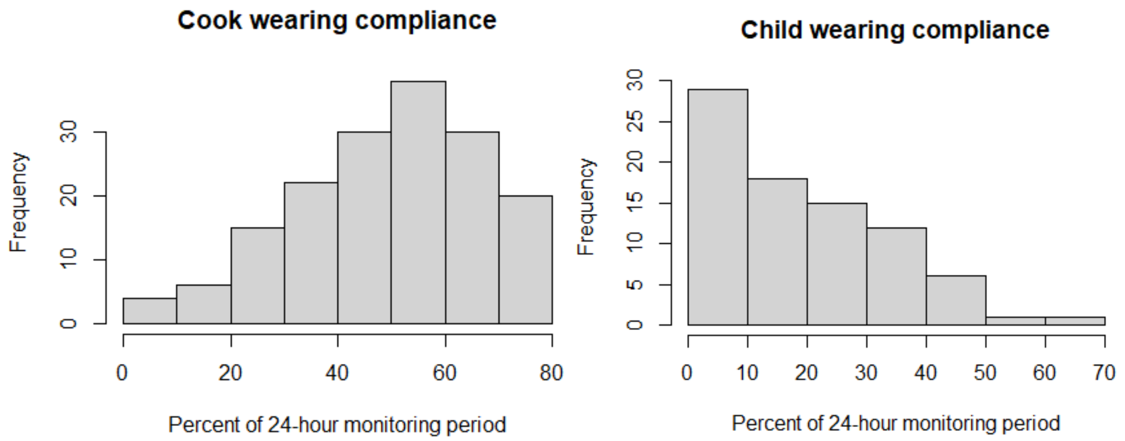

Supplementary Figure 21. MicroPEM wearing compliance among cooks and children during the 24-hour monitoring period

Supplementary Table 11. Child wearing compliance by age

Age	Average Wearing Compliance (%)
1	9.3
2	22.0
3	12.8
4	26.5
5	20.2

Sensitivity analysis: Socioeconomic characteristic comparison for sub-sample with child monitoring

Supplementary Table 12. Comparing socioeconomic characteristics of population with cook HAP measurements data to subsample with concurrent cook and child HAP measurements

	**Households in Obuasi, Ghana and Eldoret, Kenya with cook HAP exposure monitoring (N=168)**	**Households in Obuasi, Ghana and Eldoret, Kenya with cook and child HAP exposure monitoring (N=124)**	**p-value (X^2 test of independence)**
Age of household head (Mean (SD))	41.2 (12.0)	41.8 (12.6)	0.75
Marital status of cook			0.89
Married	107 (57%)	79 (51%)	
Single	48 (23%)	32 (27%)	
Cohabitating	9 (13%)	9 (15%)	
Widowed	9 (7%)	6 (9%)	
Financial security			0.80
Have enough money	51 (29%)	33 (26%)	
Not quite enough	83 (48%)	62 (49%)	
Definitely not enough	39 (23%)	31 (25%)	
Highest education level of cook			0.96
No formal education	15 (9%)	10 (8%)	
Primary	38 (23%)	30 (24%)	
Secondary/high school	80 (48%)	60 (49%)	
University	34 (20%)	23 (19%)	
Toilet in home			0.51
Yes	61 (35%)	39 (31%)	
Primary lighting fuel			0.82
Electricity	155 (90%)	111 (88%)	
Solar powered lantern/ flashlight/ oil lamp	18 (10%)	15 (12%)	
Cooking location			0.63
In home (no separate room)	5 (3%)	4 (3%)	
In home (separate room)	66 (38%)	37 (29%)	
Outside home (separate room)	43 (25%)	35 (28%)	
Veranda/porch	48 (28%)	40 (32%)	
Outside home (open air)	11 (6%)	10 (8%)	

Characteristics by occupation

Supplementary Table 13. Occupation of primary cook by community and primary cooking fuel (occupation data unavailable from Eldoret, Kenya)

Occupation	Overall (N=147)		Mbalmayo, Cameroon (N=83)		Obuasi, Ghana (N=64)	
	LPG (N=77)	Polluting (N=70)	LPG (N=43)	Polluting (N=40)	LPG (N=34)	Polluting (N=30)
Business owner	26 (34%)	27 (40%)	11 (26%)	11 (28%)	15 (44%)	16 (53%)
Homemaker	11 (14%)	16 (24%)	11 (26%)	16 (40%)	0	0
Government/business employee	16 (21%)	7 (10%)	10 (23%)	5 (12%)	6 (18%)	2 (7%)
Craftsperson/day laborer	12 (15%)	2 (3%)	5 (11%)	2 (5%)	7 (20%)	0
Farmer	0	8 (12%)	0	2 (5%)	0	6 (20%)
Unemployed	12 (15%)	10 (15%)	6 (12%)	4 (10%)	6 (18%)	6 (20%)

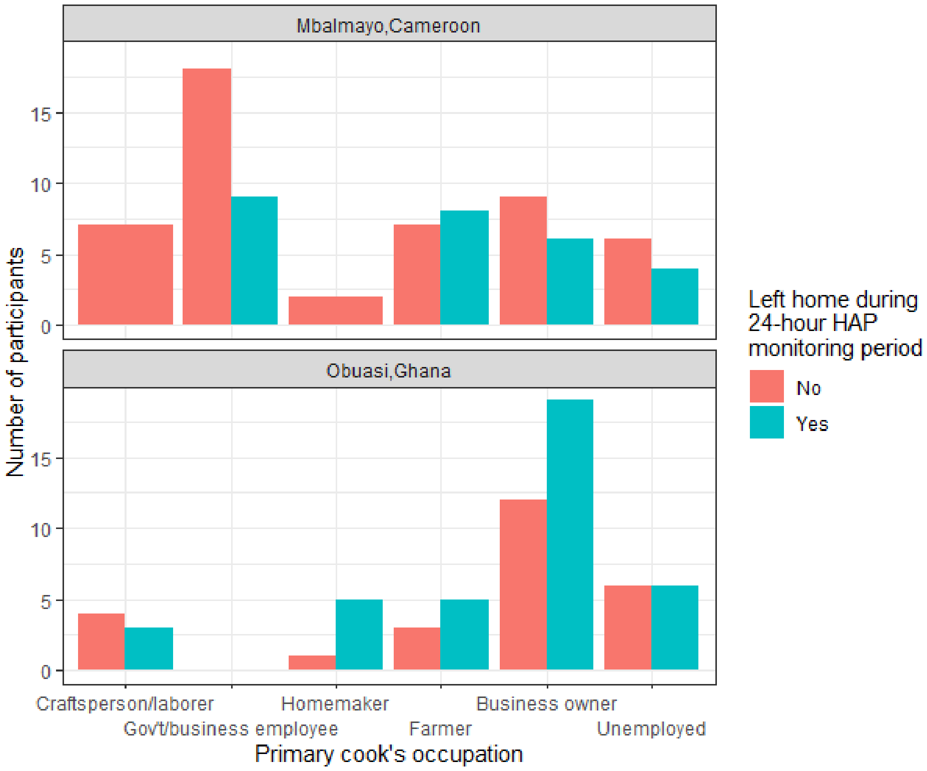

Supplementary Figure 22. Whether primary cook traveled outside their home during the 24-hour HAP monitoring period community and occupation (occupation data unavailable from Eldoret, Kenya)

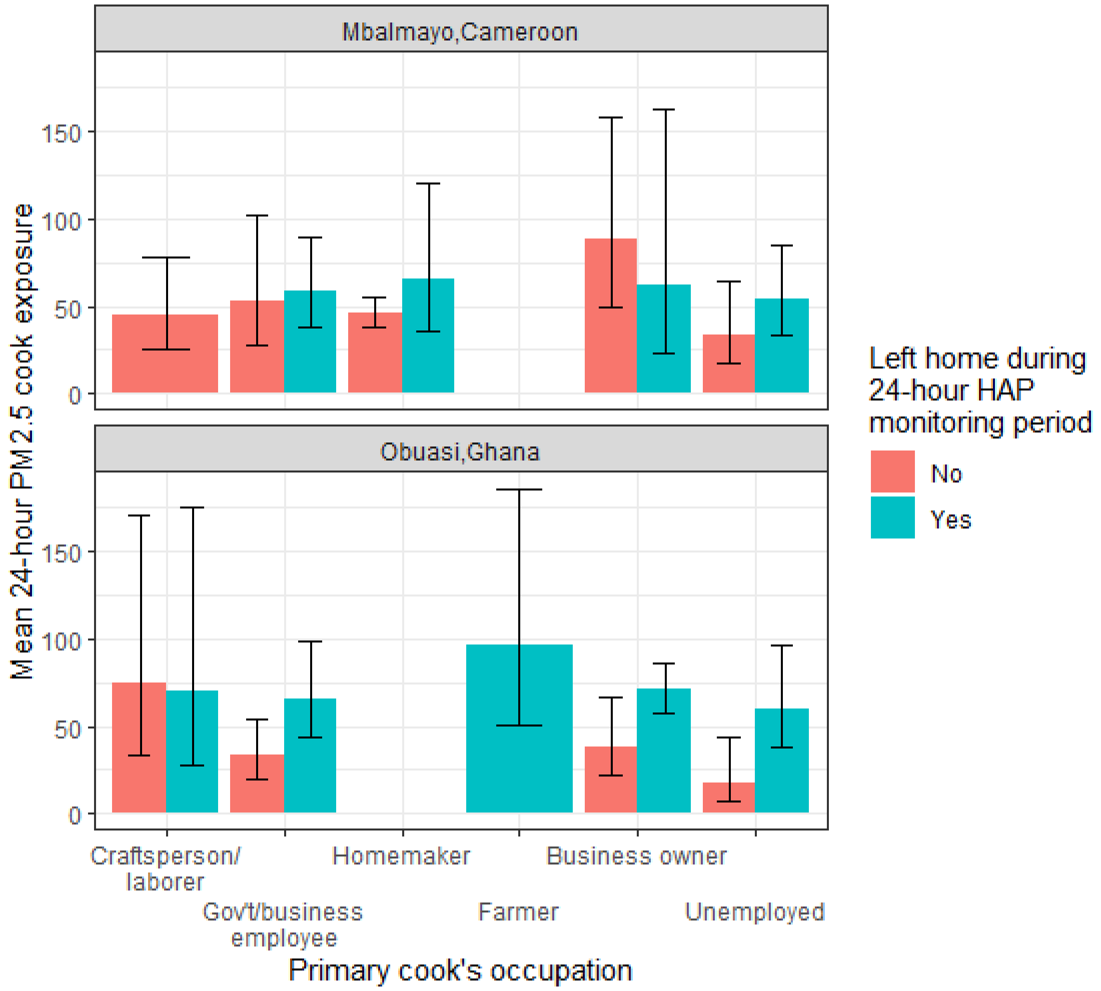

Supplementary Figure 23. Average 24-hour $PM_{2.5}$ cook exposures by whether they left their home during the 24-hour HAP monitoring period, community and occupation (occupation data unavailable from Eldoret, Kenya)

References

1 Johnson Michael A., Steenland Kyle, Piedrahita Ricardo, *et al.* Air Pollutant Exposure and Stove Use Assessment Methods for the Household Air Pollution Intervention Network (HAPIN) Trial. *Environmental Health Perspectives*; **128**: 047009.

2 Chartier R, Phillips M, Mosquin P, *et al.* A comparative study of human exposures to household air pollution from commonly used cookstoves in Sri Lanka. *Indoor Air* 2017; **27**: 147–59.

3 Bordner* A, Asante KP, Parekh M, *et al.* Validation Assessment of the RTI Micropem V3.2 as Part of Preliminary Research for the Ghana Randomised Air Pollution and Health Study (GRAPHS). *ISEE Conference Abstracts* 2014; published online Oct 20. DOI:10.1289/isee.2014.P1-180.

4 Simkovich SM, Williams KN, Pollard S, *et al.* A Systematic Review to Evaluate the Association between Clean Cooking Technologies and Time Use in Low- and Middle-Income Countries. *International Journal of Environmental Research and Public Health* 2019; **16**: 2277.

Research protocol

National Institute for Health Research CLEAN-Air (Africa) Global Health Research Group

A programme of research to support scaled adoption of liquified petroleum gas (LPG) as a clean household fuel to improve health for communities in Cameroon, Ghana and Cameroon

Partner institutions:

Moi University

Kintampo Health Research Centre

University of Ghana

Douala General Hospital

University of Liverpool

The CLEAN-Air (Africa) Global Health Research Group sponsored by the UK National Institute of Health Research

Previous research conducted by the University of Liverpool (UoL) and her partners over many years has been instrumental in highlighting the global importance of moving from solid fuel cooking technologies such as firewood and charcoal to clean fuels such as liquefied petroleum gas (LPG) to impact on the Global Burden of Disease (GBD) from Household Air Pollution (HAP) (Lim et al. 2012). The UoL work directly contributed to the development of the latest WHO Indoor Air Quality Guidelines for Household Fuel Combustion (WHO, 2014). Research is now needed to identify and overcome the challenges low and middle-income countries (LMICs) face to achieve large-scale, equitable and sustained transition to clean fuels and to demonstrate the achievable gains for health, household finances and the environment, to inform national policies.

The NIHR CLEAN-AIR(Africa) Global Health Research Group (GHRG) is a consortium of researchers from the United Kingdom (UoL) and Africa; Ghana – Kintampo Health Research Centre and the University of Ghana, Kenya – Moi University, and Cameroon – Doula General Hospital. The Group is funded by the UK National Institute for Health Research (NIHR), with UoL as the prime grant holder. The Group will provide evidence to support countries' transition to clean household energy through a program of applied research and capacity building working directly with stakeholders in Ghana, Kenya and Cameroon. The three countries have recently made ambitious commitments to scaling-up household access to LPG as a clean household energy by a significant proportion of the population. The Group will engage with health, energy and development sectors in all three countries.

Liquified Petroleum Gas (LPG) to address the burden of disease from household air pollution in Sub-Saharan Africa

Approximately 700 million people are dependent on the use of solid fuel (predominantly firewood) for cooking in Sub-Saharan Africa, a figure that continues to rise despite decreases in other world regions. It is estimated that almost 900 million people will be reliant on biomass fuel by 2020, as efforts to expand access to modern cleaner energy (electricity and gas) are far outpaced by population growth (Lambe et al., 2015). The negative impacts of traditional solid fuel reliance are well established (Smith et al., 2014). Unsustainable harvesting of fuel wood and associated deforestation are a major issue in many LMICs (Subedi et al., 2014) with 70% of deforestation in Africa attributed to wood fuel demand and predicted to account for 83% by 2030. The negative impacts on climate and health are also well established, with household air pollution (HAP) resulting from burning biomass estimated to contribute to 25% of global black carbon, the most important influence on climate change after carbon dioxide (CO_2) (Bailis et al., 2005). In 2016 in Sub-Saharan Africa, exposure to HAP from cooking with solid fuel was estimated to result in 520,000 premature deaths (6.8% of all deaths) and almost 23 million disability-adjusted-life-years (DALYS; 4.6% of total), from ischaemic heart disease, stroke, lung cancer and chronic obstructive pulmonary disease in adults and pneumonia in children (IHME, 2016). The economic costs of solid fuel reliance in Sub-Saharan Africa are also substantive and estimated at US$37 billion per year (2.8% of GDP), largely due to lost productivity taken up with time intensive fuel gathering and cooking processes (approx. US$30 billion) (Lambe et al., 2015). The associated health and economic burdens are largely born by women and girls who are chiefly responsible for cooking and fuel gathering following traditional gender roles, making reliance on solid fuels a major source of gender inequality (Austin and Mejia, 2017).

In 2014, the WHO published Indoor Air Quality Guidelines for Household Fuel Combustion to address what they called "the greatest environmental health risk in the world today" (WHO, 2014). The

Guidelines were intended to support public health policy makers and stakeholders in the energy sector to understand the best approaches for reducing HAP. One key recommendation from the guidelines was that clean fuels should be prioritised to meet target emission levels to protect health and that "governments and their implementing partners should develop strategies to accelerate efforts to meet (these lower emission rates)". Making clean energy solutions (including gas, electricity and biogas) more widely available, especially amongst the world's poorest people, is also key to achieving Sustainable Development Goal 7 (SDG 7) to "ensure universal access to affordable, reliable, sustainable and modern energy for all" by 2030 (WHO, 2016).

Liquified Petroleum Gas (LPG) or bottled gas is widely available across many geographical regions of Sub-Saharan Africa, although under used, and is an efficient, portable and safe cooking fuel with the potential to deliver benefits for health, climate, the environment and development (Bruce et al., 2017). Accordingly, a number of Sub-Saharan African governments have made it a priority to provide a majority of their populations with LPG with a view to addressing air pollution, preserving forests and strengthening economic development (Van Leeuwen et al., 2017, Bruce et al., 2017).

Choice of focus countries for CLEAN-Air (Africa)

Three Sub-Saharan African countries are the focus for the research and capacity building activities under CLEAN-Air (Africa); Cameroon, Ghana and Kenya. All three are implementing policies to scale adoption of LPG as a clean household energy to address the negative impacts on health, the environment, deforestation and energy security of domestic reliance on solid fuels for cooking and heating.

Kenya is currently implementing policies to scale adoption of LPG as a clean household energy to address the negative impacts on health, the environment, deforestation and energy security of domestic reliance on solid fuels for cooking and heating.

In Kenya, 76% of the population (35m) relies on solid fuels and kerosene for cooking and heating. Associated household air pollution (HAP) accounted for 727,689 (4.4% total) DALYs and 16,556 premature deaths (6.1% of all deaths) in 2016. LPG expansion is a priority in the country with a stated goal to double national LPG consumption by 2030. In 2017, the government had established a program (the "Mwananchi Gas project") to supply over 4 million subsidized 6kg cylinders with on top burners to poor households over 4 years. The ultimate goal was to reach 70% LPG penetration rates. However, the Mwananchi Gas project has being paused[1] but other important legal and regulatory reforms are taking place to boost the uptake of LPG and reduce illegal LPG refilling activities.[2] CLEAN-Air(Africa) will work closely with the Ministry of Petroleum & Mining and other local stakeholders to provide an evidence base for sustained use of LPG by communities. The Group will also evaluate innovative consumer financing initiatives (e.g. pay-as-you-go use of LPG with smart meter technology and microfinance).

In **Cameroon**, where approximately 70% of the population use solid fuel for cooking (more than 90% in rural communities) (DHS, 2011), the government has a target to increase LPG adoption from approximately 20% of the population to 58% (18 million people) by 2030. To this end, with support from the Global LPG Partnership (GLPGP)[3], they have developed an LPG for Clean Cooking

---

[1] https://www.theeastafrican.co.ke/news/ea/Kenya-cooking-gas-project-for-the-poor-burnt-up-by-graft-/4552908-4815272-cpq40bz/index.html

[2] http://www.xinhuanet.com/english/2018-07/27/c_137350460.htm

[3] GLPGP is a non-profit public-private partnership established in 2012 as a high-impact initiative under Sustainable Energy for All that help developing countries transition large populations rapidly to LPG for cooking on a commercially sustainable basis. See www.glpgp.org

Masterplan to inform strategies to scale adoption through improved market regulation, infrastructure, supply and access, announced in 2016 (SE4All, 2017). One important aspect of facilitating the widespread transition from solid fuel to LPG for cooking is to understand how best to encourage and support households to both adopt LPG and use it exclusively in a sustained way.

The LPG Adoption in Cameroon Evaluation (LACE) studies were launched by our group at UoL in 2016 to (i) identify potential enablers and barriers to adoption and sustained use of LPG at household level and (ii) test interventions to support communities to make the transition to cleaner fuel. The independent studies were conducted after publication of the LPG Masterplan to provide evidence to support scaled transition from solid fuel to LPG from a community perspective (Bruce et al 2018). The methods adopted and interventions assessed for the LACE studies formed the basis for the present extended program of research to further inform policies to scale up LPG transition as a clean household energy in Cameroon and beyond for CLEAN-Air (Africa).

In **Ghana**, approximately 70% of households rely on solid fuels and biomass for cooking. Household air pollution (HAP) arising from their use is responsible for 16,600 premature deaths and the loss of 502,000 disability adjusted life-years annually (GSS, 2012, 2014b; Inkoom & Crentsil, 2015). These fuels also contribute substantially to ambient air pollution in the country (Fullerton, Bruce, & Gordon, 2008; GSS, 2014a; WHO, 2011). Thus, there is a need to promote low emission 'clean' fuels such as Liquefied Petroleum Gas (LPG) (MacCarty, Still, & Ogle, 2010; WHO, 2011).

Ghana became the first country to promulgate a Sustainable Energy for All Action Plan, as called for by the United Nation's Sustainable Energy for All (SEforALL) program In June 2012 (ENERGIA, 2015). The plan emphasized the importance of promoting LPG as clean energy for cooking, amongst other strategies and set up an initial goal to expand LPG access to 50% of Ghana's population by 2020[4] (now moved to 2030). To effectively scale safe adoption of LPG as a clean household energy, the Ghana SEforALL plan also called for a national change in LPG infrastructure to the LPG cylinder recirculation model (CRM), whereby LPG marketers are responsible for ownership and safety of LPG cylinders, (as opposed to the current end user ownership), as a model for LPG distribution in the country (Energy-Commission, 2012). The National Petroleum Authority has been mandated in 2017 by the President of Ghana to implement transition to the CRM and piloting of the new distribution model is expected to start in the first quarter of 2019[5].

Over-arching research goal

The broad remit of the Group is to develop a programme of applied research, which will support population transition from polluting solid fuels and kerosene for household energy to cleaner fuels, to address the substantial burden of disease from non-communicable diseases (NCDs) related to exposure to HAP.

Goal: to ***inform national strategies to support more equitable uptake*** of clean fuel across the population by (i) identifying enabling/ inhibiting factors to use of LPG as well as potential solutions and (ii), developing, testing and evaluating interventions to facilitate equitable adoption/ use of LPG (including consumer finance solutions).

[4] https://www.se4all-africa.org/fileadmin/uploads/se4all/Documents/News___Partners_Docs/GHANA_SE4ALL_Newsletter_Oct-Dec__16.pdf
[5] https://www.myjoyonline.com/news/2018/November-23rd/npa-to-pilot-cylinder-recirculation-model-in-kumasi.php

Aims and objectives

CLEAN-Air(Africa) will conduct a comprehensive program of in peri-urban and rural communities of the three partner countries and capacity building activities.

We will work with study communities to understand how households currently manage domestic energy and explore potential barriers and enablers to adoption and sustained use of LPG as a clean household energy. We will also work with a sub-sample of households to collect HAP data and record positive impacts on pollution and health arising from the reduction in exposure to fine particulate matter ($PM_{2.5}$) and carbon monoxide (CO) derived from cooking with LPG.

There are three main aims related to Part 1:

Aim 1.1: To identify (i) current fuel use patterns and cooking activities, (ii) cross-sectional relationships between fuels used, HAP data and health outcomes, (iii) any association between household characteristics and extent of LPG use and (iv) household perceptions of LPG as a cooking fuel.

Research Questions under Aim 1.1:

- How do households currently use fuels for cooking and heating?
- How do households view LPG as a potential clean cooking fuel in contrast to other fuel options?
- What factors might help households adopt LPG for the first time (or use LPG in a more sustained way if already using the fuel)?
- How does differential fuel use affect acute respiratory health symptoms, blood pressure and the experience of burns and scalds in women and children?

Objectives under Aim 1.1:

To conduct:

1. A community 'rapid' survey in representative samples of peri-urban and rural households using previously validated, interviewer-led questionnaires.

2. In-depth household surveys (including questions on health and blood pressure measurement) for households sampled according to reported use of cooking fuels (solid fuel vs LPG) in the community survey.

3. Semi-structured interviews (SSIs) and Focus Group Discussion (FGDs) with selected households participating in the in-depth household survey from both fuel using groups.

Aim 1.2: To summarise the impact of different fuel use on concentrations of (and exposure to) household air pollution in women and children.

Research Questions under Aim 1.2:

- How does differential fuel use (solid fuels vs LPG) affect concentrations of (and exposure to) household air pollution?
- How are concentrations of and exposure to household air pollution related to use of traditional and LPG stoves for cooking?

Objectives under Aim 1.2:

1. In samples of (i) solid fuel and (ii) LPG using households, to measure $PM_{2.5}$ in kitchens (concentrations) and for women and children (exposures) using microPEM technology.

2. In the same households, to record cooking events over a 7-day period through temperature monitoring of all stoves used for cooking through stove use monitors (SUMs).

Aim 1.3: To explore socio-cultural and structural influences over household cooking energy options and decision-making to better understand how these impact on potential adoption, sustained and exclusive use of LPG as a clean fuel and identify priorities for future policy and programme development.

Research Questions under Aim 1.3:

- What factors influence household decision-making over (i) initial adoption of LPG and/or (ii) sustained LPG use and/or (iii) more exclusive use of LPG ?
- What are the wider community-level and structural factors influencing fuel options available to households from the perspective of users/the community?
- To what extent do households consider LPG to meet their fuel and cooking needs and in which ways does LPG fall short?
- What are the circumstances in which LPG fails to meet households' fuel and cooking needs and how can these be addressed?
- What are the opportunities for clean fuel stacking and what fuel and stove combinations might these comprise?
- How does cooking with LPG sit within the household's cooking energy system, the household energy system (including lighting, space heating) and the local energy system?
- What measures can households take to reduce exposure to household emissions and protect family members most at risk (including those with pre-existing health conditions, pregnant women and young children) and how might these measure be best communicated to households?

Methods

Study setting

- **Kenya**: Part 1 will be conducted in peri-urban communities in Uasin Gishu County (e.g. Kesses) that have a fairly well-established LPG market with access to LPG retail points – this is important to sample households with some LPG use or the potential to transition to LPG.
- **Ghana**: Part 1 will be conducted in peri-urban communities near Kintampo or Kumasi that have a fairly well-established LPG market with access to LPG retail points.
- **Cameroon**: Part 1 will be conducted in two sites of the Center Region of Cameroon, (i) Mbalmayo (a town with a fairly well-established LPG market, with households using both biomass and LPG fuels) and (ii) Akom nyada, a rural area in proximity of Mbalmayo, with households primarily relying on freely collected biomass fuel. The latter community will take part in the evaluation of lung function through spirometry.

Community survey (all countries)

Selected communities will initially receive a period of engagement and sensitisation whereby, after consultation with the research team, community leads communicate to their communities the aims of the research, what it will involve and how it might impact on households. This will take place over a 1 to 2-week period.

Following sensitisation, households within the selected communities will be enumerated and a random sample of up to 2,000 households will be selected for a rapid community survey. The short survey (approximately 20 minutes) will collect information about household demographics, socio-economic status and current domestic fuel use and will provide a sampling frame for more in-depth household surveys to understand household factors associated with different fuel use. The survey will ask whether respondents would be willing to take part in further research.

The surveys will be conducted by trained fieldworkers at times during the day when either the main cook or the head of household are available to answer the questionnaire. In the event that a sampled household is empty (or there is no one eligible to complete the questionnaire) fieldworkers will move onto the next household in the sampled list and will return to the missed household at a different time. The survey will be administered using mobile phones and bespoke data collection software - Mobenzi Researcher (https://www.mobenzi.com/) (Ghana will be using REDCAP). Mobenzi Researcher is a practical and secure data collection software system whereby data entered onto the mobile phone via the Mobenzi App are automatically uploaded via the phones sim card (or via wifi in areas where there is no reception) to encrypted storage on the Mobenzi cloud. The system allows for contemporary quality control of data and is entirely secure (all data are encrypted at source).

Based on responses to the rapid community survey, consenting households will be randomly selected from two fuel using groups: (i) primary and exclusive use of LPG as a cooking fuel and (ii) exclusive use of solid fuel/ kerosene as a cooking fuel. Approximately 300 households will be randomly selected from each group (total 600) to obtain a sample of 200 completed questionnaires per group (total 400).

The household surveys will be completed by the primary cook of each household. Appointments will be made based on the contact details provided by respondents during the community survey. Trained fieldworkers will administer the questionnaires using mobile phones and the Mobenzi Researcher platform as for the community survey.

The questionnaires will take approximately 1 hour to complete and will collect information on (i) household demographics, (ii) details on fuels used for cooking, heating and lighting, (iii) cooking activities and time spent for cooking, (iv) general health, acute health symptoms and burns and (v) perceptions of LPG as a clean, efficient and safe fuel and willingness to pay for LPG.

As part of the survey process, blood pressure and heart rate measurements will be taken from all consenting participants. These will involve three independent readings of systolic and diastolic blood pressure and heart rate being taken by trained fieldworkers using electronic Omron M7 Intelli IT 360 upper arm blood pressure monitors. The three measurements will be taken as part of the health questions administered during the in-depth household survey following standard procedures recommended by the international society for hypertension.

Household air pollution measurement and stove use monitoring (all countries)

A random sub sample of 40 or more households from each fuel use group (primary or exclusive use of LPG and exclusive use of solid fuel/ kerosene for cooking with at least one child under 5 years), total of 80 or more households, will be selected from participants for the in-depth household survey to take part in detailed measurement of household air pollution and monitoring of their stove usage.

a) Particulate Matter ($PM_{2.5}$) measurement

Measurement of respirable particulate matter ($PM_{2.5}$) will be carried out using the Research Triangle Institutes (RTI) MircoPEM technology. The MicroPEM is designed to be a low-burden, lightweight (<

240g) personal exposure monitor, utilising an on-board micro nephelometer to measure real-time $PM_{2.5}$ concentrations. Activity level and protocol wearing compliance are also monitored simultaneously via an on-board accelerometer. Unlike many other lightweight samplers, the MicroPEM actively aerodynamically sizes PM to better relate personal exposures to respiratory deposition zones for $PM_{2.5}$ (deep lung or thoracic, respectively).

Kitchen concentrations of $PM_{2.5}$ will be measured by placing a MicroPEM approximately one metre away from the main source of combustion (stove) at a height of approx. one metre. Personal exposure to $PM_{2.5}$ will be measured for the primary cook and designated child (under 5 years) by placing a MicroPEM in a bespoke apron with a centrally located pocket at the front within the respirable zone (Figure 1). All MicroPEM deployments will last 24-48 hours (reflecting at least a full day's domestic energy use).

MicroPEM filters will be weighed before and after sampling at RTI International's main campus in the US. After deployment of the MicroPEMs fieldworkers will download data and calibrate the units before redeployment in another household. The fieldwork coordinator will regularly upload MicroPEM data files to RTI International. RTI will process these data using a proprietary SAS script to validate the real-time data and flag data files with parameters outside of predetermined acceptable ranges. MicroPEM parameters that will be inspected include PM concentration, temperature, relative humidity, flow rate, accelerometer, and inlet and orifice differential pressures. Flagged data files will be manually inspected for potential hardware malfunctions or improper settings. These files will be corrected when possible and fieldworkers will be notified of any hardware malfunctions.

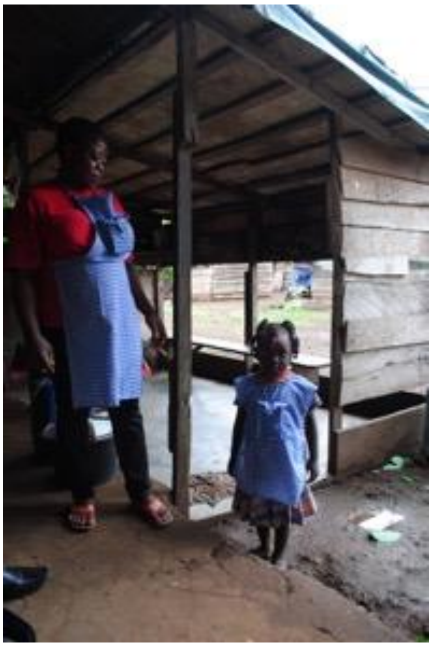

Figure 1: Deployment of MicroPEM for women and child

Ambient levels of $PM_{2.5}$ will be measured by locating a secured MicroPEM in a central pre-designated location (through discussion with community leaders) within each community setting. Four 48-hr measurements will be taken at different times during the 4-week assessment period.

b) Carbon monoxide (CO) measurements

CO will be measured in tandem with $PM_{2.5}$ as described above, using LASCAR Easy Log EL-USB-CO electrochemical monitors for both area and personal assessment. The EL-USB-CO units are small, lightweight tubes that will be co-located with the MicroPEMs during the 24-48 hour recording period. Sampling intervals will be set up to match those of the MicroPEM sampling. Instruments will be calibrated by the supplier (Thermosense) before deployment. Data will be downloaded by USB at the end of the measurement period and checked for quality.

c) Stove use monitoring (SUMs)

Homes receiving measurements of household air pollution ($PM_{2.5}$ and CO) will also have all stoves used within the household monitored to record cooking events over a 7-day period (including the 24-48-hour HAP recording window). Stove use will be measured with Geocene Dots, which are temperature loggers designed for monitoring cookstove usage over prolonged periods of time in field conditions. The process involves attaching geotagged thermocouples to the stoves (e.g. open fire, LPG burner etc) and recording regularly sampled temperature readings (same sampling periods as for HAP) which can be used to work out the location and frequency of cooking events. The data are remotely uploaded onto a mobile phone App using a Bluetooth receiver.

Data Management and Analysis

Analysis of quantitative and qualitative data will be carried out using standard methods with STATA or SPSS, and NVivo qualitative software respectively.

Quantitative analysis of cross-sectional data:

Data collected from cross-sectional community 'rapid' surveys will be analysed using descriptive statistics to summarise household fuel use for cooking, lighting and other uses. To explore the association between household characteristics and LPG adoption and sustained/ exclusive use, cross-sectional comparisons will be made using appropriate hypothesis testing (e.g Chi-squared tests for categorical comparisons and independent t-tests for continuous data). Unconditional logistic regression will be used to summarize the associations between household factors (e.g. household composition, assets, finances, socio-economic status) and LPG use (any use and primary/ exclusive use). Comparison of household and individual characteristics between fuel using groups from the household surveys (LPG primary/ exclusive use vs biomass exclusive use) will be made using appropriate hypothesis testing (e.g. independent t-tests for continuous variables like blood pressure and lung function (FEV1, FVC, FEV1/FVC ratio) and chi-squared tests for categorical variables like income categories).

Quantitative methods – Household Air Pollution measurements:

The impact of fuel use (comparing households that use LPG as primary fuel with those that use biomass exclusively) on $PM_{2.5}$ and CO (ambient, concentrations and personal exposure) will be summarized cross-sectionally by comparing geometric means from natural log transformed measurements. T-tests will be used to compare geometric means between the groups. Multivariable linear regression will be used to summarise differences in levels of $PM_{2.5}$ between (i) LPG users vs non-LPG users and (ii) exclusive LPG users vs non-exclusive LPG users adjusting for potential confounders (e.g. demographic characteristics, environmental tobacco smoke, etc.).
Potential impacts on health associated with reductions in HAP from LPG use (mixed use and exclusive) will be modelled using published integrated exposure-response (IER) curves for $PM_{2.5}$ and ischaemic heart disease, stroke, lung cancer and chronic obstructive pulmonary disease (COPD) (8).

Quantitative methods – Stove Use Monitors (SUMS):

The SUMS data will be analysed within households selected for household air pollution measurement ($PM_{2.5}$ and CO), taken over a 7-day period. The number of cooking events will be assessed and the duration of cooking (each event, per day and per week) according to temperature fluctuations of the LPG and traditional stoves will be assessed. Differences in cooking events and times will be summarised as averages between the LPG and biomass using homes and compared using independent t-tests. Household air pollution measurements taken ($PM_{2.5}$ concentrations and exposures) will be correlated with cooking patterns (frequency and duration) as measured by the SUMs.

Quantitative methods – Sample Size Calculations:

As described above, rapid surveys will be conducted of randomly selected households (n=2000) to provide the sampling frames for the evaluation work for CLEAN-Air(Africa). Equal numbers of LPG and solid fuel users will be randomly selected based on responses to the rapid survey around current fuel use and household demography. Eligible homes will be those with families including at least one child under 5 years of age. The numbers of homes are considered under sample size, below.

Sample size requirements

Two parameters have been used to determine the required sample size:

1. Survey-based household information will be assessed quantitatively. For the purpose of this calculation, aspects of fuel use for cooking are considered key, for which precision of estimates for important aspects such as % stacking LPG with solid fuels, and % of all cooking carried out with LPG, will determine sample size. Calculations for characteristics with a prevalence of 25% and 35% are shown in Table 1 below.

Table 1: Sample size calculation by prevalence of fuel use

Prevalence of characteristic	Precision (at 95% level)	Total sample size required
25%	+/- 5%	290
35%	+/- 5%	350

Comparisons between fuel use groups (LPG and solid fuels) will be made for key characteristics including demography (e.g. SES, income) and health (e.g. acute respiratory health and blood pressure). To detect a relative risk of 2 (based on previous work in Cameroon (Pope et al; Ecohealth 2018; 15: 729-743) with 95% confidence and 80% power, 163 households in each fuel use group will be required.

Based on these calculations, and allowing for around 10% refusals and lost information, a total of approximately 400 homes would be selected for survey-based data collection.

2. Demonstration (in cross-sectional baseline assessment) of differences (between predominant solid fuel and predominant LPG users) in ambient kitchen and personal exposure to $PM_{2.5}$ of approximately 75%: For $PM_{2.5}$, we assume (based on work from a modelling paper of $PM_{2.5}$ exposure reductions from LPG cookstove interventions) a mean exposure of women in solid fuel-using homes of 270 µg/m^3 (Steenland et al. 2018) and SD of 250 µg/m^3, and a mean exposure of 70

(SD = 50) μg/m^3 or lower, for women in clean fuel homes, with significance of 5% and power of 80%. This yields a sample size of 26 per group; allowing for around 25% refusals and lost data, a total of 35 per group (combined total 70) will be required. These calculations will be sufficient for demonstrating similar percentage differences in CO. While it might be anticipated that predominant LPG users may see larger reductions in HAP, the sample size has been designed based on exposure reductions found in previous cookstove interventions. Higher exposures related to LPG may be a result of stacking, emissions from neighbours, and other ambient sources, with reasonable precision.

Using an exposure-response relationship between systolic blood pressure (SBP) and $PM_{2.5}$ (Figure 1; copied from Baumgartner et al. 2011), the expected cross-sectional difference in systolic blood pressure between wood and LPG users can be estimated. While the relationship in Figure 1 was mapped for a different demographic (138 women in rural China >age 50), it is noted that the relationship between $PM_{2.5}$ and blood pressure has held in other settings, with several studies showing a difference in SBP between solid fuels and clean fuels of 1.5-7 mmHg (Arku et al 2018).

With an estimated $PM_{2.5}$ exposure of 270 ug/m^3 for wood users and 70 ug/m^3 for LPG users, a difference in SBP of 6 mmHg is estimated between wood and LPG users (Figure 1; 131 vs 125 mmHg, respectively). The estimated sample size needed to achieve 80% power, 5% significance, with a standard deviation of 16 mmHg for SBP in Ghana (Arku et al 2018) and accounting for ~20% estimated loss, is 140 in each group (total: 280).

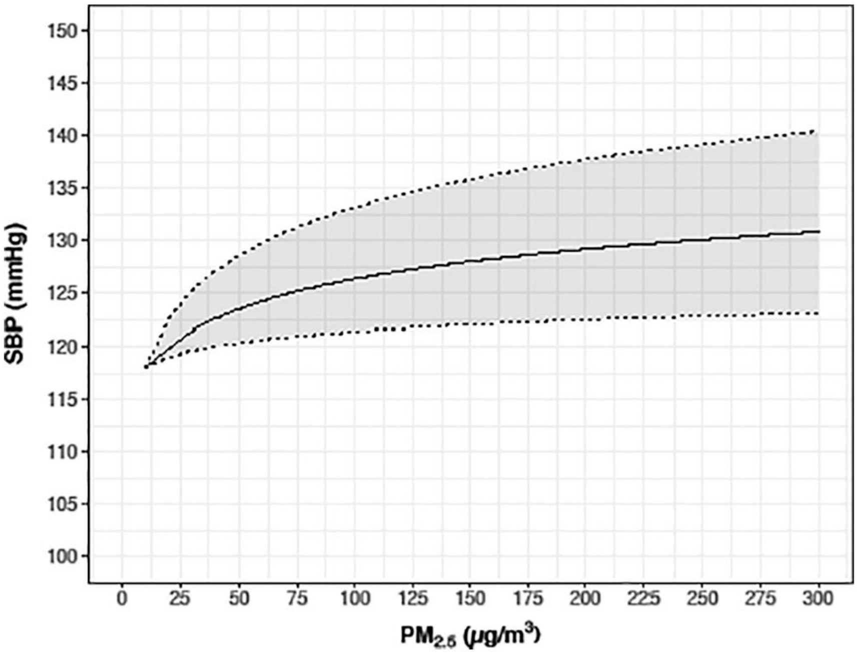

Figure 1. Exposure-response relationship for systolic blood pressure in women > 50 years old

Summary of sample size requirements

In summary, a sample size of around 400 is required for adequate precision of quantitatively assessed characteristics. For measures of air pollution, random sub- samples of around 35 per group will be needed.

References for sample size calculations:

Arku RE, Ezzati M, Baumgartner J, Fink G, Zhou B, Hystad P, Brauer M. Elevated Blood Pressure and Household Solid Fuel Use in Premenopausal Women: Analysis of 12 Demographic and Health Surveys (DHS) from 10 Countries. Environmental Research 2018, 160, 499–505. https://doi.org/10.1016/j.envres.2017.10.026.

Baumgartner J, Schauer, JJ, Ezzati M, Lu L, Cheng C, Patz JA, Bautista LE. Indoor Air Pollution and Blood Pressure in Adult Women Living in Rural China. Environmental Health Perspectives 2011, 119 (10), 1390–1395.

Pope D, Bruce N, Higgerson J, Hyseni L, Stanistreet D, MBatchou B, Puzzolo E. Household determinants of Liquified Petroleum Gas (LPG) as a Cooking Fuel in SW Cameroon. Ecohealth 2018; 15(4): 729-743.

Ofori SN, Fobil JN, Odia OJ. Household biomass fuel use, blood pressure and carotid intima media thickness; a cross-sectional study of rural dwelling women in Southern Nigeria. Environmental Pollution 2018; 242; 390-397.

Steenland K, Pillarisetti A, Kirby M, Peel J, Clark M, Checkley W, Chang H. H, Clasen T. Modeling the Potential Health Benefits of Lower Household Air Pollution after a Hypothetical Liquified Petroleum Gas (LPG) Cookstove Intervention. Environment International 2018, 111, 71–79. https://doi.org/10.1016/j.envint.2017.11.018.

Ethics approvals

In each of the countries, ethical approval will be obtained from the relevant Research Ethics Committees (RECs). Subsequently, we will seek approval from the University of Liverpool REC.

Cameroon: Ethical approval was obtained by the '*Comite Regional D'Ethique de la Recherche pour la Sante Humaine du Centr'e* (Cameroon National Ethics Committee) in October 2018 and from the University of Liverpool REC in January 2019.

Kenya: An application for obtaining ethical approval was submitted to the Kenya Ethics Committee in January 2019. Once local approval is granted, a request to obtain approval from UoL REC will be processed.

Ghana: An application for obtaining ethical approval was submitted to the Ghana Ethics Committee in February 2019. Once local approval is granted, a request to obtain approval from UoL REC will be processed.

Gaining informed consent and withdrawal

All the aspects of the study will be explained to the participants and they will be given an information sheet for each study component. For those consenting to take part in the study, an informed written consent will be obtained from all the participants. As stated in the informed consent form, participants will be free to withdraw from the study at any time without giving any reason and without their rights being affected. Participants will be able to ask for access to the information they provide, including the destruction of that information if they wish at any time prior to anonymization. Following anonymization they will no longer be able to request access to or withdrawal of the information I provide.

Participants found to have high blood pressure (hypertension) during household surveys will be advised to seek medical advice at the local health centre.

Data anonymization, processing, and storage

The University of Liverpool processes personal data as part of its research and teaching activities in accordance with the lawful basis of ‘public task’, and in accordance with the University’s purpose of “advancing education, learning and research for the public benefit”. Under UK data protection legislation, the University acts as the Data Controller for personal data collected as part of the University’s research. In each country, the principal investigator of each country will as the Data Processor for this study. Participants’ data will be stored on the Universities secure servers (in Liverpool, via the University of Liverpool’s Managed Windows Service). Transcription and anonymisation of data will only be performed by the research team and confidentiality will be maintained throughout this and all data handling and storage processes. The research team’s computers are password protected and encrypted. We will transfer the quantitative data into a database and make participants’ data anonymous by removing every name and replacing it with a code, and also by removing and replacing any other features that might be identifiable. The qualitative data (interviews and focus group discussions) will be transcribed, and any direct identifiers will be removed and substituted for reference numbers or other indirect indicators. At the end of the study, any paper copies of the data will be shredded and securely destroyed. Electronic data files will be destroyed after 10 years.

References

AUSTIN, K. & MEJIA, M. 2017. Household air pollution as a silent killer: women's status and solid fuel use in developing nations *Population and Environment,* DOI: 10.1007/s11111-017-0269-z.

BAILIS, R., EZZATI, M. & KAMMEN, D. 2005. Mortality and Greenhouse Gas Impacts of Biomass and Petroleum Energy Futures in Africa. *Science,* 308**,** 98-103.

BRUCE, N., AUNAN, K. & REHFUESS, E. 2017. Liquified Petroleum Gas as a clean cooking fuel for developing countries: Implications for Climate, Forests and Affordability. KfW Development Bank 2017; Materials on Development Financing No. 7.

GUEST, G., BUNCE, A. & JOHNSON, L. 2006. How many interviews are enough? An experiment with data saturation and variability. *Field methods,* 18**,** 59-82.

IHME. 2016. *Institute for Health Metrics and Evaluation (IHME). GBD Compare Data Visualization. Seattle, WA: IHME, University of Washington, 2016. Available from http:// vizhub.healthdata.org/gbd-compare. (Accessed 13-11-17)*.

Harper, D. (2002). Talking about pictures: A case for photo elicitation, *Visual Studies*, 17(1), 13–26.

LIM, S., VOS, T., FLAXMAN, A., DANAEI, G., SHIBUYA, K., ADAIR-ROHANI, H. & AL., E. 2012. A comparative risk assessment of burden of disease and injury attributable to 67 risk factors and risk factor clusters in 21 regions, 1990–2010: a systematic analysis for the Global Burden of Disease Study 2010. *Lancet* 380**,** 2224–60.

LAMBE, F., JÜRISOO, M., WANJIRU, H. & SENYAGAWA, J. 2015. Bringing clean, safe, affordable cooking energy to households across Africa: an agenda for action *Prepared by the Stockholm Environment Institute, Stockholm and Nairobi, for the New Climate Economy. Available at: http://newclimateeconomy.report/misc/working-papers*.

SMITH, K., BRUCE, N., BALAKRISHNAN, K., ADAIR-ROHANI, H., BALMES, J., CHAFE, Z., DHERANI, M., HOSGOOD, H., MEHTA, S., POPE, D. & REHFUESS, E. 2014. Millions dead: how do we know and what does it mean? Methods used in the comparative risk assessment of household air pollution. *Annu Rev Public Health,* 35**,** 185-206.

SUBEDI, M., MATHEWS, R., POGSON, M., ABEGAZ, A., BALANA, B. & ETAL. 2014. Can biogas digesters help to reduce deforestation in Africa? . *Biomass and Bioenergy,* 70**,** 87-98.

VAN LEEUWEN, R., EVANS, A. & HYSENI, B. 2017. Increasing the Use of Liquefied Petroleum Gas in Cooking in Developing Countries. *Live Wire: 2017/74; World Bank: Washington, DC, USA.*

Wang, C., Yi, W. K., Tao, Z. W., & Carovano, K. (1998). Photovoice as a Participatory Health Promotion Strategy. *Health Promotion International*, 13(1), 75–86. https://doi.org/10.1093/heapro/13.1.75

Wang, C., & Burris, M. a. (1997). Photovoice: Concept, Methodology, and Use for Participatory Needs Assessment. *Health Education & Behavior*, 24(3), 369–387. https://doi.org/10.1177%2F109019819702400309

Wang C, Redwood-Jones Y a (2001). Photovoice Ethics: Perspectives from Flint Photovoice. *Heal Educ Behav*;28:560–72. https://doi.org/10.1177%2F109019810102800504

WHO. 2014. *WHO Indoor Air Quality Guidelines: Household Fuel Combustion. Geneva: World Health Organisation; 2014. http://www.who.int/indoorair/guidelines/hhfc/en/)* [Online]. [Accessed].

WHO. 2016. *Burning Opportunity: Clean Household Energy for Health, Sustainable Development and Wellbeing of Women and Children. World Health Organisation, Geneva. http://apps.who.int/iris/bitstream/10665/204717/1/9789241565233_eng.pdf* [Online]. [Accessed].
